# Real-Time Electroanalytical Measurements of Dynamic Reaction Chemistry Provide Generalizable Mechanistic Considerations for Metal Nanoparticle Synthesis

**DOI:** 10.1021/jacs.5c19321

**Published:** 2026-01-30

**Authors:** Gabriel C. Halford, Abigail M. Sublett, Michelle L. Personick

**Affiliations:** Department of Chemistry, 2358 University of Virginia, Charlottesville, Virginia 22904, United States

## Abstract

The growth of metal nanoparticles is well understood to involve a combination of kinetic parameters as well as selective or nonselective passivation of surfaces by adsorbates. However, these influences are challenging to measure directly and in real time, which makes it difficult to define reaction mechanisms that are sufficiently detailed and specific to fully predict rather than retrospectively rationalize observed growth phenomena. In the present work, we demonstrate that open-circuit potential (OCP) measurements of the mixed potential of metal nanoparticle growth solutions represent a uniquely facile approach for directly identifying and understanding these complex chemical growth processes in an in situ and time-resolved manner. We combine OCP measurements of particle growth with point-in-time electron microscopy and elemental analysis to establish the power of OCP measurements even as a stand-alone approach. In doing so, we also uncover generalizable principles for synthetic design, such as the critical importance of precise changes in the chemistry of the growth solution at early time points and on short time scales in directing shape development trajectories, even when the faceted shape does not emerge until later in the growth process. Further, we validate the use of the mixed solution potential to directly distinguish between surface passivation and kinetic control of particle growth. Overall, although the chemical factors contributing to the mixed potential during particle growth are complex, a rich understanding of specific chemical mechanisms can be extracted from these OCP measurements.

## Graphical abstract

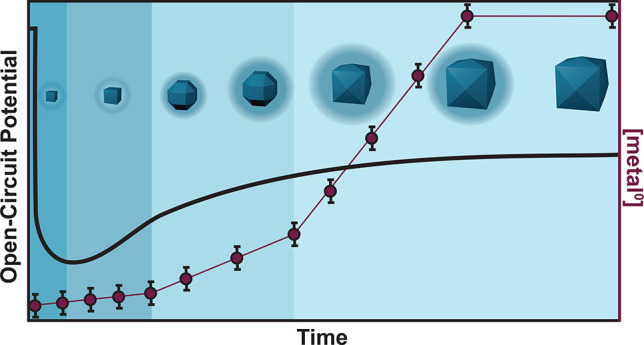

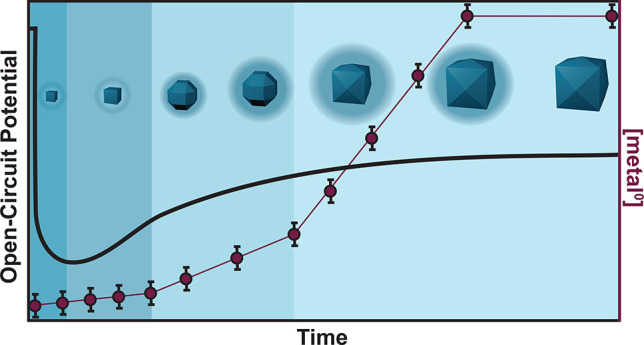

## 1 Introduction

Similarly to molecular structures, control of shape for nanoscale solids such as metal nanoparticles leads to different tunable properties and reactivity.^1–5^ However, unlike molecular synthesis, present principles of design for the synthesis of nanoparticles are less chemically detailed and specific and thus less broadly generalizable. This is largely because of difficulties in characterizing the chemistry and intermediates of nanoparticle synthesis due to the broad range of elements used, competing mechanistic processes, and interfacial reactions. Adding further complexity, local chemistry and/or side reactions influence the outcome of some nanoparticle syntheses, and the same chemical additives can play different roles in different synthetic systems.^6^ Despite a rich and detailed body of synthetic work, the prediction of the morphological results of synthesis is often limited to particular well-studied systems.^7–15^ Many significant advances in shape-selective nanomaterials synthesis have been made using systematic variation of synthetic conditions such as reagent concentrations, pH, or additives, and these observations have been well rationalized in terms of fundamental principles such as kinetic control and surface passivation.^5,8–28^ However, development of design principles that are both sufficiently fundamental to be translated across materials chemistries and also specific enough to be directly predictive is presently more limited. Principles for the prediction of reactivity and synthetic outcomes—analogous to steric hindrance, electronic effects, or nucleophilicity, but with additional consideration of the added complexity of reactions taking place at solid–solution interfaces—would help to meet challenges in the design of novel syntheses, especially for as-yet unachievable structure–composition combinations.^7,29,30^

One reason this challenge remains despite extensive work in this area is the limited number of tools for time-resolved, in situ study of colloidal metal nanoparticle growth. Though time-resolved characterization methods do exist, some of these—like ex situ electron microscopies, point-in-time inductively coupled plasma (ICP) spectroscopies, or point-in-time ultraviolet–visible (UV–vis) spectroscopy—do not provide in situ information about growth and have limited time resolution. Nuclear magnetic resonance spectroscopy, in situ transmission electron microscopy (TEM), and some in situ UV–vis kinetics techniques provide faster information acquisition but are challenging to employ and/or have only been well developed for a subset of noble metals.^7^ Additionally, many commonly used characterization techniques, such as electron microscopy, X-ray and electron spectroscopies, and diffraction, only provide physical or chemical characterization of the product nanoparticles, rather than information about the reaction chemistry during growth.^7,31^ Information, especially time-resolved and in situ information, about the changing chemistry of the nanoparticle growth solution during nanoparticle synthesis is therefore not commonly available. An approach for obtaining and interpreting this information would accelerate the development of a more mechanistically rigorous understanding of metal nanoparticle synthesis, enabling more precise and accurate synthetic predictions for a broader range of systems.

Recently, our group has pioneered the use of open-circuit potential (OCP) measurements as a real-time, in situ tool to monitor the chemistry of palladium (Pd) nanoparticle growth solutions during colloidal particle synthesis.^32,33^ Under metal nanoparticle growth conditions, the OCP is a mixed potential with contributions from the sets of half-reactions associated with metal ion reduction and reducing agent oxidation. Because both the synthesis and the OCP measurement are conducted in air under ambient conditions, the dissolved oxygen concentration and, in sufficiently reducing solutions, oxygen reduction both contribute to the OCP.^34–37^ Further discussion of the electrochemical fundamentals of OCP measurements is provided in the Supporting Information. Early results demonstrated a relationship between the reducing strength of particle growth solutions and the measured OCP.^32,33^ Additionally, the OCP technique has proven to be a versatile tool for multiple synthesis development applications, including translating between colloidal and electrodeposition syntheses^32,33^ and troubleshooting the role of trace chemical impurities in shape direction during colloidal synthesis.^33^ Our initial work validated OCP measurements as a source of real-time information about nanoparticle growth reactions that is meaningfully relevant to shape direction and showed that observations from OCP measurements correlate with known phenomenology of colloidal particle synthesis. Moreover, the OCP technique shows promise as a broadly implementable and accessible method for taking benchmark measurements of the real-time chemistry of both existing and newly reported nanoparticle syntheses for future comparison during synthesis development or troubleshooting.^7,33^ However, significant additional information is needed to understand which chemical phenomena *specifically* are captured by OCP measurements of metal nanoparticle growth and to exploit these detailed chemical insights to understand and/or influence particle growth mechanisms.

In the present work, we establish principles for the interpretation and deconvolution of these in situ OCP measurements of nanoparticle syntheses to extract detailed real-time information about the specific chemical processes that underlie nanoparticle growth as well as how these chemical processes dictate growth pathways and mechanisms. Using orthogonal time-resolved kinetic analysis and materials characterization techniques, we demonstrate that OCP measurements capture changes in metal ion reduction kinetics that are responsible for directing shape development before these shape differences are observable by electron microscopy. Additionally, we show that OCP measurements provide a uniquely rich understanding of the critical role of reagent diffusion to seeds early in particle growth in creating an optimal reducing environment. This is particularly notable given that reactant diffusion is known to be important in controlling nanoparticle growth,^38–42^ but it is difficult to explicitly use as a design parameter in colloidal metal nanoparticle synthesis development, in large part because there has not, to date, been a way to directly measure it. As a result, diffusion is more commonly used as a rationalization of synthesis results, rather than a design principle.^38–40,43^ We also validate the utility of OCP measurements in distinguishing between kinetic and surface passivation mechanisms for controlling nanoparticle shape—a determination that otherwise requires time-consuming analysis using a series of indirect characterization techniques—and which is crucial for understanding complex shape-control mechanisms.

With a robust understanding of the fundamental reaction processes that contribute to OCP measurements of metal nanoparticle growth, we use these in situ OCP measurements to probe outstanding questions of interest in the nanoparticle synthesis field. First, using OCP measurements of the mixed solution potential as a guide, we demonstrate that it is possible to redirect the growth pathway of a kinetically controlled nanoparticle synthesis *after* the initiation of growth, and to establish the time scale within which successful redirection is possible. We then use OCP measurements to identify contributions of side reactions during particle growth, specifically reducing agent degradation, which occurs on a synthetically relevant time scale. Together, this combination of principles for interpreting OCP measurements of nanoparticle syntheses and case studies of the application of this approach to outstanding synthetic questions establishes in situ OCP measurements as an experimentally facile but information-rich direct analytical method for developing a detailed and translatable understanding of the fundamental mechanisms behind the dynamic chemistry of metal nanoparticle growth.

## 2 Results and Discussion

OCP measurements are generally useful for comparing the reducing strength of particle growth solutions across multiple particle syntheses. The measured OCP, as well as how it changes over time, correlates with the formation of specific nanoparticle shapes. However, fully extracting and interpreting the detailed chemical information contained in the OCP measurements requires a deeper understanding of the relationships between the mixed potential, reagent consumption, and reaction kinetics. Probing these relationships is necessary to understand what the OCP measurement does and does not show about the reaction chemistry in real time. In particular, we were interested in understanding (1) whether the measured OCP can be used to report on metal ion reduction kinetics and (2) what information about reaction chemistry can be extracted from changes in the measured OCP over time.

Our group’s previously reported synthesis for Pd concave cubes (CC) and tetrahexahedra (THH) in cetyltrimethylammonium bromide (CTAB) surfactant solution was selected as a model system to establish these fundamental relationships because the synthesis is kinetically controlled and results in distinct changes in mixed potential over time for each product shape.^33^ CTAB surfactant from two MilliporeSigma product lines with slightly different impurity levels—BioUltra (99.0%, Lot No. BCCF7530—referred to as Lot “B” in our previous work)^33^ and BioXtra (99%, Lot No. SLCJ8356)—was used, and acetone and sodium iodide (NaI; “iodide”) were added as required for shape direction. For the successful synthesis of THH, the powdered CTAB surfactant must be oven-dried to remove excessive concentrations of solvent impurities before the preparation of the growth solution. As previously reported, when the as-received CTAB powder was used without pretreatment via oven drying and no acetone or iodide additives were introduced, the Pd particle products were not THH, but instead a mixture of CC particles and polydisperse twinned byproducts.^33^ The OCP of the CC growth solution was less positive (more strongly reducing) than the OCP of the THH growth solution throughout the reaction, leading to a previous hypothesis that the CC growth was faster.

### 2.1 OCP Measurements Provide Insight into Dynamic Changes in Reaction Kinetics

To identify what information OCP measurements can provide about particle growth kinetics, we used point-in-time inductively coupled plasma optical emission spectroscopy (ICP) analysis—which allows for direct, but ex situ, measurement of the kinetics of metal ion reduction—to correlate the reaction kinetics of Pd THH and CC formation with the OCP measurements of these reactions. To obtain point-in-time ICP kinetics data, an aliquot of nanoparticle growth solution was collected at each time point of interest and “quenched” with a chelating agent. The nanoparticles (metal solids) were then separated from chelated metal ions in solution and digested. ICP analysis was used to quantify the concentration of the metal(s) in each sample—representative of the total concentration of metal ions that have been reduced up to that time point. By tracking the increase in reduced metal concentration over time, these data can be used to build a kinetic curve.^7,13,20,44^ ICP data taken using filtered supernatant (containing Pd^2+^ ions) showed similar kinetics to data taken with digested metal solid samples and additionally indicated ∼90% consumption of Pd^2+^ ions across both CTAB sources (Figure S1 and Table S1).

Pd CC growth kinetics in BioUltra Lot B and BioXtra CTAB were tracked using ICP for a 1600 min (∼27 h) period, with samples taken most frequently in the first 3 h of growth (Figures 1A and S2, red and blue data points). For CC produced in both CTAB sources, the ICP data showed a rapid increase in reduced Pd in the first 7 min of growth to 2.5 ± 0.5 ppm of Pd for BioUltra Lot B and 2 ± 1 ppm for BioXtra (Figure S2). This period of fast growth was followed by an intermediate period of little or no Pd^2+^ reduction for ∼25 min and finally a mostly linear increase in reduced Pd until approximately 1000 min in both CTAB sources, representative of slower metal ion reduction at a constant rate. After ∼1000 min, no additional metal ion reduction occurred, indicating that particle growth is finished.

**Figure 1:**
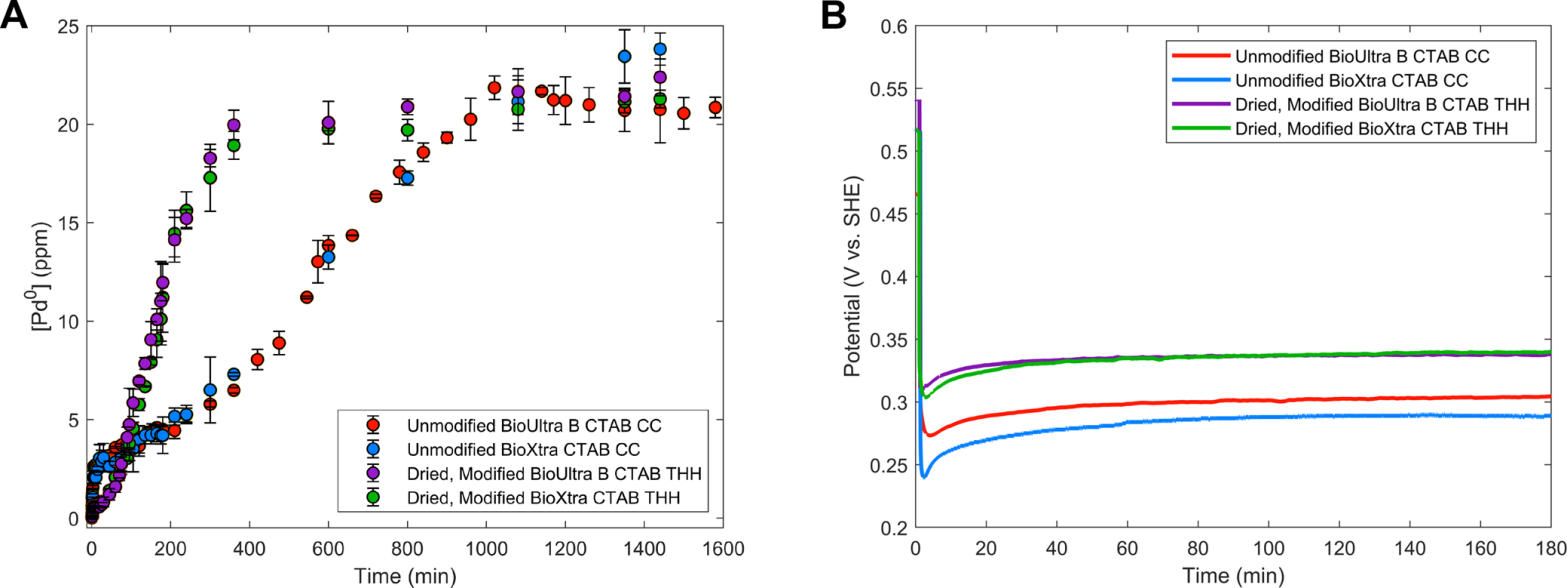
(A) ICP kinetics data showing the amount of reduced Pd (ppm) incorporated into nanoparticles over time (representative of the rate of metal ion reduction) for reactions that yield Pd CC (red, blue) or Pd THH (purple, green). (B) Corresponding OCP measurements of the same reaction conditions.

Correlation of time-resolved electron microscopy imaging of CC shape development with the ICP kinetics data shows that shape development happens during the third growth phase, where the rate of metal ion reduction is slower but constant (Figures S3–S6). TEM images indicate significant growth in size between 10 and 30 min in both CTAB sources (Figures S3 and S4). The TEM and scanning electron microscopy (SEM) images also demonstrate subtle differences in early growth between the two surfactant lines, with a wider size and shape dispersity of particles forming in BioXtra CTAB over the first 60 min (Figures S3–S6). At 120 min, particles in both CTAB sources had approximately doubled in size, retaining a truncated morphology (Figures S5 and S6). Only at 240 min did concave cubic structures emerge as the dominant product, along with slightly concave right bipyramids and other multiply twinned and poorly formed side products. At 360 min, CC growth was mostly complete. Because the ICP data show continued metal ion reduction until 1000 min but CC shape formation seems to have fully taken place by 360 min, we hypothesize that the additional metal ion reduction relates to shape focusing and/or the growth of one or more kinds of side product particles in the CC product mixture. The twinned and poorly formed side product particles are not as uniform from batch-to-batch as the CC, so conclusively determining when their growth is finished via SEM imaging is a challenge. The final percentages of CC and twinned side products differ in the two CTAB sources, with BioUltra CTAB samples containing ∼83% CC and ∼17% side products, vs ∼74% CC and ∼26% side products in BioXtra CTAB (Table S2).

Corresponding OCP measurements during the first 3 h of reaction—from our group’s previous work—show that, after an initial drop in solution potential due to the addition of reducing agent (ascorbic acid, AA), BioXtra Pd CC stabilized at a mixed potential that was 20 mV less positive (more strongly reducing) compared to that of BioUltra Lot B Pd CC (Figures 1B, S7 and S8).^33^ This difference in OCP is related to the slight difference in halide and organic impurity concentrations in each line of CTAB, primarily a higher concentration of acetone in as-received BioXtra CTAB,^33^ and is reflected in minor differences in particle quality and side product mixtures (Figures S3–S6 and Table S2). However, the overall similarities in ICP kinetics, shape development trajectory by point-in-time electron microscopy, and OCP measurements for the two CC syntheses imply that similar metal ion reduction kinetics, which ultimately drive shape development, lead to similar OCP measurements. In addition, although the OCP measurements for the two CC conditions are similar to each other, they are different from the OCP measurements for the growth of THH (Figures 1B, S7 and S8). Therefore, if OCP measurements reflect these reaction kinetics, we would expect the kinetics for the THH to be different from those for the CC, but identical between the two THH conditions.

To test this hypothesis, an identical analysis of ICP kinetics data and point-in-time SEM imaging was carried out for Pd THH (Figures 1A, S2, S9 and S10). The THH synthesis was probed in both CTAB sources: oven-dried BioUltra Lot B CTAB with 0.68 μM iodide and 0.26 M acetone added for shape direction (“dried, modified BioUltra Lot B CTAB”) and oven-dried BioXtra CTAB with 0.58 μM iodide and 0.31 M acetone additives (“dried, modified BioXtra CTAB”). In both cases, the rate of metal ion reduction for the first hour of the THH synthesis was slower than for the CC-forming conditions, with an especially stagnant rate of growth after the first 5 min. Only 1.5 ± 0.25 and 2 ± 0.5 ppm of Pd^0^ were measured after an hour of growth in dried, modified BioUltra Lot B CTAB and dried, modified BioXtra CTAB, respectively (Figures 1A and S2, green and purple data points). In contrast, a similar amount of metal ion reduction was measured after only 5–7 min for the CC (Figures 1A and S2). The metal ion reduction rate for the THH then increased in both CTAB sources, with a linear region of steeper slope occurring from 60 to 240 min. From 240 to 360 min, the rate of growth gradually leveled off, and at 360 min, growth was complete.

The syntheses of THH in the two CTAB sources not only had similar metal ion reduction kinetics but also similar shape development based on point-in-time SEM imaging, with small, faceted structures visible at 60 min. These structures grew into a pseudospherical morphology around 120 min and then developed a visible high-index pyramid feature on each face at 180 min (Figures S9 and S10). The THH increased in size in both CTAB sources until sometime between 360 and 600 min. This point-in-time imaging corresponds with the ∼360 min estimate for the completion of growth from the point-in-time ICP data. These similarities in the reduction kinetics and shape development are mirrored by the nearly identical OCP measurements for the two THH formation conditions (Figures 1B, S11 and S12).^33^ The two OCP traces are within 20 mV of one another for the first 20 min of measurement and then differ by less than 5 mV for the rest of the 3 h measurement period.

Time intervals where the slope changes in the OCP measurements correlate with regions of differing metal ion reduction rate from ICP. To more directly and quantitatively understand this correspondence, three regions of distinct slope were identified in each OCP measurement, and apparent rate constants (*k*_obs_) were calculated from the ICP data in these time intervals using pseudo-first-order reaction kinetics with respect to Pd^2+^ (Tables 1, S3–S4 and Figures S13–S14). Pseudo-first-order metal ion reduction kinetics have been reported for a variety of other noble metal nanoparticle syntheses with excess reducing agent.^25,45–48^ This data analysis confirms that the regions of different OCP slope do correspond to regions of differing reaction rate as described qualitatively in the preceding discussion. Specifically, there is an initial fast rate from 0 to 5 min that is more rapid for CC than for THH, an intermediate period of slow reaction rate from 5–30 min for CC and 5–60 min for THH where minimal metal ion reduction occurs, and, finally, a steady-state growth rate that is significantly slower for the CC conditions than for the THH conditions. In addition, particles with the same shape (CC or THH) have very similar rate constants at each stage and nearly identical *k*_obs_ during steady-state growth.

**Table 1:** Comparison between Measured and Calculated Rate Constants and OCP Slope for Growth of Pd CC and THH in 50 mM CTAB

reaction	region	range (min)	*k*_obs_ (min^–1^) measured	*k*_obs_ (min^–1^) calculated	slope_OCP_ (mV min^–1^) measured	slope_OCP_ (mV min^–1^) calculated
CC	1	0–6	0.0137	NS^a^	–3.3333	
unmodified	2	6–28	0.0004	NS	0.0545	
BioUltra B CTAB	3	28–180	0.0007	0.0007^b^	0.0373^c^	0.0393
						
CC	1	0–6	0.0129	NS	0.8333	
unmodified	2	6–45	0.0008	NS	0.5897	
BioXtra CTAB	3	45–180	0.0006	0.0010	0.0494	0.0402
						
THH	1	0–10	0.0035	NS	0.0000	
dried, modified	2	10–40	0.0007	NS	0.2667	
BioUltra B CTAB	3	40–180	0.0049	0.0118	0.0452	0.0703
						
THH	1	0–9	0.0026	NS	–0.7778	
dried, modified	2	9–50	0.0008	NS	0.4146	
BioXtra CTAB	3	50–180	0.0045	0.0101	0.0564	0.0747

a NS = no solution.

b Calculated value of *k*_obs_ is the average of fits to three OCP measurements.

c Measured values of Slope_OCP_ for Region 3 are the average of three OCP measurements.

While the mixed potential in OCP measurements is a combination of contributions from multiple half-reactions, if a single redox-active species is primarily responsible for the *change* in OCP, it may be possible to approximately model this change in OCP from *k*_obs_ or vice versa using the Nernst equation. A related approach has previously been used by others to evaluate enzyme kinetics from changes in the measured OCP using a redox mediator.^49^ In the present work, AA is the redox active species that is in the highest concentration, and the OCP increases (reducing environment gets weaker) over time. Therefore, we chose to probe whether the change in OCP could be approximated by considering the oxidation of AA in any of the time regions. The time-dependent Nernst equation for the change in potential due to the oxidation of AA (AA – 2e^–^ → AA_ox_) is shown in eq 1

1 $$E_{rel}\left(t\right)=\frac{RT}{nF}\ln\left(\frac{\Delta {\left[{AA}_{ox}\right]}^{*}\left(t\right)}{\Delta {\left[AA\right]}^{*}\left(t\right)}\right)$$

where *E*_rel_(*t*) is the relative value of the OCP after a period of time (*t*), *t* is time, *R* is the universal gas constant (8.314 J K^–1^ mol^–1^), *T* is temperature (313.15 K for this reaction), *n* is the number of electrons (2 for this reaction), *F* is the Faraday constant (96,485 C mol^–1^), Δ­[AA_ox_]* is the change in bulk concentration of oxidized AA, and Δ­[AA]* is the change in bulk concentration of AA.

The kinetics of the redox reaction between Pd^2+^ and AA were fit to pseudo-first-order in [Pd^2+^] and zero-order in AA (Figure S13), but the change in [AA] can be calculated from *k*_obs_ using the 1:1 stoichiometry between AA and Pd^2+^, leading to the following equations

2 $$\Delta {\left[AA\right]}^{*}={\left[AA\right]}_{i}-\left({\left[{Pd}^{2+}\right]}_{i}-{\left[{Pd}^{2+}\right]}_{i}e^{-kt}\right)={\left[AA\right]}_{i}-{\left[{Pd}^{2+}\right]}_{i}\left(1-e^{-kt}\right)$$

3 $$\Delta {\left[{AA}_{ox}\right]}^{*}={\left[{AA}_{ox}\right]}_{i}+{\left[{Pd}^{2+}\right]}_{i}\left(1-e^{-kt}\right)$$

4 $$E_{rel}\left(t\right)=\frac{RT}{nF}\ln\left(\frac{{\left[{AA}_{ox}\right]}_{i}+{\left[{Pd}^{2+}\right]}_{i}\left(1-e^{-kt}\right)}{{\left[AA\right]}_{i}-{\left[{Pd}^{2+}\right]}_{i}\left(1-e^{-kt}\right)}\right)$$

where [AA]_*i*_, [AA_ox_]_*i*_, and [Pd^2+^]_*i*_ are the concentrations of these species at the beginning of the time window of interest and *k* is the rate constant for the reduction of [Pd^2+^]. The derivative of eq 4 gives the slope of the potential, or the change in OCP over a period of time

5 $${slope}_{OCP}=\frac{RT}{nF}{\left[{Pd}^{2+}\right]}_{i}ke^{-kt}\left(\frac{{\left[AA\right]}_{i}+{\left[{AA}_{ox}\right]}_{i}}{\left({\left[{AA}_{ox}\right]}_{i}+{\left[{Pd}^{2+}\right]}_{i}\left(1-e^{-kt}\right)\right)\left({\left[AA\right]}_{i}-{\left[{Pd}^{2+}\right]}_{i}\left(1-e^{-kt}\right)\right)}\right)$$

where [AA]_*i*_ + [AA_ox_]_*i*_ is conveniently equivalent to the [AA] at the start of the synthesis, here 0.96 mM.

Using eq 5, it is possible to calculate *k*_obs_ in a particular time region from the slope of the OCP, assuming that (1) the oxidation of AA dominates the change in potential in that region and (2) the values of [Pd^2+^]_*i*_, [AA]_*i*_, and [AA_ox_]_*i*_ for the region are known or can be calculated. In this case, we worked from experimental [Pd^2+^] determined from ICP analysis of [Pd^0^] at the start of each time region. For regions 1 and 2, it is not possible to solve for a value of *k*_obs_ that satisfies eq 5 for the experimentally determined OCP slope, which suggests that the change in OCP in these two early regions is controlled by something other than, or in addition to, Nernstian behavior of AA. However, for the steady-state region, region 3, solving this equation based on the experimental slope in OCP results in solutions for *k*_obs_ that are physically reasonable (Tables 1 and S3–S6). The values of the calculated *k*_obs_ are similar in order of magnitude for the two CC syntheses. These values are an order of magnitude lower than the *k*_obs_ values for the THH, which are, in turn, similar to one another. The trends in order of magnitude for the calculated *k*_obs_ values generally track with those for the experimentally calculated values, although the exact values do not match. This preliminarily suggests that it might be possible to approximate the change in OCP during the steady-state region by Nernstian equations for the oxidation of AA over time.

We also used eq 5 in the reverse manner: to calculate a predicted OCP slope using the experimentally determined *k*_obs_ values from ICP (Tables 1 and S3–S6). This yields values for predicted changes in OCP over the steady-state region of ∼5.5 mV for the CC and ∼10 mV for the THH (Tables S4 and S6). These are in the same general range as what was measured experimentally for the change in OCP in this region (5.7–7.3 mV) based on an average of three OCP replicates per condition, providing further evidence that the change in OCP during the steady-state time frame can be approximated by the oxidation of AA. Together, these calculations identify promising prospects for directly deriving rate constants from the OCP data that would be enabled by enhanced experimental precision and improved modeling of the OCP data.

Viewing this data set as a whole, we can conclude that changes in the metal ion reduction rate are a significant contributor to changes in the mixed potential and that a single set of apparent rate laws for metal ion reduction may exist for formation of each faceted shape from a particular metal precursor under related classes of synthetic conditions (aqueous, low temperature, similar solution chemistry, etc.) This combination of ICP kinetics data and point-in-time image data also provides a more rigorous understanding of the time scale of shape development and completion of particle growth, as well as the relationship between the completion of particle growth and the measured OCP. We had previously assumed that CC and THH growth concluded within 3 h due to the near-constant mixed potential.^33^ However, the ICP data, which demonstrate continuing metal ion reduction for greater than 6 h, and the point-in-time SEM data, which show visible shape development until 4–6 h of reaction time, instead suggest that the nearly constant solution potential is indicative of slow, steady-state metal ion reduction. The majority of shape evolution occurs during this period. Conversely, although little shape evolution is visible by SEM in the first minutes of growth, significant early changes in the OCP and the metal ion reduction kinetics suggest that this is an important period for setting the reducing strength of the growth environment, which in turn determines the growth kinetics and shape trajectory later in the reaction. These observations correspond with literature reports of the importance of the early growth kinetics in determining product morphology.^41,50–53^ The origin and implications of these early changes will be discussed in Section 2.2.

To validate the proposed relationship between metal ion reduction rates and the measured mixed potential, OCP measurements and ICP kinetics data were analyzed for a set of synthesis conditions where changing the ratio of [Pd^2+^]/[AA] produces a gradient of different shapes (Figure 2). In addition to modifying reduction kinetics by tuning the concentration of shape-directing additives (or shape-directing impurities present in CTAB), the kinetics can also be adjusted by changing the ratio of reducing agent concentration to metal ion concentration. In this case, terraced cubes (TC, 0.38 mM Pd^2+^), THH (0.24 mM Pd^2+^), and CC (0.15 mM Pd^2+^) can be produced in dried BioUltra Lot B CTAB with 0.68 μM iodide and 0.26 M acetone additives while holding [AA] constant at 0.96 mM (Figure 2C–E).^33^ As expected, the overall ordering of mixed potentials showed that higher [Pd^2+^] (a lower [reducing agent]/[Pd^2+^] ratio) led to a more positive solution potential (weaker reducing environment) (Figure 2B). These shape gradient conditions were adapted from our previous work,^33^ but new OCP measurements and SEM images were taken, as we did not have a sufficient quantity of the CTAB lot used for the previously reported shape gradient and OCP measurements available for this study.

**Figure 2:**
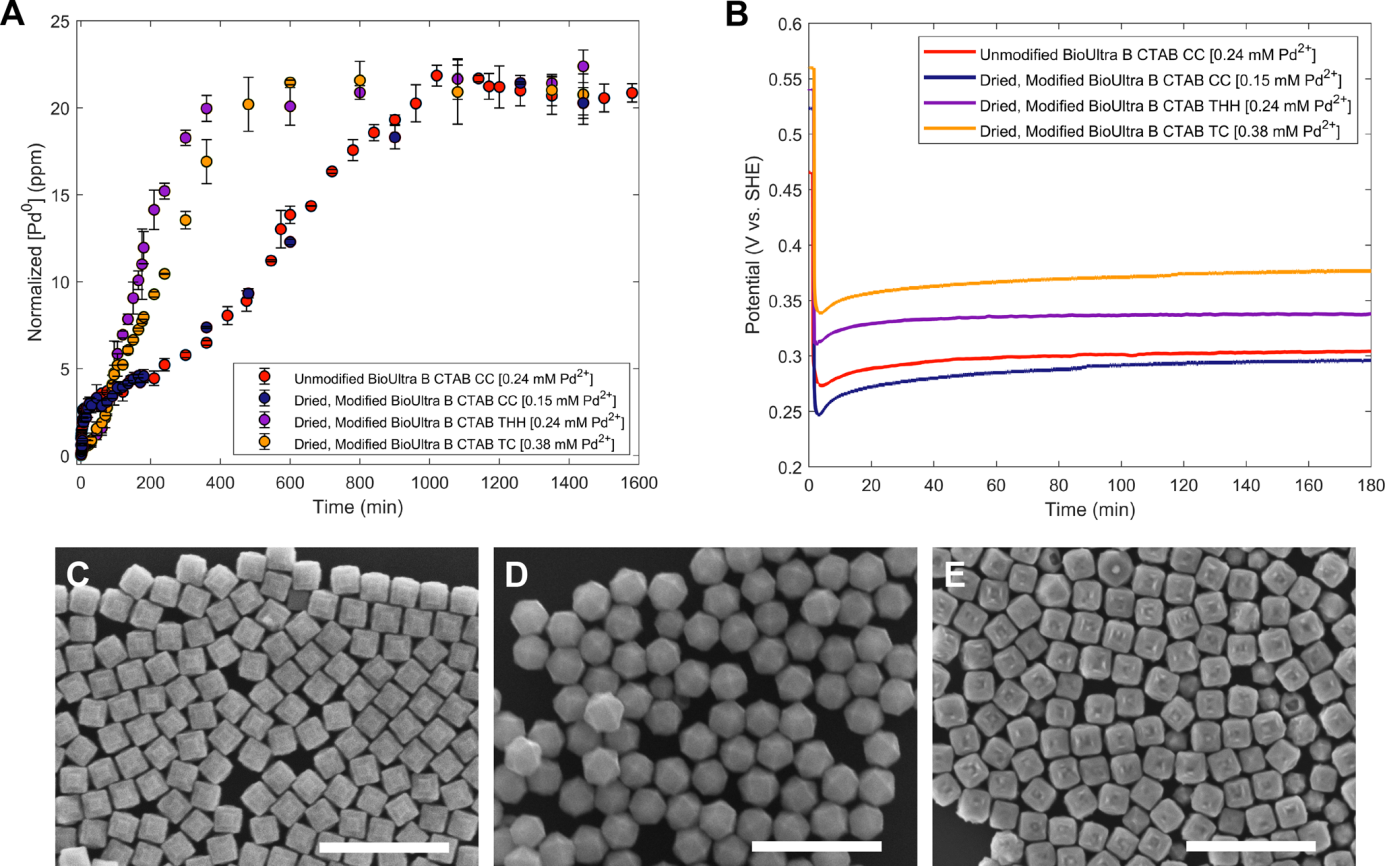
(A) ICP kinetics measurements, normalized by starting [Pd^2+^], and (B) corresponding OCP measurements of Pd nanoparticle growth in as-received 50 mM BioUltra Lot B CTAB with 0.24 mM Pd^2+^ in the growth solution and dried, modified 50 mM BioUltra Lot B CTAB with 0.15 mM, 0.24 mM, and 0.38 mM of Pd^2+^ in the growth solution. (C–E) SEM images of Pd nanoparticle shape as a function of metal ion concentration in dried, modified 50 mM BioUltra Lot B CTAB: (C) 0.38, (D) 0.24, and (E) 0.15 mM Na_2_PdCl_4_. Scale bars: 500 nm.

Comparison of ICP kinetics data across the [Pd^2+^]-mediated CC, THH, and TC shape series required accounting for the different absolute amounts of Pd salt precursor added to the growth solution by scaling for the pseudo-first-order kinetics. Higher [Pd^2+^] input led to higher [Pd^0^] incorporated into the nanoparticles at the end of the ICP time-course experiment (Figure S15). A normalization factor of $\frac{0.24}{x}$ , where *x* is equal to the millimolar (mM) concentration of Pd^2+^ initially added to the growth solution and 0.24 mM Pd^2+^ is the standard concentration of Pd^2+^ used to form THH, was introduced to account for the input [Pd^2+^] in each reaction (Figure 2A).

The rate constants for the CC condition (0.15 mM Pd^2+^) match those measured for CC synthesized in as-received, BioUltra Lot B and BioXtra CTAB with the standard 0.24 mM Pd^2+^ from the previous experiments (Figures 2A and S16, Tables 1, S3,S4,S7 and S8). The mixed solution potentials for the different CC syntheses are also comparable for the majority of the measurement, although there is a 25–50 mV difference during the first 30 min between the low [Pd^2+^] CC and the CC synthesized under standard conditions in BioUltra Lot B due to the higher concentration of acetone present in the [Pd^2+^] gradient experiments (Figure 2B). The low [Pd^2+^] CC have a “dented,” slightly concave–convex morphology (Figure 2E) and do not co-occur with twinned side products, while CC synthesized in unmodified CTAB have the appearance of smoother {*hk*0} facets. Despite these morphological differences, the correspondence of the ICP kinetics data for the different CC synthesis routes provides further evidence that there is a consistent rate constant for Pd ion reduction required to produce concave structures with this mixture of reagents (Tables 1, S3,S4,S7,S8, and Figure S17). Of note, the OCP measurement directly reflects the similarities in observed rate constants across the various CC synthesis conditions without requiring a normalization for initial [Pd^2+^].

The ICP kinetics data for the growth of TC particles with 0.38 mM Pd^2+^ were similar to those for THH (0.24 mM Pd^2+^) until 120 min (Figures 2A and S16). However, from 120 to 480 min, the TC metal ion reduction rate became slower than the THH metal ion reduction rate, and the growth of TC took ∼100 min longer to complete than THH growth (Figure 2A). The TC shape is low-index but closely related to the THH shape, with exposed {110} and {100} facets, as opposed to high-index {*hk*0} facets and {100} truncations seen for the THH.^33,54^ Given this relationship, it is interesting that the divergence in metal ion reduction rates occurs within the temporal region where the THH shape development—particularly of the high-index features—occurs, as observed through point-in-time SEM imaging (Figures S9 and S10). The difference in OCP between the TC and THH growth conditions from the very beginning of the reaction, however, again implies that the correct reducing strength/mixed solution potential needs to be present from early in the reaction in order to set optimal metal ion reduction kinetics.

Across the set of measurements described thus far, a less positive initial mixed potential correlates with a faster initial reaction rate. However, for these syntheses, the steady-state rate is generally observed to trend inversely with the initial rate: faster initial rates of reaction lead to slower steady-state rates and vice versa. While not all syntheses will have the same inverse correlation between initial reaction rate and steady-state reaction rate as the Pd CC and THH do, the importance of early growth kinetics to specific systems, such as the size distribution of spherical nanoparticles^41,50,53^ and the development of nanorod morphology and aspect ratio,^51,52,55^ has been reported in the computational and experimental literature. In addition, the above results show that initial concentrations of reagents do not necessarily directly correlate with the steady-state reaction rate, which is critical given the common use of the initial concentrations of metal ions and reducing agent (or their ratios) as an experimental handle for influencing particle growth rate, with higher ratios of [reducing agent]/[metal ions] predicted to increase reaction rate.

### 2.2 Diffusion and Establishment of Concentration Gradients at Early Time Points

#### 2.2.1 Depletion and Diffusion Effects on the Kinetics of Seeded Particle Growth

The process­(es) that determine the OCP at the beginning of the reaction arise from something other than the steady-state metal ion reduction, and these processes are crucial for setting the steady-state rate that drives desired shape formation minutes or hours later. Better understanding of the processes that contribute to the OCP in the first seconds to minutes—where the limit of detection for ICP kinetics, the spatial resolution for point-in-time electron microscopy imaging, and the time resolution for both techniques are inadequate—is required to understand why changes in the initial reducing environment, observable in OCP measurements, can be so crucial for growth kinetics and shape development later in nanoparticle synthesis reactions.

Of particular interest were the initial features in OCP measurements, which often show a dip in potential for ∼30 s to ≥30 min, depending on the synthetic system, before increasing by 10–20 mV and entering the steady-state growth region.^32,33^ The initial dip in potential correlates temporally with regions of rapid early metal ion reduction and subsequent stagnant metal ion reduction during the first ∼60 min of ICP measurements. While this feature could be a perturbation associated with turning off mechanical stirring—200 rpm stirring is used while adding reagents and then turned off 10 s after the addition of seeds and reducing agent—the minutes-long time duration of the potential dip makes that attribution unlikely.

One possible explanation for the early dip in potential is that it does relate to reagent mixing but that it results from a more complex reagent diffusion process than simply a change in the rate of reagent mixing due to mechanical stirring and its cessation. With the initial mixing of reagents following the injection of AA to the growth solution, a rapid rate of metal ion reduction onto the surface of the seed particles—autocatalyzed by the seeds^50,56,57^ —will develop. However, if the seed concentration is sufficiently high that the seeds are close to one another—and, therefore, proximal to the same pool of reactant molecules—reactants in the immediate vicinity of seeds will quickly begin to become depleted, leading to a stagnation of the metal ion reduction. This phenomenon is well established through both theory and experiment for the electrodeposition of metal nanostructures on flat substrates. Sufficiently close neighboring nucleation sites are known to “couple” and influence or limit reactant diffusion rates to one another during nanoparticle electrodeposition, in a process referred to as interparticle diffusion coupling, which has primarily been studied as a mechanism for particle size focusing.^58–65^ Similarly, theories of colloidal nanoparticle growth often predict a diffusion-driven size-focusing event following rapid reduction onto seed particles^41,42,66–68^ and generally accept that zones of local reagent concentration around growing colloidal seed particles differ from bulk concentration as a function of distance from the seed particle surface.^38–40,42^ As reagent depletion around seed particles occurs, reagent molecules that are farther away will diffuse to the lower-concentration areas near seed particles, in turn changing the mixed potential of the bulk solution. Concentration gradients will become established, and reactants will reach a steady-state rate of diffusion and reaction, as we observe. We note that, in colloidal synthesis, this model of nanoparticle growth is still controlled by the rate of metal ion reduction at the particle surface^41^ and is not truly *diffusion-limited*, but this rate is inherently dependent on diffusion kinetics because of their influence on the local reagent concentration.^41,42^

A multiphase diffusion regime, with early rapid reagent depletion, an intermediate phase of limited or no growth, and the subsequent establishment of a steady-state reagent diffusion rate during nanoparticle growth has been posited or noted experimentally for metal,^19,38–40,43,69–72^ metal oxide,^38,40,69,70^ and semiconductor^38,40,42,68–70^ colloidal nanocrystal growth, though this work has generally focused on controlling nucleation and size dispersity, not reaction rate or particle shape. Direct observation of reagent diffusion during colloidal particle growth is challenging and has been limited to a few model systems. So far, rigorous characterization of zones of local reactant concentration around nucleation sites during the early portions of growth has involved advanced techniques such as ex situ beamline small-angle X-ray scattering and X-ray absorption near-edge spectroscopy,^43^ in situ scanning transmission electron microscopy,^73^ or atomic force microscopy,^74^ and these studies have not directly monitored reagent depletion dynamics. Consequently, if OCP measurements do indeed capture these diffusion and depletion phenomena in real time, this would enable the direct use of these processes as mechanistic considerations in the rational design of metal nanoparticle syntheses.

To probe the role of reagent diffusion to and depletion around seeds in creating the initial dip in the OCP measurements, and thus determine whether this technique can measure the effect of these rapid processes, seed concentration was varied in the Pd THH synthesis in BioXtra CTAB. At higher seed concentrations, where seeds are closer together, we predicted that an accelerated early depletion of reactants would be visible as either a decrease in the depth or duration in the initial dip in OCP, as a more positive constant potential due to rapid depletion of reagents from the bulk solution, or as some combination of the two. Very low seed concentrations, in contrast, would be expected to result in less or no depletion of reactants around the widely spaced seed particles. This would be observable in the OCP measurement as an increase in the duration of the initial dip in potential and/or as a more negative steady-state potential due to the consistent presence of higher reducing agent concentrations in the bulk solution.

Manipulation of the seed concentration led to changes in both the measured OCP and the product morphology (Figure 3). The standard condition for THH formation is 100 μL of 10-fold diluted 22 nm Pd nanocube seeds.^33,75^ When 100 μL of undiluted seeds were used (10× the standard amount of seeds), the OCP was consistently 15–20 mV more positive than in the standard seed condition, with a shallower initial dip in potential, as predicted for more rapid reagent depletion (Figure 3A,B). Products were monodisperse TC rather than THH (Figure 3C) and were smaller than the THH that form with the normal amount of seeds (71 ± 5 nm vs 135 ± 10 nm). When 100 μL of 100-fold or 1000-fold diluted seeds were used (a 10× or 100× decrease in seed concentration, respectively, from standard conditions), the initial OCP of the 100-fold diluted seed sample was close to that of the standard condition, while the 1000-fold diluted seed condition began slightly lower in potential, indicative of slower early depletion (Figure 3A,B). The products were a mixture of poorly formed TC and truncated THH-like particles, resulting from a shift away from ideal reaction kinetics (Figure 3E,F).

**Figure 3:**
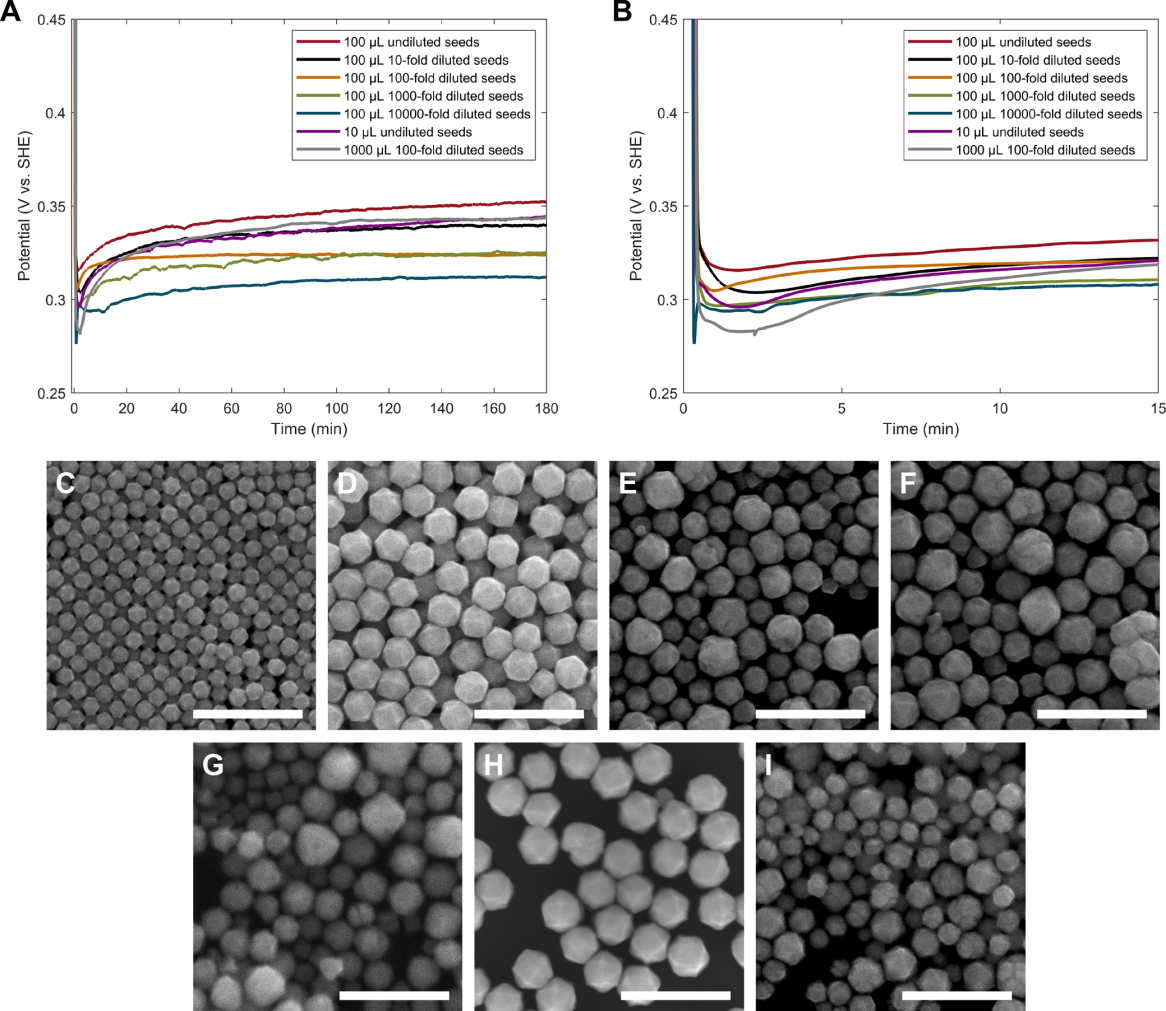
(A) OCP measurements of particle growth reactions in dried, modified 50 mM BioXtra CTAB (THH-forming conditions) with different seed concentrations. (B) Zoomed-in plot of the first 15 min of the OCP measurements shown in (A). (C–I) SEM images of Pd nanoparticle shape as a function of Pd nanocube seed concentration and/or volume for the conditions measured in (A,B): (C) 100 μL of undiluted seeds, (D) 100 μL of 10-fold diluted seeds (standard condition), (E) 100 μL of 100-fold diluted seeds, (F) 100 μL of 1000-fold diluted seeds, (G) 100 μL of 10,000-fold diluted seeds, (H) 10 μL of undiluted seeds, and (I) 1000 μL of 100-fold diluted seeds. Scale bars: 500 nm.

The OCP measurement for the most dilute seeding condition tested, 100 μL of 10,000-fold diluted seeds (a 1000× decrease from the standard seed concentration), exhibited a sharp and immediate down-and-up feature at the beginning of the reaction (Figures 3A,B and S18), in contrast to the smooth dip in potential observed in the first 10–15 min for all other seed concentrations tested. The products included a population of smaller cubes as well as truncated, poorly defined particles (Figure 3G). The sharp down-and-up feature early in the OCP measurement appears similar to our previously reported OCP measurements of a synthesis for homogeneously nucleated Pd cubes and octahedra, although those measured potentials then sloped upward in potential due to rapid depletion of AA, which was only in a 1.14:1 molar ratio with Pd^2+^.^32,76^ To provide a direct comparison of the above data to a known homogeneously nucleated synthesis, the [AA] in the cube and octahedra syntheses was increased to a 19:1 [AA]/[Pd^2+^] ratio to prevent rapid AA depletion. This yields smaller cubes and octahedra. The mixed potentials of these reaction conditions showed the same feature at the beginning of the measurement (Figure S19), confirming that a nearly instantaneous decrease and increase in the OCP at the beginning of a growth reaction is characteristic of homogeneous nucleation and fast depletion of reagents from the bulk solution due to rapid seed formation. Conversely, the smooth dip observed at the beginning of other OCP measurements is representative of seed-mediated growth. As a result, OCP measurements can help distinguish between these two types of early nanoparticle growth, which happen on short time scales that are inaccessible with many other analytical approaches.

We also investigated adding different volumes of the standard concentration of seeds as alterations to the total growth solution volume can affect reagent diffusion. Addition of 10 μL of undiluted seeds (1.01× the standard seed concentration, but a smaller volume) successfully produced THH (Figure 3H). Although the initial dip in the OCP with 10 μL of undiluted seeds was deeper, the solution potential of this condition rapidly reached a value within 5 mV of the standard THH OCP in the first 4 min of measurement (Figure 3A,B). Conversely, the addition of 1 mL of 100-fold diluted seeds led to a mixture of THH and other poorly formed products with high size dispersity (Figure 3I). This is also the same number of seeds as the standard conditions but at a lower overall concentration in solution (0.91× the standard seed concentration) and it introduces a large change (10%) in the total reaction solution volume from the standard conditions, altering the growth solution concentrations of other reagents as well. The initial dip for this condition was much larger in magnitude and lasted 15 min before reaching the ideal potential for THH formation (Figure 3A,B), indicating significant slowing of early reagent diffusion because of the greater solution volume and the dilution of the seeds.

To further probe the role of reagent transport to seeds in determining the early mixed potential behavior of seed-mediated growth, we examined the influence of the mechanical stirring rate at the beginning of the Pd THH synthesis in BioXtra CTAB (Figures S20 and S21). All reactions were stirred at 200 rpm at 40 °C prior to the addition of seeds and AA. The stir rate was then varied from the standard 200 rpm in subsequent steps. The results of these experiments confirm that the initial dip in potential early in OCP measurements of seeded syntheses relates to reagent diffusion to the seed particles, in this case driven by a combination of mixing and fast reduction of metal ions at the nanoparticle surface. Specifically, this dip in potential lasts longer when the initial phase of reagent diffusion is slower (no stirring or low stir rate) because the solution is not well mixed and diffusion relies more on mass transport through the growth solution, while the dip in potential is almost eliminated when reagent transport to seeds is facilitated by high rates of magnetic stirring (i.e., 800 rpm or vortex stir rates, or stirring lasting for minutes). In the latter case, mechanical mixing dominates over diffusion gradients resulting from the reaction at the nanoparticle surface. A range of intermediate stirring conditions—100–800 rpm for 10 s—allow for proper formation of THH particles, but the upper and lower extremes of stir rate and stir time significantly perturb particle formation (Figure S20). Constant stirring caused the OCP to fluctuate throughout the reaction, indicating that stirring after the first minutes of the reaction causes a continuous disturbance of the equilibrium diffusion around the growing nanoparticles and thus the metal ion reduction rate (Figure S21A). As a result of this continual disturbance of the reaction environment, the products formed under constant mechanical stirring were a mixture of low-index, truncated faceted shapes with significant size dispersity (Figure S21C).

In order for THH to form, there must be a sufficiently high rate of mixing in the initial seconds of the reaction to ensure uniform distribution of reagents, but mechanical stirring must be turned off during the first several minutes of the reaction to avoid perturbing the equilibrium diffusion rate of reagents that results from the surface reaction. This has important implications for synthetic reproducibility, as inconsistencies in swirling of colloidal reactions in scintillation vials^7^ and even in stir bar placement in magnetically stirred syntheses^77^ may cause irreproducibility of shaped particle products, especially when shape development is governed by kinetics. Given the ability to visualize changes in reaction kinetics due to reagent mixing and transport using OCP measurements, the technique could be used to report on ideal mixing conditions with the aim of developing more robust and informative protocols for the reproducibility of syntheses requiring “swirling” or mechanical stirring.

Considered together, these results show that OCP measurements capture the real-time changes in the mixed potential of the bulk solution early in the reaction that result from establishment of a concentration gradient and a diffusion–reduction equilibrium in solution. This is notable, since semi-direct, real-time observation of diffusion and especially of local reagent concentration around seeds is not easily achievable with common characterization techniques, despite the frequently proposed role of bulk diffusion effects in the design and understanding of nanoparticle synthesis.^40^ While seed concentrations are one way to manipulate reagent diffusion, chemical additives can also influence diffusion by altering both solution transport and the rate at which molecules can diffuse through surfactant layers to reach the surface of the nanoparticles to react. For example, modification of surfactant layer density and/or stability and a corresponding modulation of reagent diffusion is the proposed role of iodide and acetone additives in directing Pd nanoparticle shape from CC to THH.^33^ Being able to directly measure what an additive is fundamentally changing about the dynamic chemistry of the reaction solution will help to overcome challenges that arise from assuming that the same additives will have similar effects in different reaction systems. Reagent depletion around seed particles and subsequent reagent diffusion from the bulk solution also have significant implications for scale-up or scale-down of synthesis, where the total volume of the reaction vessel can influence diffusion and mixing, and direct measurement of these phenomena using real-time OCP measurements would aid in streamlined optimization of both processes to overcome challenges in this area.

#### 2.2.2 Shape Selection of Pd Tetrahexahedra due to Diffusion Limitations in High [CTAB]

To validate the above mechanistic claims about the importance of reagent diffusion to and depletion around seeds in setting growth kinetics and to illustrate the influence of chemical parameters on diffusion, we examined Pd nanoparticle growth in 150 mM as-received BioUltra Lot B CTAB—in contrast to the 50 mM CTAB used in the previously described Pd THH syntheses. Iodide (0.47 μM) was added for shape direction, but no additional acetone was needed for THH to form in this case, likely due to the higher concentration of the acetone impurity in the more concentrated CTAB. Interestingly, under these synthetic conditions, multiple initial concentrations of Pd^2+^—from 0.19 to 0.48 mM—led to formation of THH (70–90%) of a large, disperse size (183 ± 29 nm; Figures 4C,D and S22), which differs from the gradient of shapes produced at different [Pd^2+^] in 50 mM CTAB (Figure 2C–E). Decreasing [Pd^2+^] to 0.15 mM led to the formation of particles with both concave and convex features (Figure 4E). This difference suggests that in 150 mM CTAB, the high [CTAB] limits the rate of bulk reagent diffusion and, consequently, metal ion reduction—rather than the rate being determined only by the ratio of [AA]/[Pd^2+^]—and directs the formation of kinetically controlled products.

**Figure 4:**
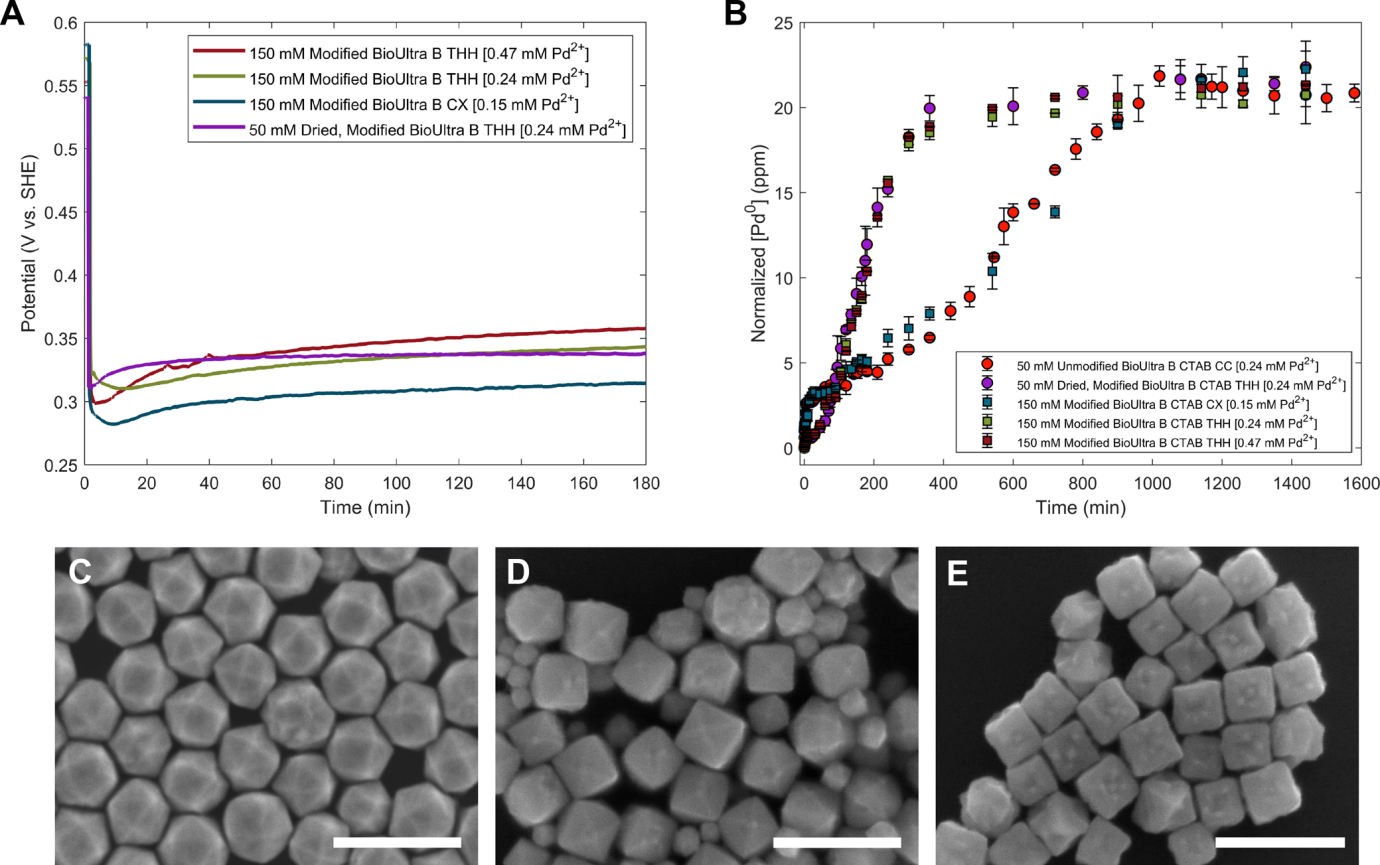
(A) OCP measurements of reactions in iodide-modified 150 mM BioUltra Lot B CTAB with 0.47 mM, 0.24 mM, and 0.15 mM of Pd^2+^ in the growth solution, along with dried, modified 50 mM BioUltra Lot B CTAB with 0.24 mM Pd^2+^ (THH) as a benchmark comparison. (B) ICP kinetics measurements for the same reactions, as well as unmodified 50 mM BioUltra Lot B CTAB with 0.24 mM Pd^2+^ (CC), all scaled by starting [Pd^2+^]. (C–E) SEM images of Pd nanoparticle shape as a function of Pd^2+^ concentration in 150 mM BioUltra Lot B CTAB: (C) 0.47, (D) 0.24, and (E) 0.15 mM Na_2_PdCl_4_. Scale bars: 500 nm.

To compare the Pd THH growth conditions in 150 mM CTAB to the previously described [Pd^2+^]-mediated shape gradient in 50 mM CTAB, we took OCP measurements of multiple growth solution compositions (0.47 mM, 0.24 mM, and 0.15 mM Pd^2+^) in 150 mM CTAB. Both of the THH-forming conditions (0.24 mM and 0.47 mM) in 150 mM CTAB produced OCP measurements that were similar to those for Pd THH formation in 50 mM CTAB (Figure 4A). The OCP trace for the growth solution in 150 mM CTAB with 0.15 mM Pd^2+^—which generates concave–convex (CX) Pd particle products (Figure 4E)—was approximately 40 mV less positive (more strongly reducing) compared to the measurements of THH-forming growth solutions (Figure 4A). It was, however, about 40 mV more positive than the solution potential for CC particles made with 0.15 mM Pd^2+^ in dried, modified 50 mM BioUltra B CTAB (Figure 2B). This aligns with our previous observation of concave–convex particles as an intermediate shape between THH and CC.^33^ Of note, the initial dips in potential in 150 mM CTAB were flattened and lasted for a longer time period than those seen in 50 mM CTAB, further establishing a relationship between low rates of reagent diffusion and this feature in the OCP measurements.

The role of CTAB in limiting diffusion was confirmed by OCP measurements of simple Pd particle growth solutions in different [CTAB] (BioXtra), ranging from 100 to 12.5 mM (Figures S23 and S24). These minimally complex growth solutions contained 10 mL of CTAB, 500 μL of 10 mM H_2_PdCl_4_, 100 μL of 1000-fold diluted small (≤5 nm) pseudospherical Pd seeds, and 100 μL of 100 mM AA. The measurement in 100 mM CTAB showed a ∼50 mV dip in potential for the first ∼15 min. The dip in potential lasts for 2 min in 50 mM CTAB, while OCP measurements of the same reagent mixture in 25 mM and 12.5 mM CTAB do not show the same dip in potential early on, instead maintaining a near-constant potential throughout the measurement.

Given that THH form at multiple [Pd^2+^] in the 150 mM CTAB synthetic system, we used ICP analysis to investigate whether there are consistent rate constants of metal ion reduction for these different synthetic conditions, which form the same shaped product—as was previously observed in the standard 50 mM CTAB (Figure S25). Two THH-forming conditions measured in 150 mM CTAB (0.24 mM Pd^2+^ and 0.47 mM Pd^2+^) indeed showed metal ion reduction kinetics similar to one another and to the standard THH-forming conditions in 50 mM CTAB (Figure 4B), as well as similar shape development by point-in-time SEM (Figures S26 and S27). The reaction with 0.15 mM Pd^2+^ in 150 mM CTAB, which produces CX, had very different metal ion reduction kinetics from the THH-forming conditions and instead mirrored those observed for related “dented” CC particles (0.15 mM Pd^2+^, different iodide and acetone modifications) in 50 mM CTAB and CC (0.24 mM Pd^2+^, no additives) in 50 mM CTAB, with some subtle differences between 200 and 800 min (Figures 4B and S28).

These high [CTAB] Pd THH and concave–convex particle syntheses provide further confirmation of our proposed mechanism for the interplay of reagent diffusion, reduction kinetics, and measured OCP, all in the same synthetic system. The diffusion limitations due to the high [CTAB] control the kinetics of particle growth in 150 mM CTAB, restricting the ability to finely tune kinetic products by changing [Pd^2+^] that was possible in 50 mM CTAB. The low diffusion rate can be observed in the flattened and extended initial dip feature in the OCP. The diffusion limitations set by the high [CTAB] also lead to similar OCP traces for reactions with 0.24 mM and 0.47 mM Pd^2+^, both of which form THH, despite the differences in [Pd^2+^]. These similar OCP measurements correspond to similar observed rate constants—an additional demonstration of the consistent rate laws for metal ion reduction observed for specific shaped products and the ability of OCP measurements to report on the relative rate constants (Tables S9,S10 and Figure S29). Additionally, for these high [CTAB] conditions, the Nernst equation based on AA consumption cannot be successfully solved for *k*_obs_ using the experimentally measured OCP slope during steady-state growth, confirming the importance of other factors, such as bulk diffusion limitations, in this case.

As a stand-alone technique, OCP measurements simplify understanding of reaction kinetics by enabling real-time, in situ insight into the dynamics of early nanoparticle growth and by identifying regions in which the reaction rate changes (Table 2). Specifically, we have identified that the value of the initial solution potential upon reducing agent and seed injection to the growth solution correlates with the initial metal ion reduction rate—which may or may not trend with the steady-state growth rate later in the reaction. The “dip” in the mixed potential relates to the speed of reagent transport through the bulk solution following initial rapid reduction and can also be used to distinguish between homogeneous nucleation and seed-mediated growth on large seeds. The shallower second portion of the dip in mixed potential corresponds with a decrease in the metal ion reduction rate due to decreased reagent availability during the establishment of concentration gradients from the bulk solution to the particle surface. The near-constant or low slope region in the OCP measurements represents steady-state growth, where a balance of reagent diffusion and surface reaction at the growing nanoparticle has been established. The timing and relative rates of reaction during each of these growth regions are in agreement with the results of a computational study on the growth of spherical nanoparticles under conditions where the surface reaction rate is limiting,^41^ further validating our interpretation. With this detailed set of principles for interpreting OCP data, OCP measurements can be used to specifically identify the fundamental chemical processes that determine nanoparticle growth kinetics in a reaction system, as well as to make robust and specific comparisons between interrelated syntheses.

**Table 2:** Summary of Typical Features of OCP Measurements and Their Interpretations

time (min)	feature	origin	parameters	interpretation
0	initial solution potential	reducing strength of reaction solution (Fermi level)	value	Lower (less positive) potentials correspond to a stronger reducing environment and a faster *initial* metal ion reduction rate; higher potentials correspond to a weaker reducing environment and a slower *initial* reaction rate
				
0–100 (variable)	change in potential from initial potential to steady-state potential (“dip”)	depletion of reagents around growing particles and subsequent diffusion to establish concentration gradients (also influenced by reagent transport from stirring)	duration	Shorter duration corresponds to faster reagent diffusion; longer duration to slower reagent diffusion
			depth	A deeper dip correlates with slower reagent diffusion; a shallower dip with faster reagent diffusion (or enhanced reagent transport due to stirring)
				
variable	steady-state potential region	establishment of steady-state reaction rate	onset timing	If the OCP has a constant slope from the initiation of the reaction, the reaction is not strongly affected by initial reagent diffusion, either because reagent mixing is rapid (such as due to stirring) or depletion is very slow (due to a slow reaction rate)
			change in potential	If the reaction can be approximated by Nernstian behavior (eq 5) during the steady state, a larger slope in the OCP will correspond to a faster rate of reaction

In light of the powerful information about the roles of seeding, diffusion, and reduction kinetics that can be obtained through OCP measurements—as well as its ease of use compared to many other tools for time-resolved study of nanoparticle growth—the technique is well poised to answer outstanding questions about metal nanoparticle synthesis and untangle complex mechanisms. The remainder of this work is dedicated to addressing unanswered or complex synthetic problems using OCP measurements as the primary or sole method of characterization and to showing specifically how the features described above can be used resolve these questions. In particular, we use the OCP technique to probe the following: (1) separation of the relative contributions of kinetics and surface passivation in nanoparticle growth; (2) dynamic control or redirection of synthetic conditions; and (3) measuring the influence of side reactions in nanoparticle synthesis.

### 2.3 OCP Measurements of Surface Passivation-Controlled Particle Synthesis

The development and interpretation of the OCP measurement technique have thus far focused on particle syntheses where shape is kinetically controlled. However, shape control can be achieved through other methods, such as surface passivation. Often, the shape-defining mechanism is a mix of both kinetic control and surface passivation. Given the fast information acquisition and experimental simplicity of OCP measurements compared to ICP kinetics measurements, the technique holds promise for efficient assignment of nanoparticle growth mechanisms as well as possible deconvolution of the relative contributions of multiple growth mechanisms in a particular synthesis.

One well-characterized system where shape is primarily controlled by surface passivation is the growth of (Ag)Au dilute bimetallic nanoparticles, where a submonolayer coverage of surface Ag controls shape.^13,78,79^ Addition of different concentrations of Ag^+^ to the growth solution leads to the formation of different products: {111}-faceted octahedra with 0.92 μM AgNO_3_ in the growth solution (1:500 ratio of [Ag^+^]/[Au^3+^]), {110}-faceted rhombic dodecahedra and bipyramids with 9.2 μM AgNO_3_ (1:50 ratio of [Ag^+^]/[Au^3+^]), {310}-faceted truncated ditetragonal prisms with 37 μM AgNO_3_ (1:12.5 ratio of [Ag^+^]/[Au^3+^]), and {720}-faceted CC with 92 μM AgNO_3_ (1:5 ratio of [Ag^+^]/[Au^3+^]). Importantly for the present work, previous ICP kinetics data demonstrated that while growth is slower in the presence of Ag^+^ than in its absence, identical Au particle growth rates are observed with any of the above concentrations of added Ag^+^, regardless of the shape that is ultimately formed.^13^ The Ag surface passivation of the Au particle growth in the above case is theorized to occur by an underpotential deposition (UPD) mechanism, a process resulting in the deposition of a submonolayer of Ag onto the Au particle surface.^13,78–83^ Ag UPD onto the growing Au particle is a form of facet-selective surface passivation. By depositing onto a particular facet—specifically a {110}, {*h*10}, or {*hk*0} facet—of the growing Au particle, the reduced Ag slows further Au deposition onto that facet, leading to retention of the facet in the final nanoparticle product.

To test whether it is possible to distinguish kinetic control and surface passivation during particle growth using OCP measurements, we measured the changes in the mixed solution potential under kinetic and surface passivation control of Au particle shape. OCP measurements with and without added Ag^+^ were taken to explore the region in which Ag^+^ concentration affects Au reduction kinetics, while different [Ag^+^] were studied to probe the region where selective surface passivation controls particle shape. For all syntheses, growth solutions contained 10 mL of 100 mM CTAC, 500 μL of 10 mM HAuCl_4_, 200 μL of 1 M HCl, and 0–100 μL of 10 mM AgNO_3_, to which 100 μL of 100 mM AA and 100 μL of 1000-fold diluted Au seeds (∼7 nm) in CTAC were added sequentially.

Though the OCP measurement technique is, in theory, amenable to any metal chemistry in the growth solution, previous work has been conducted with Pd particle syntheses. Determination of appropriate protocols for OCP measurements of Au particle growth was therefore necessary. In particular, the choice of reference electrode (RE) type was crucial for accurate measurement. Hg/Hg_2_SO_4_ was selected to avoid leaching of Ag^+^ from a Ag/AgCl RE.^33,84^ Triplicate OCP measurements of the Au-only synthesis with a Hg/Hg_2_SO_4_ RE demonstrated measurement-to-measurement consistency (Figure S30A). SEM images of measured Au particle syntheses show a mixture of {111}-faceted shapes (octahedra, plates, bitetrahedra) and nanorods along with other faceted particles with apparent exposed {111} and {100} facets (Figure S30B–D). This product mixture is similar in size, appearance, and distribution whether or not it is measured, confirming that the OCP measurement does not contaminate or significantly influence the synthesis.

To elucidate mixed solution potential behavior during particle growth under conditions of selective surface passivation-driven shape control, OCP measurements were conducted for each of the four Ag^+^ concentrations leading to different (Ag)Au particle shapes (Figures 5A and S31–S34). Notably, these measurements demonstrated a roughly identical steady-state solution potential of +0.47 ± 0.01 V vs SHE, regardless of the amount of Ag^+^ added to the growth solution. This mixed potential is shifted ∼120 mV more positive (less strongly reducing) than the solution potential observed for the measurements of Ag-free Au particle growth—consistent with a slower growth rate relative to the Ag-free conditions as measured via ICP kinetics (Figure 5A,B).^13^ SEM imaging following OCP measurements of the growth solutions confirmed the development of the expected particle morphologies with each concentration of added Ag^+^: octahedra, rhombic dodecahedra, truncated ditetragonal prisms, and CC (Figure 5C–F). The development of the full range of Ag-passivated Au-faceted shapes, despite the identical solution potential measurements for each condition, indicates that OCP measurements do not capture differences in growth pathways due to selective surface passivation processes such as Ag UPD on Au, unless these surface interactions also influence overall reduction kinetics. The identical metal ion reduction kinetics for all of the Ag-passivated Au particle shapes mean that the identical mixed solution potentials for each shape are still a response to reduction kinetics—but the reduction kinetics, in this case, do not drive differences in shape development (Figure 5B). Appropriate reduction kinetics (and thus OCP) are still required to establish a rate of growth where selective surface passivation by Ag can effectively control shape.

**Figure 5:**
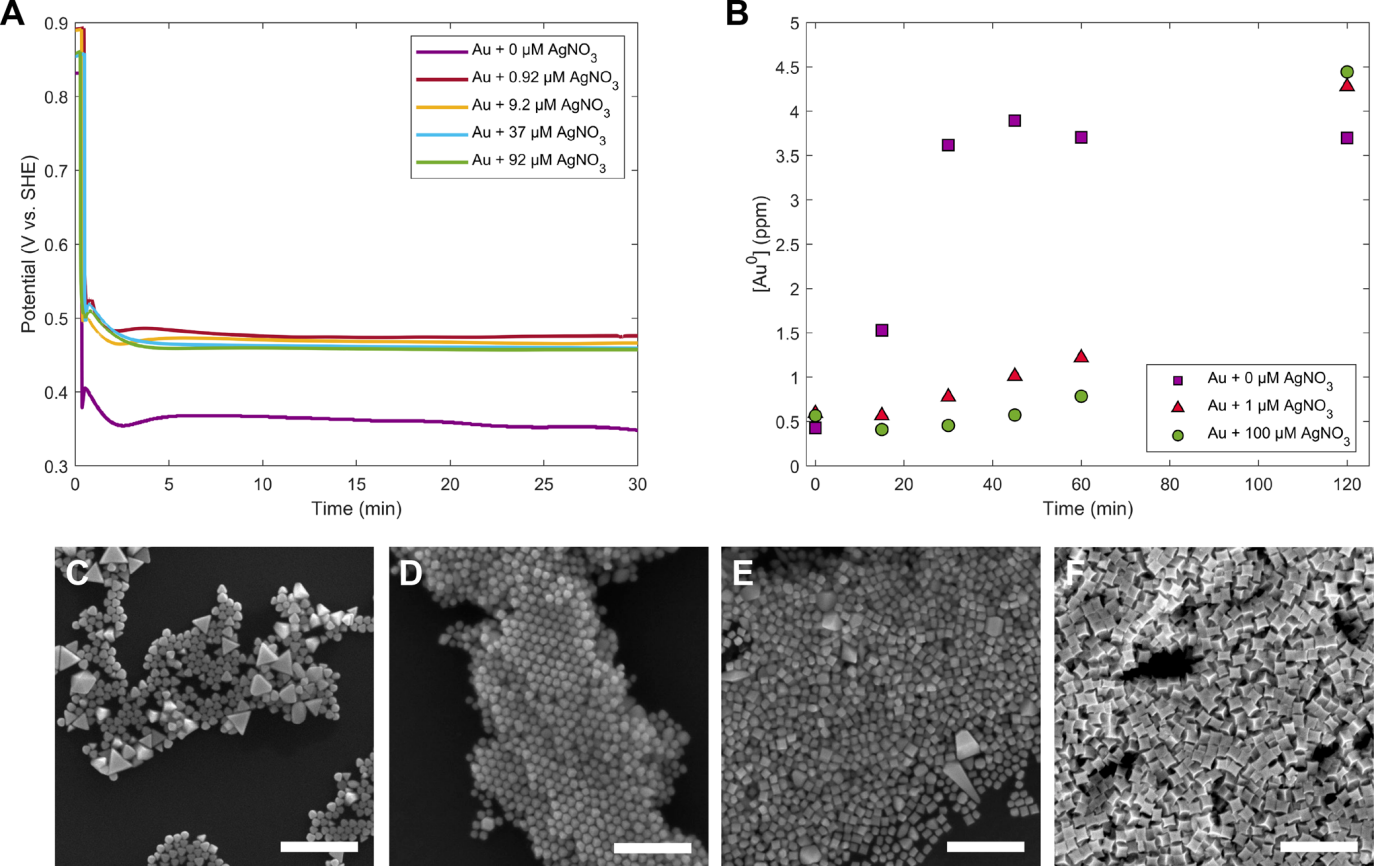
(A) OCP measurements of Au particle synthesis with different concentrations of Ag^+^ added for surface passivation-based shape direction. (B) ICP kinetics data for Au particle growth reactions containing 0 μM (purple squares), 1 μM (red triangles), and 100 μM (green circles) AgNO_3_. (C–F) SEM images of Au nanoparticle shape as a function of Ag^+^ concentration: (C) 0.92 μM, (D) 9.2 μM, (E) 37 μM, and (F) 92 μM AgNO_3_ in the growth solution. Scale bars: 500 nm. Panel B adapted with permission from ref 13. Copyright 2012 American Chemical Society.

The difference in the values of the OCP measurements for sets of different kinetically controlled shaped particle products versus the similarity in the values of the OCP measurements for sets of different selective surface passivation-controlled shaped particle products indicates that the OCP measurement technique can be used as a tool for distinguishing between these two mechanisms. Some surface effects, such as nonspecific surface binding (as opposed to selective passivation of particular facets) by halides, can have secondary kinetic effects on particle growth.^85,86^ Furthermore, single-crystal electrochemistry experiments have underscored the importance of halide adsorption and surfactant behavior at the surface–solution interface in controlling the comparative kinetics of metal ion reduction overall and to different surface facets.^87–89^ Surface effects with kinetic consequences can be observed in OCP measurements due to their influences on both reactant diffusion to the surface in general and the rate of reaction that results. The mixed solution potential could therefore possibly be used to distinguish the relative contributions of selective surface passivation and kinetic influences of particular additives, perhaps through concentration-dependent experiments. Other electroanalytical experiments focused on probing ligand adsorption to or the electrochemical potential at model metal surfaces or nanoparticle surfaces^87–90^ and computational work^87,89,91^ have been powerful tools for understanding reaction mechanisms at interfaces; these approaches may be helpful in tandem with future OCP experiments for uncovering the comparative influences of reagent diffusion and surface effects in particular systems.

### 2.4 Assessing the Feasibility of Mid-Reaction Redirection of Particle Growth Pathways

The importance of processes occurring on a variety of time scales—from seconds to minutes for reagent depletion around seeds, to hours for complete Pd particle shape development in CTAB—prompts questions about how early in a reaction the proper mixed solution potential must be set for targeted shape direction to occur successfully. This is an important consideration for the fine tailoring of particle synthesis, as it indicates when a growth reaction is possibly redirectable or modifiable with solution potential-altering additives before the growth pathway becomes fixed. A similar question has been considered in the case of altering the aspect ratio of Au nanorods during growth, monitored via time-resolved UV–vis spectroscopy.^52^ However, the OCP technique allows for observation of growth kinetics in systems where UV–vis monitoring is not feasible (i.e., growth of nanoparticles without a visible-range plasmon resonance or easily monitored metal ion precursor, such as Pd nanoparticles).

We used a familiar system—Pd particle synthesis in as-received 50 mM BioUltra Lot B CTAB—to investigate when it is possible to “redirect” from one anticipated shaped particle product to another. We added 0.068 M acetone and 0.68 μM iodide to CC-forming growth solutions in as-received BioUltra Lot B CTAB at various time points to attempt to “shift” products to THH *after* the initiation of particle growth with seeds and AA (Figure 6). These additive concentrations in as-received (not dried) BioUltra Lot B CTAB have metal ion reduction kinetics similar to those in the dried, modified BioUltra Lot B CTAB THH-forming condition (Figure S35). For each growth solution, diluted Pd nanocube seeds were added first, followed by 100 μL of 100 mM freshly prepared AA, and the addition of AA was taken as *t* = 0 min. All growth solutions—which did not yet have added acetone or iodide—had early solution potential behavior similar to that of OCP measurements of the typical CC growth solution (Figure 6).

**Figure 6:**
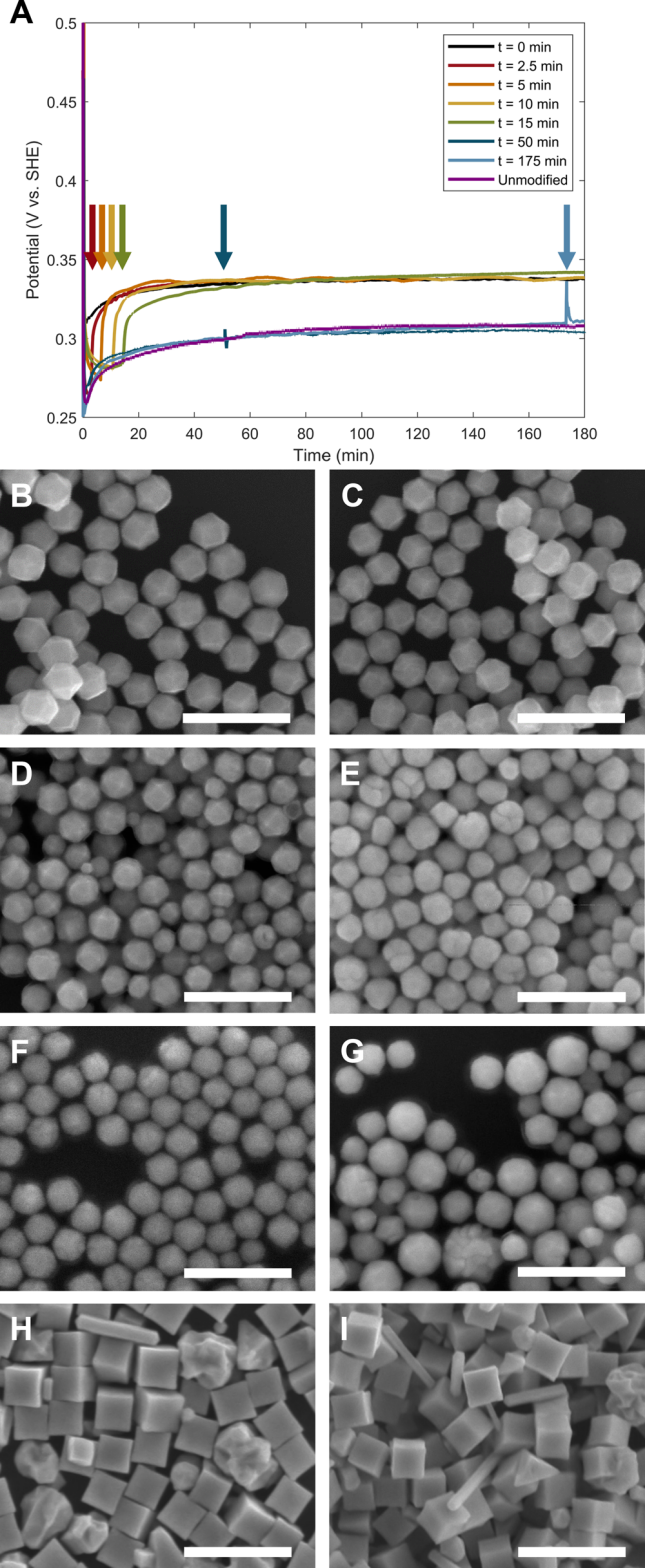
(A) OCP measurements of Pd nanoparticle growth reactions in as-received 50 mM BioUltra Lot B CTAB with acetone and iodide additives injected at various time points following the initiation of particle growth, in an attempt to shift growth from CC to THH. Arrows indicate the time of additive addition. (B–I) SEM images of Pd nanoparticle shape as a function of the time of additive addition: (B) *t =* 0 min, (C) *t =* 2.5 min, (D) *t =* 5 min, (E) *t =* 10 min, (F) *t =* 15 min, (G) *t =* 50 min, (H) *t =* 175 min, and (I) no acetone or iodide added. Scale bars: 500 nm.

Despite the fact that the CC and THH shapes take between 3 and 4 h to emerge based on point-in-time SEM imaging, acetone and iodide additives must be present in the growth solution by *t* = 5 min for well-formed THH to develop (Figure 6A,C,D). When acetone and iodide are added within the period of *t* = 10–15 min, a significant increase in mixed potential occurs—possibly indicating the additives’ influence on diffusion and/or metal ion reduction kinetics during that period of particle growth (Figure 6A). However, the formation of rounded structures at those two time points shows that the shape-directing additives must be present before 10 min of reaction to set appropriate growth kinetics for the formation of high-index features (Figure 6E,F). The slope of the steady-state OCP region for additive injection at *t* = 15 min is clearly different from the slope for the standard *t* = 0 condition, reflecting this detrimental change in reaction kinetics. When acetone and iodide are injected at 50 min, little or no change in the mixed solution potential occurs, but CC product formation is perturbed—indicating some intermediate regime where the additives can redirect the CC growth but cannot completely shift to THH growth kinetics (Figure 6A,G). No redirection of shape is possible when acetone and iodide are added at *t* = 175 min, although the CC shape has not yet fully formed by that time (Figures 6H, S5 and S6). The growth pathway for these kinetically controlled products, therefore, seems to be set in the first several minutes of the hours-long growth reaction, but early redirection of the growth pathway is possible. This is congruent with the observation of a 10 min window in which Au nanorod aspect ratio can be successfully changed using Ag salt additives, although the mechanism for shape control in that case is likely different.^52^ Redirection of growth through manipulation of the solution potential at later time points would likely require addition or removal of metal ions or reducing agent.

These experiments also provide further evidence that changes in solution potential due to the addition of acetone and iodide additives are because of indirect or direct changes they cause in metal ion reduction kinetics. The solution potential increases instantaneously with the addition of acetone and iodide at early time points in the reaction (i.e., *t* ≤ 5 min), when growth is also successfully redirected from CC to THH products. The timing of these successful product redirections corresponds with the early (*t* = 6–10 min) change in metal ion reduction rate during CC and THH growth (Figures 1A and S2; Tables 1, S3 and S4) and with the maximum time for which mechanical stirring can be applied and still allow for THH formation (*t* < 5 min; Figure S21A,B), indicating an early window in which growth kinetics are more flexible before the steady-state rate of metal ion reduction must be established for appropriate product formation. When acetone and iodide are added later (i.e., *t* = 175 min), their addition neither significantly influences the particle shape *nor* changes the mixed potential. If the increases in the mixed potential were only due to the presence of acetone and/or iodide species in solution, the mixed potential would always increase with their introduction regardless of whether they proceeded to have any shape-directing impacts. Instead, the solution potential only changes when the acetone and iodide additives are introduced early enough to lead to THH product production or to pseudospherical particles (*t* ≤ 15 min). The kinetic influence of these additives could relate to effects on bulk diffusion in solution early in the growth process—possibly by allowing additional reagent diffusion to the particle surface—but it is more likely related to influences on the accessibility of diffusion to the growing particle surface through combined effects of iodide surface binding and acetone intercalation between ordered CTAB molecules at the particle–solution interface.^33,85,86,92,93^ The latter would be an example of a surface effect that alters both growth kinetics and the OCP.

### 2.5 Identification of Side Reactions and the Conditions at Which They Influence Particle Growth

In our initial validation of OCP measurements through analysis of Pd nanoparticle syntheses with various common reducing agents in a simplified model system, we noticed a difference in the dynamics of OCP measurement over time for AA compared to hydroquinone, even though the reducing strengths of these reagents are relatively similar.^32^ Specifically, for AA, an OCP measurement of a Pd nanoparticle synthesis with 2:1 [AA]/[Pd^2+^] in the halide-free surfactant cetyltrimethylammonium hydrogen sulfate (CTAHSO_4_) showed an unusual line shape, going through an inflection point at around 15 min (Figures 7 and S36). This inflection point is reproducible and occurs significantly later than the initial dip for processes associated with reagent diffusion and depletion, which takes place in the first minute of this reaction. No inflection point is observed for the reaction with hydroquinone as a reducing agent under otherwise identical conditions. We sought to understand the origin of this inflection point by probing how it changes as a function of the initial [AA] and of the [AA]/[Pd^2+^] ratio.

**Figure 7:**
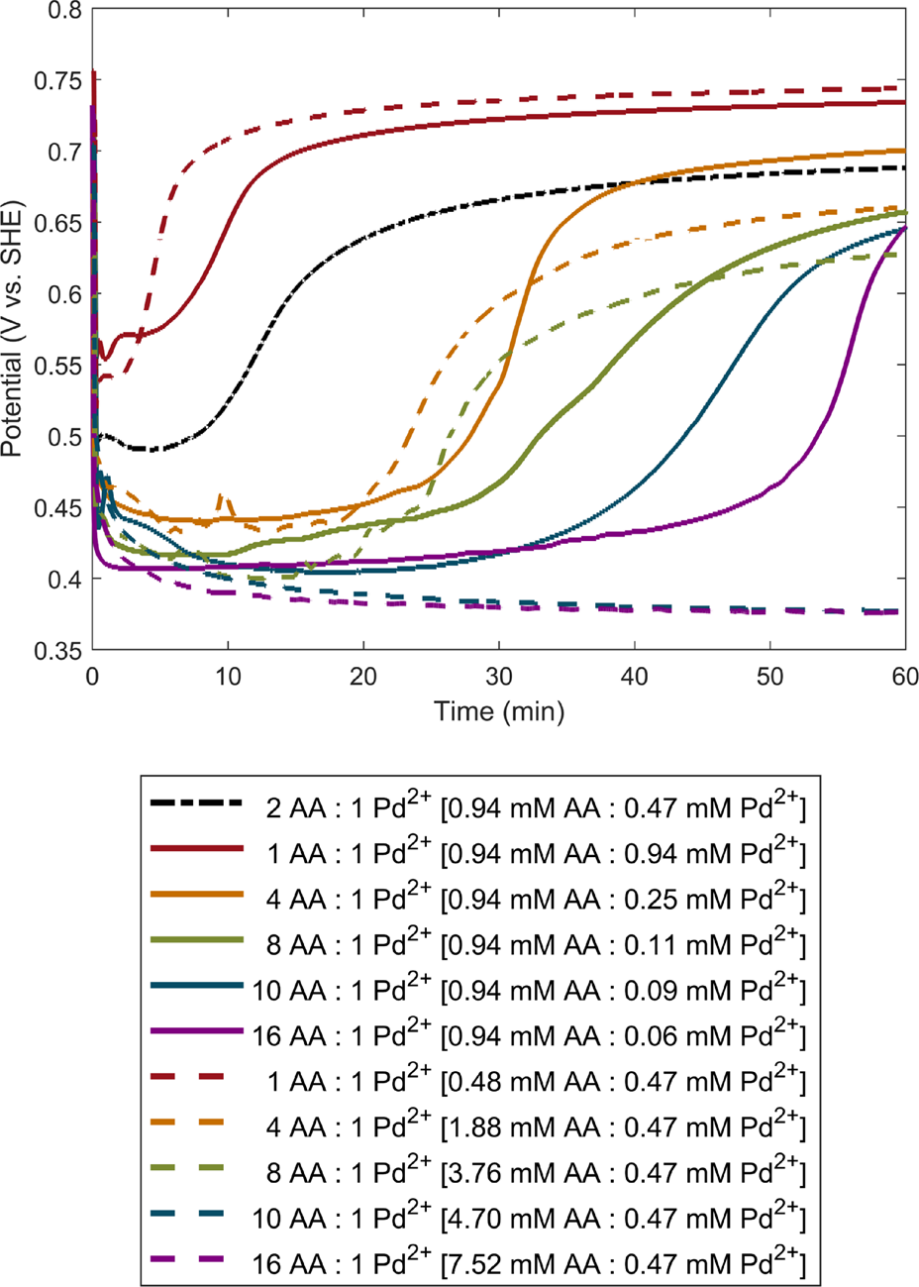
OCP measurements of Pd nanoparticle synthesis reactions with different ratios of AA to Pd precursor, with the ratio controlled by changing [Pd^2+^] or [AA].

All growth solutions were prepared with 10 mL of 100 mM cetyltrimethylammonium hydrogen sulfate (CTAHSO_4_; Tokyo Chemical Industries Lot No. 46XTG-ZG), 10 mM H_2_PdCl_4_, 100 mM freshly prepared AA, and 100 μL of 1000× diluted small, pseudospherical Pd seeds. Reactions were heated to 40 °C, with stirring at 200 rpm until 10 s after the addition of seeds. Volumes of 500 μL of 10 mM H_2_PdCl_4_ salt (= 0.47 mM in the growth solution) and 100 μL of 100 mM freshly prepared AA (= 0.94 mM in the growth solution; a 2:1 [AA]/[Pd^2+^] ratio) were taken as “standard” conditions, and all other measured conditions for this set of experiments iterated around these. Due to the halide-free surfactant, fast particle growth led to small, pseudospherical, and rounded octahedral Pd particle products (Figures S37B–G and S38B–G) rather than well-faceted or high-index structures.

When [AA] was held constant and [Pd^2+^] was modulated, the 200–250 mV rise in potential with inflection point was observed at all [AA]/[Pd^2+^] ratios between 1:1 and 16:1 (0.94 mM freshly prepared AA and between 0.94 and 0.0057 mM H_2_PdCl_4_). As [AA]/[Pd^2+^] increased due to a decrease in [Pd^2+^], the mixed potential early in each measurement became less positive (more strongly reducing), while the increase in potential and inflection point occurred later in the measurement (Figure 7, solid lines). OCP measurements of the same set of [AA]/[Pd^2+^] ratios, but with [Pd^2+^] held constant at 0.47 mM and [AA] modulated, revealed similar trends for the conditions with lower excesses of AA (Figure 7, dotted lines). For [AA]/[Pd^2+^] of 1:1–8:1 (0.48 mM to 3.76 mM AA and 0.47 mM H_2_PdCl_4_ salt), the rise and inflection point in the OCP also occurred progressively later as the [AA] was increased. When the [AA]/[Pd^2+^] ratio was 10:1 or 16:1 and [AA] was high (4.7 mM or 7.52 mM), no inflection point in the mixed potential was observed, even when the measurement was extended to 180 min (Figure S39). To investigate the dramatic difference between the OCPs for 8:1 and 10:1 ratios of [AA]/[Pd^2+^]— the 8:1 [AA]/[Pd^2+^] condition goes through an inflection point at 28 min, while a rise in potential is not observed over the course of a 180 min measurement for 10:1 [AA]/[Pd^2+^]— the OCP for a 9:1 [AA]/[Pd^2+^] condition was also measured. It showed a ∼10 mV increase in potential after about 50 min of measurement, indicating the possibility of a delayed inflection point in the OCP (Figure S38A).

The appearance of the rise in potential and inflection point when a high ratio of [AA]/[Pd^2+^] is established by decreasing [Pd^2+^], but not at the same ratios when [AA] is high, suggests that higher concentrations of excess AA inhibit this increase in the solution potential. Point-in-time ICP studies also indicate that for growth conditions with 2:1 [AA]/[Pd^2+^], Pd^2+^ reduction had concluded after 10.5 min, with the majority of particle growth occurring within the first 4 min (Figure S40). Since the OCP for this condition does not begin to increase until 10–15 min into the measurement—after particle growth is complete—the rise in potential is not attributable to any process occurring as a consequence of the redox reaction between AA and Pd^2+^.

The unexplained rise in potential and inflection point in the OCP measurements for the model reactions of Pd^2+^ with AA in CTAHSO_4_ (Figure 7) suggests that while OCP measurements primarily capture diffusion of reagents (to growing particles) and rates of metal ion reduction, the mixed potential can also be used to identify additional phenomena, such as background reactions involving redox-active species. Consideration of side reactions is especially important for particle growth reactions with AA; recent work has proposed a more complex pathway of AA oxidation chemistry in noble metal nanoparticle growth solutions, asserting that AA degrades in aqueous Au and Ag nanoparticle growth solutions at basic pH on a time scale relevant to common nanoparticle syntheses and that the degradation is to an as-yet unknown species other than dehydroascorbic acid (DHA), in contrast to previous assumptions in the literature.^94^ Given the possible mechanistic importance of AA degradation during synthesis, we used OCP measurements to directly monitor AA degradation over time as a function of its concentration.

Freshly prepared aqueous AA solutions with concentrations ranging from 0.1 mM to 1 M, adjusted with HNO_3_ to the pH of the model Pd particle growth solutions (pH = 1.5), were monitored by OCP for 20 h (Figure 8). All measurements in this series were taken at 40 °C with no stirring for the duration of the measurement. Most measurements of the OCP of fresh AA solutions showed an increase in potential over the 20 h measurement—indicative of the mixed potential growing less reducing, possibly due to AA degradation through reaction with dissolved oxygen. The timing and magnitude of this increase were concentration-dependent, with more dilute AA solutions experiencing a more rapid increase in potential earlier in the measurement period. The highest concentration tested, 1 M, did not notably increase in potential (Figure 8). A set of identical solutions was prepared, stored in the dark for 1 week, and then monitored with OCP to examine the behavior of “aged” AA. The aged AA underwent a color change from colorless to pale yellow, indicating degradation. After aging, several of the concentrations measured—0.1 mM and 1 mM aged AA—had reached the same mixed potential (+0.44 ± 0.01 V vs SHE), while the OCP of 10 mM aged AA also increased to this value over the course of the 20 h measurement. The OCP of 100 mM aged AA was constant at roughly the same value as the terminal potential for the fresh 100 mM AA measurement (0.37 V vs SHE).

**Figure 8:**
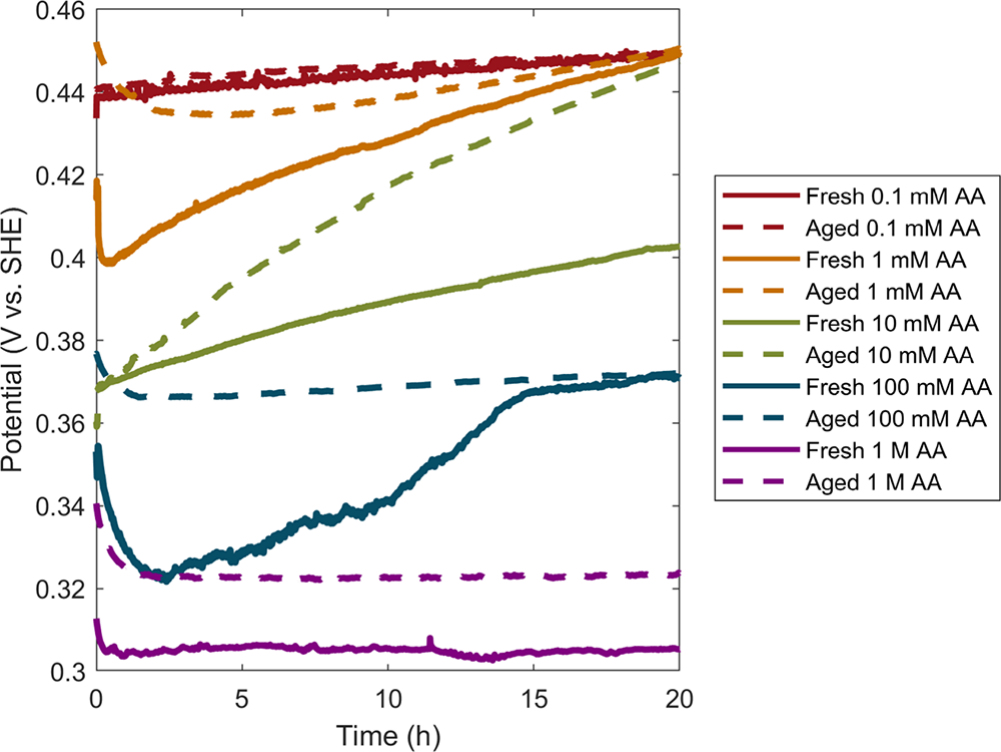
OCP measurements of fresh and aged AA in acidic, aqueous solution. The mixed potential and time of onset of AA degradation are [AA]-dependent.

These data suggest that the degradation of AA into a weaker reductant could be observable in the mixed potential during measurements of particle growth, that its degradation kinetics are concentration-dependent within concentration ranges relevant to nanoparticle synthesis, and that the time scale of degradation is sufficiently rapid to be relevant for Pd particle synthesis under acidic conditions. The concentration dependence of this degradation also explains other trends in the observed OCP behavior with different ratios of [AA]/[Pd^2+^], depending on whether AA is in high concentration (with constant [Pd^2+^]) or at a fixed moderate concentration (with low [Pd^2+^]). Higher absolute concentrations of AA remaining in solution—either due to a higher initial concentration of AA or to a higher excess of AA resulting from the absolute [AA] compared to [Pd^2+^]—slow the degradation process.

The attribution of the rise in solution potential to oxidative degradation of AA was further confirmed by an OCP measurement of the “standard” 2:1 [AA]/[Pd^2+^] model reaction under an argon (Ar) atmosphere (Figure S41). The initial solution potential is shifted ∼100 mV less positive—most likely due to the removal of the contribution from oxygen itself from the mixed potential—and a rise in potential is not noted, indicating that degradation of AA does not occur when the concentration of dissolved oxygen in solution is decreased. Interestingly, when syntheses using the standard Pd CC and THH growth conditions in 50 mM BioUltra B CTAB were conducted under Ar, the OCP for both reactions was shifted ∼100 mV less positive as well, while products of both syntheses were slightly polydisperse THH (Figure S42). Numerous factors with likely implications for reaction kinetics, including the reduction potential of Pd ions^95^ and the ease of oxidation of AA, are altered in the absence of oxygen, so the precise reason for the change in product morphology cannot be pinpointed without further experiments. However, these results underscore the significant impact of the dissolved oxygen concentration on both the mixed solution potential and the shape development, indicating that this could be a fruitful area for future study with OCP.

As a counterpoint to OCP measurements of AA degradation, we also investigated the mixed potential of fresh and degraded HQ solutions under ambient conditions (Figure S43). Changes in the OCP of pure HQ solutions began within 20–30 min. However, there is no inflection point in the mixed potential of Pd particle growth solutions with HQ as a reducing agent, except at the lowest [HQ] tested of 0.48 mM (Figure S44–S46). This is an order of magnitude lower concentration than the onset of measurable degradation for [AA] (∼4.5 mM), and the inflection point occurs later for HQ (∼70 min). Together, this suggests that the degradation of HQ in Pd nanoparticle growth solutions is slower than that of AA and/or that it takes longer for HQ to reach a concentration where it experiences rapid oxidative degradation. This is likely because the decrease in [HQ] via reaction with Pd^2+^ is slower due to the slightly weaker reducing strength of HQ compared to that of AA.

The above results indicate that OCP measurements can be used to detect side reactions such as reagent degradation but that contributions from background reactions may be masked when the reducing agent is in large excess. However, OCP measurements can also identify conditions under which reagent degradation may influence particle growth, such as low [AA] regardless of [AA]/[Pd^2+^] ratio, while no influence of this side reaction might be observed with the same ratios of reagents at higher total concentrations. In reactions at lower [AA], degradation of AA may slow the reaction rate later in the reaction by decreasing the amount of AA available for metal ion reduction. Given the complexities of modeling side reactions in many-component particle growth systems and the importance of certain possible side reactions, such as reducing agent degradation, in altering the nanoparticle growth environment, OCP measurements can provide valuable real-time insights into competing reaction processes.

## 3 Conclusions

The electroanalytical measurement studies presented in this work provide a wealth of real-time mechanistic information about the nanoparticle growth environment that is not observable with other approaches. Importantly, OCP measurements and their corresponding analysis are broadly generalizable and readily implementable by other researchers, providing access to information about the dynamics of metal nanoparticle growth with an elevated degree of detail, specificity, and time resolution to deepen retrospective mechanistic analysis of existing nanoparticle syntheses and accelerate predictive synthesis design. Our analysis of the data captured by in situ OCP measurements elucidates the various factors that dominate the mixed potential during different phases of metal nanoparticle growth for our synthetic systems: the reducing strength of the solution at reaction initiation, reagent diffusion (to seed particles) in the early seconds to minutes, and then the steady-state rate of metal ion reduction in the later minutes to hours. Using OCP measurements of nanoparticle growth solutions, we can observe the development of the “ideal” reducing environment for shaped particle growth is required in a time frame before the faceted shape itself can be visualized by electron microscopy. The number of samples required for point-in-time ICP analysis of kinetics could also be significantly reduced by using the slopes of the OCP measurement to identify regions where the rate changes and then selecting a smaller representative number of time points in these regions to analyze by ICP. Point-in-time ICP is time-consuming, and access to instrumentation can be prohibitive in terms of both accessibility and cost, so this would be a significant advance.

While diffusion of reactants is well known to influence reaction rates and metal nanoparticle growth, being able to measure it during nanoparticle growth in a straightforward way allows it to be understood in a more detailed and specific manner and thus used for synthetic design rather than as a post hoc rationalization of observed phenomena. Additionally, OCP measurements readily show the difference between growth onto seeds vs homogeneous nucleation based on the line shape early in the measurement. Further mechanistic determinations can be made via OCP measurements, as they can be used to distinguish between kinetic and surface passivation control of particle shape. The ability to distinguish between purely kinetic and surface passivation control of shaped particle growth holds promise for future exploration of systems controlled by a mixture of the two and/or by selective and nonselective surface binding of species like halides.

With this increased understanding, not only is OCP measurement a powerful tool for understanding mechanisms, but it can be used to more directly answer additional outstanding questions in particle synthesis. OCP measurements can show when a reaction can be modified midcourse with shape-directing reagents, versus when a particle growth pathway is “set”. Additionally, we have identified concentrations and conditions at which side reactions, such as reducing agent degradation, influence the mixed potential. Given the range of interesting synthetic questions that can be probed using OCP measurements, the technique is well positioned to continue to deepen understanding of unknowns about reaction chemistry—an especially important task for the development of novel high-index particles for specialized applications and the improvement of shaped nanoparticle synthesis for non-noble metals.

## Supplementary Material



## Notes

DOI for replication data repository, detailed experimental materials and methods, fundamental electrochemical background for OCP measurements, additional plots of ICP kinetics data, additional SEM images, additional graphs of OCP profiles, tables of parameters for and results of Nernstian kinetics calculations, and chart of nanoparticle size distribution (PDF)
