# Supplementary material for: Real-Time Electroanalytical Measurements of Dynamic Reaction Chemistry Provide Generalizable Mechanistic Considerations for Metal Nanoparticle Synthesis
Source: J Am Chem Soc. 2026 Jan 30;148(7):7280–99. doi: 10.1021/jacs.5c19321 (PMC12951458; doi:10.1021/jacs.5c19321)
Supplement: Supplementary file 1 [file ja5c19321_si_001.pdf]

Supporting Information for

Real-Time Electroanalytical Measurements of Dynamic Reaction Chemistry Provide Generalizable Mechanistic Considerations for Metal Nanoparticle Synthesis

Gabriel C. Halford, Abigail M. Sublett, Michelle L. Personick*

Department of Chemistry, University of Virginia, Charlottesville, Virginia 22904, United States

*Corresponding author email: mpersonick@virginia.edu

Replication Data

Halford, Gabriel C.; Sublett, Abigail M.; Personick, Michelle L., 2026, "Replication Data for: Real-Time Electroanalytical Measurements of Dynamic Reaction Chemistry Provide Generalizable Mechanistic Considerations for Metal Nanoparticle Synthesis," https://doi.org/10.18130/V3/2T1ILZ.

Experimental Methods

Materials

Cetyltrimethylammonium bromide (CTAB, BioXtra Lot No. SLCJ8356 ≥99% and BioUltra Lot No. BCCF7530 ≥99.0%), cetyltrimethylammonium chloride solution (CTAC, Lot No. STBL3571, 25 wt. % solution in water), L-ascorbic acid (AA, ACS reagent grade ≥99%), sodium borohydride ($NaBH_4$, 99.99% trace metal basis), palladium ICP standard solution (TraceCERT grade), sodium tetrachloropalladate(II) (Na_2PdCl_4, ≥99.99% trace metal basis), gold(III) chloride trihydrate (chloroauric acid, $HAuCl_4$, ≥99.9% trace metals basis), and silver nitrate ($AgNO_3$, 99.9999% trace metal basis) were purchased from MilliporeSigma. Hydrochloric acid (HCl, 1M solution) was purchased from VWR. Nitric acid solution (HNO_3, 1M solution), concentrated hydrochloric acid (HCl, ACS Plus grade), concentrated nitric acid (HNO_3, ACS Plus grade), high-purity concentrated hydrochloric acid (HCl, TraceMetal grade), high-purity concentrated nitric acid (HNO_3, TraceMetal grade), sulfuric acid solution (H_2SO_4, 0.5 M solution), acetone (ACS

grade, ≥99.5%), hydroquinone (HQ, 99%), sodium bromide (NaBr, 99.99% metals basis), sodium iodide (NaI, ≥99% molecular biology grade and 99.9% metals basis), palladium (II) chloride ($PdCl_2$, 99.9% metals basis), and saturated potassium chloride electrode storage solution (KCl, solution in water with potassium hydrogen phthalate stabilizer) were purchased from Thermo Fisher Scientific. Cetyltrimethylammonium hydrogen sulfate ($CTAHSO_4$, Lot No. 46XTG-ZG, >98.0%) and cetyltrimethylammonium chloride powder (CTAC, Lot No. CN6VE-OK, >95.0%) were purchased from Tokyo Chemical Industries. Alumina powder (0.05 µm and 0.3 µm MicroPolish) was purchased from Buehler. Ethanol (190 proof) was purchased from Decon Laboratories. Bis(p-sulfonatophenyl)phenylphosphine dihydrate dipotassium salt (BSPP, ≥97%) was purchased from Strem Chemicals. Saturated potassium sulfate electrode storage solution (K_2SO_4, in water) was purchased from Koslow Scientific Co.

All chemicals were used without further purification (with the exception of oven drying of CTAB as described below) and all solutions were prepared with ultrapure deionized (DI) water (18.2 MΩ resistivity, MilliQ IQ 7000). All bis(p-sulfonatophenyl)phenylphosphine dihydrate dipotassium salt (BSPP), sodium iodide, and sodium bromide solutions were prepared fresh daily. All ascorbic acid, hydroquinone, sodium borohydride, and silver nitrate solutions, as well as diluted seed solutions, were prepared immediately before use and re-made fresh on the half-hour as needed. Acetone was not added to surfactant solutions until ~10 minutes before synthesis/measurement and solutions were kept capped, to avoid evaporation.

Sodium tetrachloropalladate(II) (Na_2PdCl_4, 50 mM solution) was prepared by combining 0.294 g Na_2PdCl_4 and 20 mL of ultrapure DI water. The solution was capped and stirred for 3 hours until fully dissolved. As we previously reported, Pd CC and THH syntheses are highly sensitive to the concentration of Pd^{2+} in the growth solution;[1] for that reason, all the nanoparticle synthesis

reactions were conducted by diluting a more stable 50 mM stock solution of Na_2PdCl_4 to 10 mM concentration. This 10 mM solution was then used the same day. When a new stock solution (50 mM) was created, a tight gradient (200-300 μL of 10 mM Na_2PdCl_4 in the growth solutions for Pd THH in CTAB) was conducted to establish a consistent, high-quality THH product due to apparent solubility issues between prepared Pd stock solutions.

Tetrachloropalladic acid (H_2PdCl_4, 100 mM solution) was prepared by combining 0.354 g $PdCl_2$ and 20 mL of 0.2 M HCl in a 30 mL glass scintillation vial. The solution was capped and stirred for 3 hours until the solid fully dissolved and the solution became a clear dark orange. This solution did not suffer from the solubility issues that the sodium salt did, so calibration was not required.

For use in nanoparticle synthesis, AA and HQ stock solutions were prepared in ultrapure DI water without additional modification before addition to nanoparticle growth solutions. Acidic solutions of AA and HQ were prepared for OCP measurement of reducing agent degradation (see below) by making a dilute nitric acid stock of 3.2 mL 1 M HNO_3 in 100 mL ultrapure DI water. The dilute nitric acid stock had a pH of 1.5, similar to the pH of acidic Au and Pd nanoparticle growth solutions. 10 mL AA and HQ solutions of various concentrations from 0.1 mM to 1 M were then prepared using the dilute nitric acid stock solution as the solvent. These solutions were either measured right away or stored in a dark drawer for one week before measurement. 1 M HQ required sonication for 30+ minutes to fully dissolve and was measured or stored in a dark drawer as soon as no more solid HQ was observed.

Synthesis Procedures

Synthesis of Pd Nanocube Seeds

22 nm nanocube seeds were synthesized according to a previously reported method.[2] 10 mL of 12.5 mM BioXtra CTAB, 500 μL of 10 mM H_2PdCl_4, and a stir bar (3x10 mm) were added into a tall 30 mL glass scintillation vial. The vial was then placed in 95 °C silicone oil bath with 600 RPM stirring and allowed to equilibrate for ten minutes. 80 μL of 100 mM ascorbic acid was then injected and the solution was stirred in the oil bath for 20 minutes. After that, it was transferred to a 30 °C water bath and the seeds were allowed to sit for at least two hours to finish reacting. They maintained their quality for up to 24 hours after synthesis before developing rounded corners and/or aggregating. 10-fold diluted solutions of seeds in 12.5 mM BioUltra Lot B and BioXtra CTAB lines only maintained quality for about 15 minutes at room temperature before beginning to aggregate, so all seed dilutions were prepared immediately before use. Seeds were diluted in the same CTAB source that was used for subsequent shaped Pd nanoparticle synthesis.

Synthesis of Small Pseudospherical Pd Seeds

<10 nm Pd seeds were synthesized according to our previously reported method.[3] 10 mL of 12.5 mM CTAB and 250 μL of 10 mM Na_2PdCl_4 were added to a 20 mL scintillation vial and stirred to vortex. Once the solution was thoroughly mixed, 600 μL of freshly prepared 10 mM $NaBH_4$ was rapidly injected under continued vortex stirring, inducing an instantaneous darkening of the solution from beige to dark brown. The seed solution was allowed to stir for an additional minute to ensure uniformity in the dispersion of the reducing agent. The stirring was then halted and the seed solution was aged on the benchtop for two hours to ensure degradation of excess $NaBH_4$. Seeds were used within 24 hours of synthesis.

Synthesis of Au Seeds

~7 nm Au seeds were synthesized according to a previously reported method.[4] 10 mL of 100 mM MilliporeSigma CTAC and 250 µL of 10 mM $HAuCl_4$ were added to a 20 mL glass scintillation vial and stirred to vortex. Once the solution was thoroughly mixed, 600 µL of freshly prepared 10 mM $NaBH_4$ was rapidly injected under continued vortex stirring, inducing an instantaneous color change of the solution from yellow to red. The seed solution was allowed to stir for an additional minute to ensure uniformity in the dispersion of the reducing agent. The stirring was then halted and the seed solution was aged on the benchtop for two hours to ensure degradation of excess $NaBH_4$. Seeds were used within 24 hours of synthesis.

Colloidal Synthesis of Pd CC Nanoparticles in As-Received BioUltra Lot B CTAB

Pd CC nanoparticles were synthesized according to our previously-reported method.[1] A growth solution was prepared by adding 10 mL of 50 mM CTAB (as-received BioUltra Lot B), 200 µL of 10 mM Na_2PdCl_4 (subject to equilibration as described in previous section), and 100 µL of 0.1 M HNO_3 to a 20 mL glass scintillation vial (for unmeasured synthesis and/or ICP sample preparation) or a five-neck flask (for OCP measurement). The vial was placed in a 40 °C water bath and allowed to come to temperature for at least 10 minutes. To this warm solution, 100 µL of fresh 100 mM ascorbic acid was added, the solution swirled, and 100 µL of 10x diluted nanocube seeds (seeds diluted in 12.5 mM BioUltra Lot B CTAB) were injected, and the solution was swirled again. The vial was then put back into the 40 °C water bath for at least 15 hours.

Colloidal Synthesis of Pd CC Nanoparticles in As-Received BioXtra CTAB

Pd CC nanoparticles were synthesized according to our previously-reported method.[1] A growth solution was prepared by adding 10 mL of 50 mM CTAB (as-received BioXtra), 200 µL of 10 mM Na_2PdCl_4 (subject to equilibration as described in previous section), and 100 µL of 0.1 M

HNO_3 to a 20 mL scintillation vial (for unmeasured synthesis and/or ICP sample preparation) or a five-neck flask (for OCP measurement). The vial was placed in a 40 °C water bath and allowed to come to temperature for at least 10 minutes. To this warm solution, 100 µL of fresh 100 mM ascorbic acid was added, the solution swirled, and 100 µL of 10x diluted nanocube seeds (seeds diluted in 12.5 mM BioXtra CTAB) were injected, and the solution was swirled again. The vial was then put back into the 40 °C water bath for at least 15 hours.

Colloidal Synthesis of Pd THH Nanoparticles in As-Received BioUltra Lot B CTAB

Pd THH nanoparticles were synthesized according to our previously-reported method.[1] A growth solution was prepared by adding 10 mL of 50 mM CTAB (as-received BioUltra Lot B), 200 µL of 10 mM Na_2PdCl_4 (subject to equilibration as described in previous section), 100 µL of 0.1 M HNO_3, 50 µL of 0.1 mM freshly-prepared NaI (Thermo Fisher molecular biology grade) and 50 µL neat acetone to a 20 mL glass scintillation vial (for unmeasured synthesis and/or ICP sample preparation) or a five-neck flask (for OCP measurement). The vial was placed in a 40 °C water bath and allowed to come to temperature for at least 10 minutes. To this warm solution, 100 µL of fresh 100 mM ascorbic acid was added, the solution swirled, and 100 µL of 10x diluted nanocube seeds (seeds diluted in 12.5 mM BioUltra Lot B CTAB) were injected, and the solution was swirled again. The vial was then put back into the 40 °C water bath for at least 15 hours.

Drying Procedure for CTAB (BioUltra Lot B and BioXtra)

Commercial CTAB powder was placed into a tall 30 mL glass scintillation vial. The vial was placed uncapped in an oven at 80 °C for at least 24 hours. CTAB was dried in ~10 g batches. The CTAB was stirred with a new Pasteur pipette after ~12 hours to ensure appropriate drying of powder at the bottom of the vial. Vials of dried CTAB were then stored capped on the benchtop without further purification.

Colloidal Synthesis of Pd CC, THH, and TC Nanoparticles in Dried BioUltra Lot B CTAB

Pd CC, THH, and TC nanoparticles were synthesized according to our previously-reported method.[1] A growth solution was prepared by adding 10 mL of 50 mM CTAB (oven-dried BioUltra Lot B), 10 mM Na_2PdCl_4 (125 µL for CC, 200 µL for THH, and 400 µL for TC; subject to equilibration as described in previous section), 100 µL of 0.1 M HNO_3, 70 µL of 0.1 mM freshly-prepared NaI (Thermo Fisher molecular biology grade) and 190 µL neat acetone to a 20 mL glass scintillation vial (for unmeasured synthesis and/or ICP sample preparation) or a five-neck flask (for OCP measurement). The vial was placed in a 40 °C water bath and allowed to come to temperature for at least 10 minutes. To this warm solution, 100 µL of fresh 100 mM ascorbic acid was added, the solution swirled, and 100 µL of 10x diluted nanocube seeds (seeds diluted in 12.5 mM BioUltra Lot B CTAB) were injected, and the solution was swirled again. The vial was then put back into the 40 °C water bath for at least 15 hours.

Colloidal Synthesis of Pd THH Nanoparticles in Dried BioXtra CTAB

Pd THH nanoparticles were synthesized according to our previously-reported method.[1] A growth solution was prepared by adding 10 mL of 50 mM CTAB (oven-dried BioXtra), 200 µL of 10 mM Na_2PdCl_4 (subject to equilibration as described in previous section), 100 µL of 0.1 M HNO_3, 60 µL of 0.1 mM freshly-prepared NaI (Thermo Fisher molecular biology grade), and 230 µL neat acetone to a 20 mL glass scintillation vial (for unmeasured synthesis and/or ICP sample preparation) or a five-neck flask (for OCP measurement). The vial was placed in a 40 °C water bath and allowed to come to temperature for at least 10 minutes. To this warm solution, 100 µL of fresh 100 mM ascorbic acid was added, the solution swirled, and 100 µL of 10x diluted nanocube seeds (seeds diluted in 12.5 mM BioXtra CTAB) were injected, and the solution was swirled again. The vial was then put back into the 40 °C water bath for at least 15 hours.

Colloidal Synthesis of Pd CC and THH Nanoparticles in High-Concentration, As-Received BioUltra Lot B CTAB

A growth solution was prepared by adding 10 mL of 150 mM CTAB (as-received BioUltra Lot B), x µL of 10 mM Na_2PdCl_4 (x = 125 µL for CX and 150 to 500 µL for THH), 100 µL of 0.1 M HNO_3, and 50 µL of 0.1 mM freshly-prepared NaI (Thermo Fisher molecular biology grade) to a 20 mL glass scintillation vial (for unmeasured synthesis and/or ICP sample preparation) or a five-neck flask (for OCP measurement). The vial was placed in a 40 °C water bath and allowed to come to temperature for at least 10 minutes. To this warm solution, 100 µL of fresh 100 mM ascorbic acid was added, the solution swirled, and 100 µL of 10x diluted nanocube seeds (seeds diluted in 12.5 mM as-received BioUltra Lot B CTAB) were injected, and the solution was swirled again. The vial was then put back into the 40 °C water bath for at least 15 hours.

Colloidal Synthesis of Homogeneously Nucleated Pd Cubes and Octahedra

Pd cubes and octahedra were synthesized via modification of a previously reported method.[5,6] A solution was prepared by adding 10 ml of 15 mM CTAC (TCI powder), 1.050 mL of 10 mM H_2PdCl_4, and 75 µL of 1 mM NaBr (to form cubes) or 7.5 µL of 1 mM NaI (Thermo Fisher metals basis; to form octahedra) to a 20 mL scintillation vial, which was swirled and placed in a 35 °C water bath for 30 minutes to allow partial ligand exchange and temperature equilibration. To this warm solution, 1 mL of 100 mM ascorbic acid was added, the solution swirled, and the vial was then put back into the 35 °C water bath for at least 30 minutes.

Colloidal Synthesis of Pd Nanoparticles in $CTAHSO_4$ with Ascorbic Acid

Pd nanoparticles were synthesized via modification of our previously reported method.[6] 10 mL of 100 mM $CTAHSO_4$ and 500 µL of 10 mM H_2PdCl_4 were added to a 20 mL scintillation vial and swirled, then placed in a 40°C water bath for 10 minutes. 100 µL of 100 mM ascorbic acid and 100 µL of 1000x diluted small Pd pseudospherical seeds (in 12.5 mM $CTAHSO_4$) were added

and growth solution was swirled again. The reaction was left undisturbed in the 40°C water bath for at least 30 minutes.

Colloidal Synthesis of Pd Nanoparticles in $CTAHSO_4$ with Hydroquinone

Pd nanoparticles were synthesized via modification of our previously reported method.[6] 10 mL of 100 mM $CTAHSO_4$ and 500 µL of 10 mM H_2PdCl_4 were added to a scintillation vial and swirled, then placed in a 40°C water bath for 10 minutes. 100 µL of 100 mM hydroquinone and 100 µL of 1000x diluted small Pd pseudospherical seeds (in 12.5 mM $CTAHSO_4$) were added and growth solution was swirled again. The reaction was left undisturbed in the 40°C water bath for at least 30 minutes.

Colloidal Synthesis of Au-only Nanoparticles

Au nanoparticles were synthesized via modification of a previously reported method.[4,7] A growth solution was prepared by adding 10 mL of 100 mM room-temperature CTAC (Sigma 25 wt. % solution), 500 µL of 10 mM $HAuCl_4$, and 200 µL of 1 M HCl to a 20 mL scintillation vial and swirling. 100 µL of 100 mM fresh ascorbic acid was added and the vial was swirled again. 100 µL of 1000x diluted Au seed particles (in 100 mM Sigma 25 wt. % CTAC) were added to the growth solution. The reaction was swirled immediately after the addition of the seeds and then left undisturbed on the bench top for at least 30 minutes.

Colloidal Synthesis of Shape-Controlled (Ag)Au Nanoparticles

*(*Ag)Au nanoparticles were synthesized using a previously reported method.[4,7] A growth solution was prepared by adding 10 mL of 100 mM room-temperature CTAC (Sigma 25 wt. % solution), 500 µL of 10 mM $HAuCl_4$, 200 µL of 1 M HCl, and x µL of 10 mM freshly-prepared $AgNO_3$ (x = 1 µL for octahedra, 10 µL for rhombic dodecahedra, 40 µL for truncated ditetragonal prisms, and 100 µL for concave cubes) to a 20 mL glass scintillation vial and swirling. 100 µL of

100 mM fresh ascorbic acid was added and the vial was swirled again. 100 μL of 1000x diluted Au seed particles (in 100 mM Sigma 25 wt. % CTAC) were added to the growth solution. The reaction was swirled immediately after the addition of the seeds and then left undisturbed on the bench top for ~180 minutes.

Other Methods

Electrode Preparation and Reference Electrode Benchmarking

Glassy carbon working electrodes (GCEs; 5 mm OD x 4 mm thick disk insert, Pine Research) were prepared by polishing on MasterTex Buehler polishing pad with a slurry of 0.3 μM alumina polishing powder (Buehler MicroPolish Alumina), rinsed with ultrapure DI water, polished again with 0.05 μm alumina polishing powder (Buehler MicroPolish Alumina) on a different polishing pad and rinsed again. Polished electrodes were then sonicated in a glass scintillation vial in ultrapure DI water for 15 minutes three times (with water changed after each sonication step to remove alumina), sonicated in ethanol for 15 minutes, and rinsed with ultrapure DI water before being inserted into a Teflon electrode tip holder (Pine Research). Once assembled, the electrode in the Teflon tip holder was rinsed and dried again.

Reference electrodes were stored at room temperature, away from direct sunlight. Ag/AgCl electrodes were kept in saturated potassium chloride storage solution (KCl, solution in water with potassium hydrogen phthalate, Thermo Fisher Scientific Accumet Electrode Storage Solution), and Hg/Hg_2SO_4 reference electrodes were kept in saturated potassium sulfate storage solution (K_2SO_4, in water, Koslow Scientific Co.) at all times when not in use. All reference electrodes were labeled and each electrode was stored in a corresponding dedicated vial of storage solution. Saturated Hg/Hg_2SO_4 reference electrodes were prone to the formation of salt crystals near the porous frit, preventing appropriate ion exchange. These crystals were periodically eliminated by

placing a drop of hot ultrapure DI water in the K_2SO_4 storage solution container and putting the reference electrode (in the storage solution) in a 30°C water bath for 30-60 minutes.

Open-circuit potential (OCP) measurements were taken using a polished GCE as the working electrode and a Ag/AgCl (saturated KCl electrolyte) or a Hg/Hg_2SO_4 (saturated K_2SO_4 electrolyte) reference electrode (both from Koslow Scientific Co.). Ag/AgCl reference electrodes were used for all measurements of Pd particle synthesis. Hg/Hg_2SO_4 reference electrodes were used for all measurements of Au particle synthesis, to avoid leakage of Ag^+ ions,[8] which affect Au particle synthesis at very dilute concentrations.[7] The reference electrode was isolated from the reaction solution by a bridge tube with glass frit containing 100 mM sulfuric acid to stop leakage of ions and to prevent damage to the reference electrode.

Standard Pd THH growth conditions were measured with both types of reference electrodes, and the measured potentials were found to be in good agreement (accounting for the conversion between reference electrode types). All reference electrodes were benchmarked (in bridge tubes, in saturated KCl electrolyte solution) against a "master" Ag/AgCl reference electrode, with a variance between all Ag/AgCl reference electrodes tested of ± 0.007 V and between all Hg/Hg_2SO_4 reference electrodes tested of ± 0.009 V across an 1800-second benchmarking measurement. Measured solution potentials were normalized to a standard hydrogen electrode (SHE) scale to streamline comparison between measurements by adding 0.197 V to all Ag/AgCl electrode measurements and 0.640 V to all Hg/Hg_2SO_4 electrode measurements.

Open-Circuit Potential Measurements of Colloidal Particle Synthesis

An electrochemical cell was prepared in a 4- or 5-neck heart-shaped flask (Gamry Instruments Dr. Bob's Cell) using a working electrode (polished GCE) and reference electrode (Ag/AgCl or Hg/Hg_2SO_4; as previously described), as well as a miniature stir bar (3x10 mm). A counter

electrode was not necessary—as no current was being passed—and therefore was not used (except for in our previously-reported Pd CC and THH OCP data, where a Pt wire counter electrode was used).[1] Our early tests of OCP measurement of Au particle synthesis with Pt wire, foil, and mesh counter electrodes showed Au plating on the Pt counter electrode (data not reported in this work), so we omitted the counter electrode in all cases in this work to avoid influencing the concentration of metal ions in solution. The electrodes were immersed in a particle growth solution containing all reagents except for the chemical reducing agent and seed particles. The reference electrode was isolated from the reaction solution by a bridge tube with glass frit containing 100 mM sulfuric acid. The necks of the flask that were not in use were capped. The electrochemical cell was clamped above a stir plate with a 40°C water bath (except in the case of homogeneously nucleated Pd cubes and octahedra, where a 35°C water bath was used), and electrode leads were connected to the potentiostat (Gamry Instruments 1010B or 1010E). The growth solution was stirred at 200 RPM for ten seconds. After that time, an OCP measurement was started using the Gamry Framework software interface. The electrochemical cell was uncapped, and reducing agent and seeds were rapidly injected into the growth solution. For most seeded reactions, including simple Pd growth solutions and Au particle growth, the reducing agent was added immediately before the seeds. However, for Pd CC and THH in CTAB, the seeds were injected before the reducing agent, and this is important for product quality. Stirring was continued for 10 seconds after injection of seeds and reducing agent, then turned off. OCP data was analyzed using Gamry Echem Analyst software and MATLAB. Any data from >60 seconds prior to the introduction of both seeds and reducing agent was omitted from plots of OCP traces of colloidal reactions.

Open-Circuit Potential Measurements of Reducing Agent Degradation

Measurements were taken identically to the procedure described above for measurement of particle synthesis, except that the acidic solution of reducing agent (either fresh or aged) was present at the start of the measurement and was not stirred. An Ag/AgCl reference electrode was used.

Open-Circuit Potential Measurements Under Argon Atmosphere

Electrochemical glassware was prepared as described above, with the addition of a fritted glass bubbler connected to benchtop argon (Ar) gas supply. The bubbler was positioned so that it was just above but not submerged in the growth solution, as sparging of surfactant solutions causes significant bubbling. Rather than capping unused necks of the flask with stoppers as usual, all openings to the electrochemical flask were covered securely with Parafilm. A needle was added to one of the necks for pressure release. Ar flow was turned on ≥15 minutes before the initiation of measurement and kept on throughout the measurement. Rather than adding reagents with a micropipette, as usual, reducing agent and diluted seed solution were injected into the growth solution rapidly through the Parafilm covering using a syringe and PTFE needle. Measurements were otherwise conducted identically to the procedure described above for measurement of particle synthesis.

Considerations and Cleaning for Glassware and Electrochemical Apparatus

Large Pd cube seeds and Pd salt stock solutions were prepared in 30 mL glass vials (Fisherbrand™ Class A Clear Glass Threaded Vials with Attached Caps, PE Poly-Seal™ Cone Liner). Small Pd seeds, Au seeds, all other stock solutions, and all growth solutions (except for those prepared in a heart-shaped electrochemical flask for OCP measurement) were prepared in 20 mL glass scintillation vials (DWK Life Sciences 986560; Wheaton™ Glass 20 mL Scintillation

Vials: Polypropylene Caps, Foamed Polyethylene Liner). Pd THH and (Ag)Au syntheses were sensitive to the use of other lines of glass scintillation vials or plastic vials, and the expected shape development did not occur when other vials were used.

Electrochemistry glassware, including 4- or 5-neck heart-shaped flasks, bridge tubes for reference electrodes, miniature stir bars (3x10 mm), glass stoppers, and Teflon holders for glassy carbon disk electrode inserts were cleaned with aqua regia (1:3 ratio of concentrated nitric acid: concentrated hydrochloric acid; **caution: strong acid**) between uses. Porous glass frits on bridge tubes were replaced every ~8 weeks or whenever they became permanently discolored. Bridge tubes were used for only one type of metal and reference electrode between cleaning with aqua regia and/or frit replacement, to avoid contamination. Electrochemistry flasks that were used for syntheses that contain chloride only; chloride and bromide; or chloride, bromide, and iodide were kept separate, as adsorbed halides were challenging to remove from the glassware. Glassware that had contained bromide or iodide was washed in 50°C ultrapure DI water, which cleaned off halide residues more successfully than room-temperature water.

Characterization of Nanoparticles by Electron Microscopy

To prepare colloidal samples for characterization via scanning electron microscopy (SEM), the completed reaction vial was vortexed, sonicated briefly (around 30 seconds), and vortexed again. From this dark, cloudy solution, 1 mL was removed and transferred to a microcentrifuge tube. The solution was then centrifuged (VWR Micro Star 21 or Eppendorf 5430) at 6000 rpm for four minutes to spin down a pellet. The supernatant was removed with a Pasteur pipette, and the microcentrifuge tube refilled to 1 mL with ultrapure DI water, vortexed, sonicated, vortexed, and then centrifuged again at 6000 rpm for four minutes. The supernatant was removed again, and a minimal amount of ultrapure DI water (200-400 μL) was added, and the tube was sonicated and

vortexed to disperse the particles. 1 μL of this solution was then dropcast onto a prepared silicon wafer and allowed to dry for at least 45 minutes in a desiccator before characterization by SEM (FEI Quanta 650 FE-SEM). For point-in-time SEM imaging, this procedure was followed identically at each timepoint of interest. A new reaction vial was used for each timepoint. Au and (Ag)Au nanoparticles were imaged within 48 hours of sample preparation due to oxidative etching of particles; etching of Pd nanoparticle SEM samples was not observed. Average particle size (ImageJ) was determined using >100 particles. Shape yield was determined using >1000 particles.

Transmission electron microscopy (TEM) samples of early Pd concave cube synthesis timepoints were prepared to minimize loss of small particles during sample preparation. At the timepoint of interest, 1 mL of growth solution was removed and centrifuged immediately for 4 minutes at 6000 RPM. A pellet did not form, but particles became concentrated in the bottom portion of the microcentrifuge tube. Approximately 0.5 mL of supernatant from the top of the tube was removed, but particles were not washed further. 2 μL of sample was dropcast on a TEM grid (Formvar/carbon film, 400 mesh, Cu grid; Ted Pella #01754-F) and allowed to dry for ≥1 hour. Images were taken within 24 hours of sample preparation using a 120kV Tecnai Spirit TEM. A new reaction vial was used for each timepoint.

Brightness and contrast of electron microscopy images were normalized using the Curves adjustment tool in Adobe Photoshop.

ICP-OES Kinetics Sample Preparation and Instrumentation

Reduced Pd (pellet) samples were prepared as follows: Samples were prepared by removing a 1 mL aliquot from reaction solutions at different time points, with a separate but identical reaction solution used for each time point. The aliquots were immediately quenched by injecting 100 μL of 10 mM BSPP and then vortexed to ensure uniform dispersion. The samples were then centrifuged,

within one hour of quenching, at 14,800 rpm for 15 minutes (VWR Micro Star 21) and the supernatant was removed with care to ensure the particle pellet at the bottom of the tube was undisturbed. The particles were then washed with ultrapure DI water and the previous procedure was repeated. After washing, the particles were resuspended in 1 mL of ultrapure DI water, sonicated for 10 minutes, and carefully transferred to a 15 mL conical tube. The solution in the 15 mL tube was then digested with 2 mL of aqua regia (1:3 nitric acid to hydrochloric acid) made from concentrated trace metals grade acids (**caution: strong acid**). The particles were allowed to digest overnight and were then diluted to 5 mL total volume with 2 mL of ultrapure DI water and vortexed to mix. Samples were prepared in triplicate for each timepoint. All samples were syringe filtered prior to analysis using a filter with pore size ≤ 0.2 μm (GE Healthcare Life Sciences Whatman GD/X 25 mm syringe filter, 0.2 μm pore, PTFE membrane).

Pd^{2+} (supernatant) samples were prepared as follows: Samples were prepared by removing a 1 mL aliquot from reaction solutions at different time points, with a separate but identical reaction solution used for each time point. The aliquots were immediately quenched by injecting 100 μL of 10 mM BSPP and then vortexed to ensure uniform dispersion. The samples were then centrifuged, within one hour of quenching, at 14,800 rpm for 15 minutes (VWR Micro Star 21) and the supernatant was removed with care to ensure the particle pellet at the bottom of the tube was undisturbed. The supernatant was carefully transferred to a different microcentrifuge tube. Samples were syringe filtered twice to remove excess surfactant prior to analysis using a filter with pore size ≤ 0.2 μm (GE Healthcare Life Sciences Whatman GD/X 25 mm syringe filter, 0.2 μm pore, PTFE membrane; new filter and syringe used for each step). After filtering, samples were transferred to a 15 mL conical tube. The solution in the 15 mL tube was then digested with 2 mL of aqua regia (1:3 nitric acid to hydrochloric acid) made from concentrated trace metals grade acids

(**caution: strong acid**). The solution was allowed to digest overnight and was then diluted to 5 mL total volume with 2 mL ultrapure DI water and vortexed to mix. Samples were prepared in triplicate for each timepoint. All samples were syringe filtered again prior to analysis using a filter with pore size ≤ 0.2 µm (GE Healthcare Life Sciences Whatman GD/X 25 mm syringe filter, 0.2 µm pore, PTFE membrane). We note that, while ICP kinetics data from supernatant sample preparation method was in reasonable agreement with the typical pellet sample preparation method, we recommend the pellet sample preparation method. The supernatant sample preparation method seems to produce less precise results More importantly, the introduction of samples containing surfactants to ICP spectrometers risks damaging the instrument, requiring additional filtration steps and extra caution.

Fresh Pd ICP standards were also prepared fresh in a 40% trace metals grade aqua regia/60% DI water matrix each time new samples were prepared for analysis. Inductively coupled plasma optical emission spectroscopy (ICP-OES) measurements were carried out using a PerkinElmer Avio 200 ICP-OES. Concentrations obtained from the raw ICP intensities and calibration curve were multiplied by 5 to account for the 5-fold sample dilution factor. For point-in-time ICP measurements of Pd particle synthesis in CTAB, the data was then multiplied by a scale factor of $0.24/x$ (where x is equal to the concentration of Pd^{2+} initially added to the growth solution in mM, and 0.24 mM Pd^{2+} is the standard concentration of Pd^{2+} used to form Pd THH) to account for the different input [Pd^{2+}] values and allow for easy measurement-to-measurement comparison.

Fundamentals of Open-Circuit Potential (OCP) Measurements

A brief fundamental background on OCP measurements is given in this section to provide context for the data shown in this work for those who are less familiar with electroanalytical measurement approaches. OCP measurements are taken in an electrochemical cell containing a working electrode (WE; for example: glassy carbon (GC)) and a reference electrode (RE; for example: silver/silver chloride (Ag/AgCl) or mercury/mercurous sulfate (Hg/Hg_2SO_4)). The OCP is a measure of the potential between the WE and RE when there is no externally applied current or potential, and, therefore, no net current flow in the electrochemical cell.[9] Though OCP measurements are often conceptualized as being representative of the Nernstian potential of the redox-active species in solution, this is not strictly true under most conditions, including many of the measurement conditions presented in this work. While the OCP can match the Nernst potential of a particular redox couple if it is "poised" to that redox couple (no other significant electrochemical events are occurring in solution or at the working electrode surface), the OCP more generally represents the "mixed" solution potential of all electroactive species.[9,10]

The "mixed" potential sensed at the working electrode during an OCP measurement is the sum of all anodic and cathodic currents (from each anodic and cathodic half reaction) taking place at or near the working electrode. Because the net current is zero, the sums of the anodic and cathodic currents must be equal in magnitude but opposite in sign.[9] However, as long as their absolute values remain equal, the sums of the anodic and cathodic currents can and do change over time under non-equilibrium conditions, changing the zero current point (the OCP).

Under metal nanoparticle growth conditions, such as the ones studied in this work, the OCP is a mixed potential with contributions from the sets of half reactions associated with metal ion reduction and reducing agent oxidation.[11] Additionally, because both the synthesis and the OCP measurement are conducted in air under ambient conditions, the dissolved oxygen concentration

and, in sufficiently reducing solutions, oxygen reduction both contribute to the OCP.[12–15] All electroactive species, such as halide salts, acid/base additives, ionic surfactants, and some other organic capping agents, also contribute to the anodic or cathodic currents that make up the OCP. The OCP is more generally affected by solute concentrations, mass transport limitations, solution resistance, the geometry of the WE and RE setup, and local or bulk pH changes.[9,16,17]

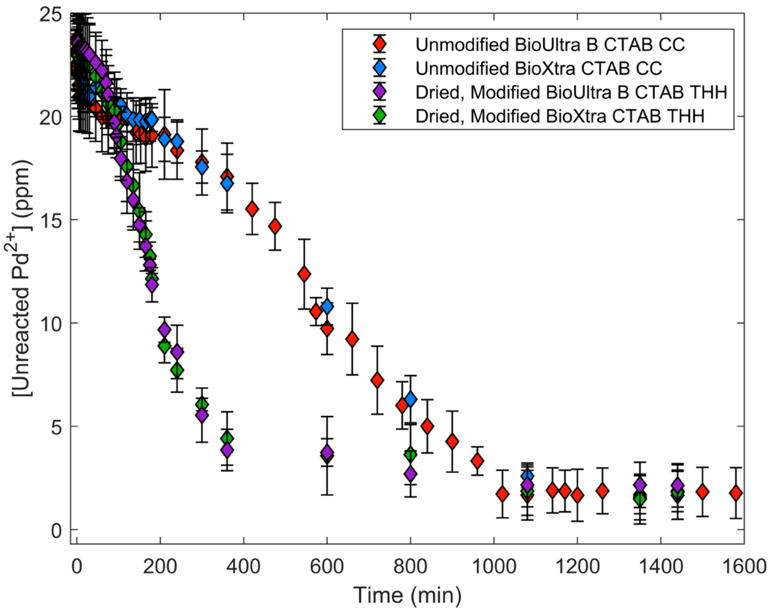

Figure S1. Inductively coupled plasma (ICP) kinetics data showing the amount of unreduced Pd^{2+} (ppm) in solution (unincorporated into growing nanoparticles) over time for reactions that yield Pd CC (red, blue) or Pd THH (purple, green).

Table S1. Extent of Pd^{2+} Ion Reduction During Pd CC and Pd THH Growth in BioUltra B and BioXtra CTAB Sources.

Sample	% of Pd^{2+} Reacted
BioUltra B CC	91 ± 1
BioXtra CC	94 ± 2
BioUltra B THH	89 ± 3
BioXtra THH	91 ± 2

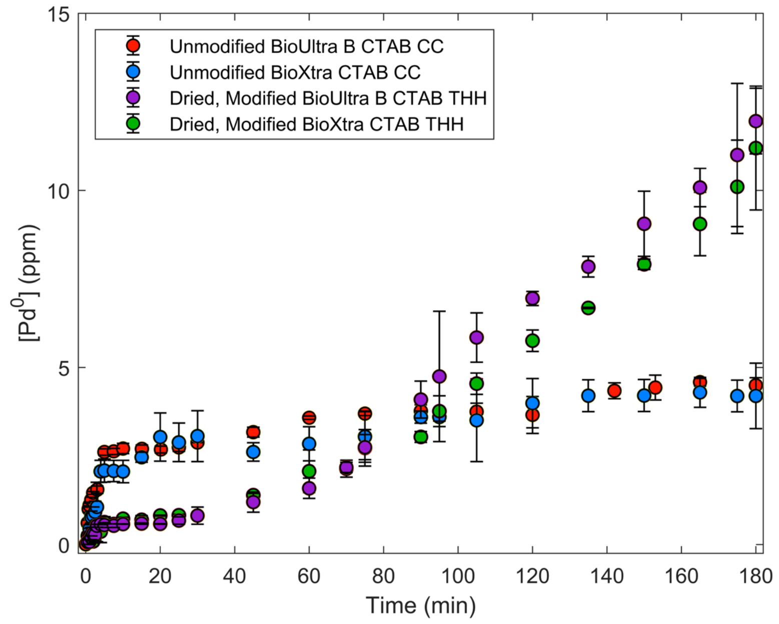

Figure S2. Zoomed-in plot of the first 180 minutes of inductively coupled plasma (ICP) kinetics data showing the amount of reduced Pd (ppm) incorporated into nanoparticles over time (representative of the rate of metal ion reduction) for reactions that yield Pd CC (red, blue) or Pd THH (purple, green).

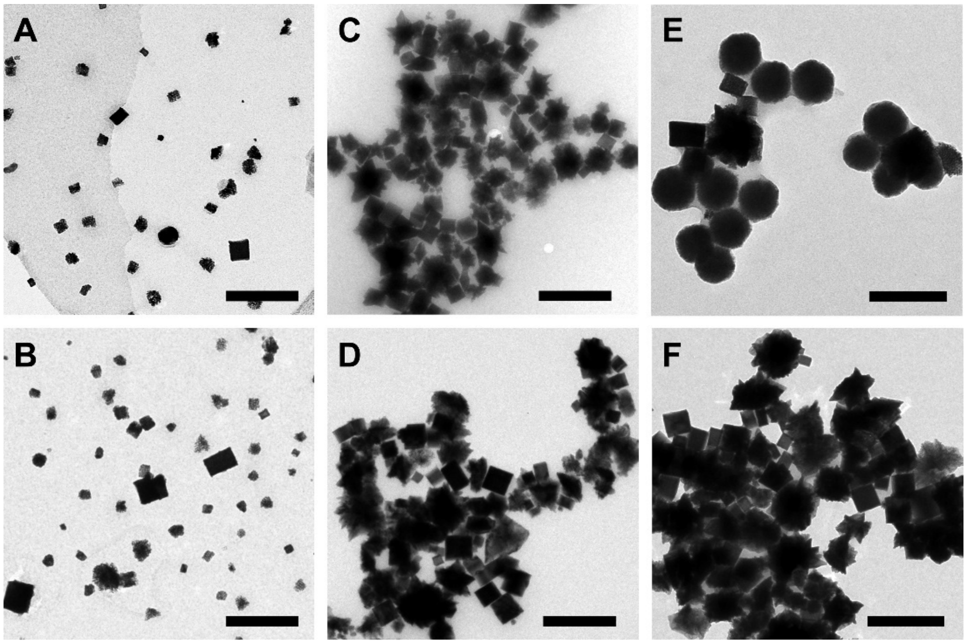

Figure S3. Point-in-time TEM images of Pd CC growth at early timepoints in as-received BioUltra B CTAB at (A, B) 10 min, (C, D) 30 min, (E, F) 60 min. Scale bars: 100 nm.

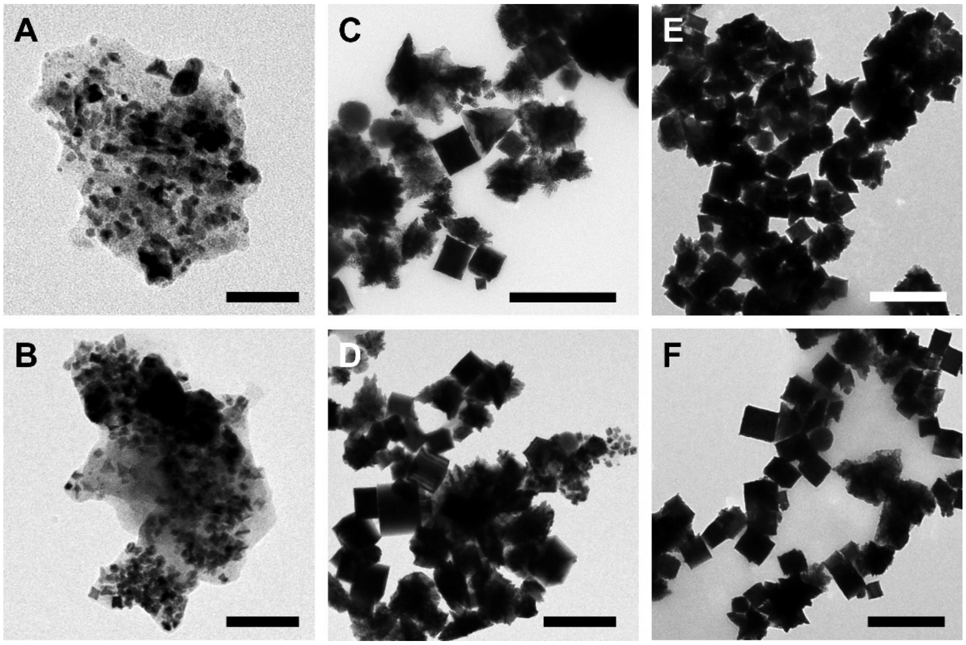

Figure S4. Point-in-time TEM images of Pd CC growth at early timepoints in as-received BioXtra CTAB at (A, B) 10 min, (C, D) 30 min, (E, F) 60 min. Scale bars: 100 nm.

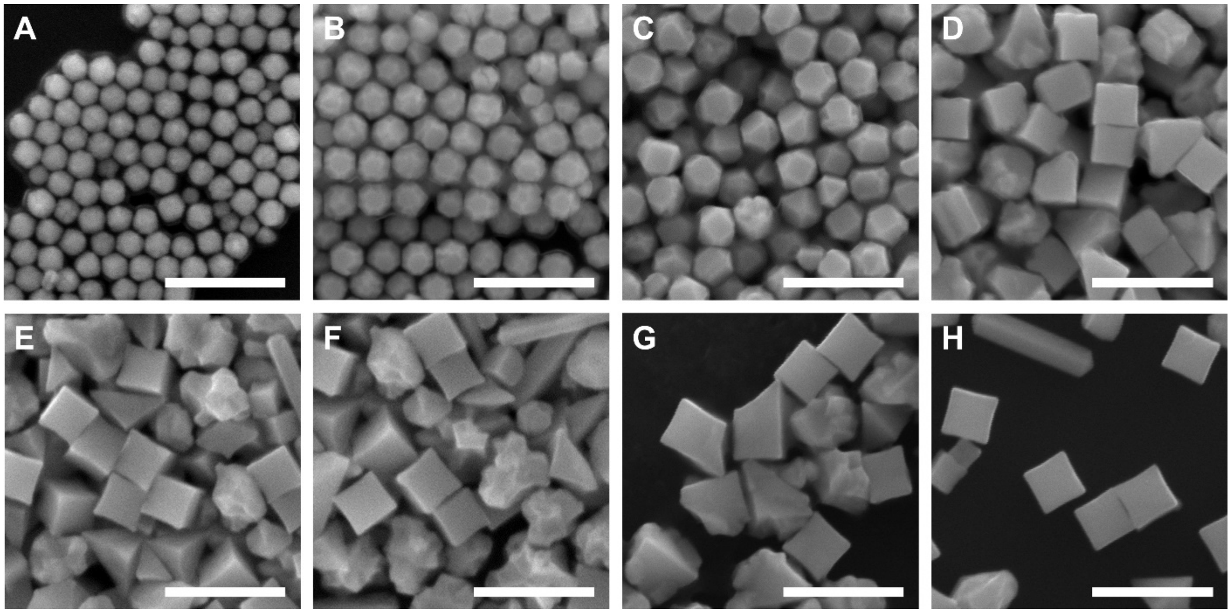

Figure S5. Point-in-time SEM images of Pd CC growth in as-received BioUltra Lot B CTAB at (A) 60 min, (B) 120 min, (C) 180 min, (D) 240 min, (E) 360 min, (F) 600 min, (G) 1260 min, and (H) 1440 min. Scale bars: 500 nm.

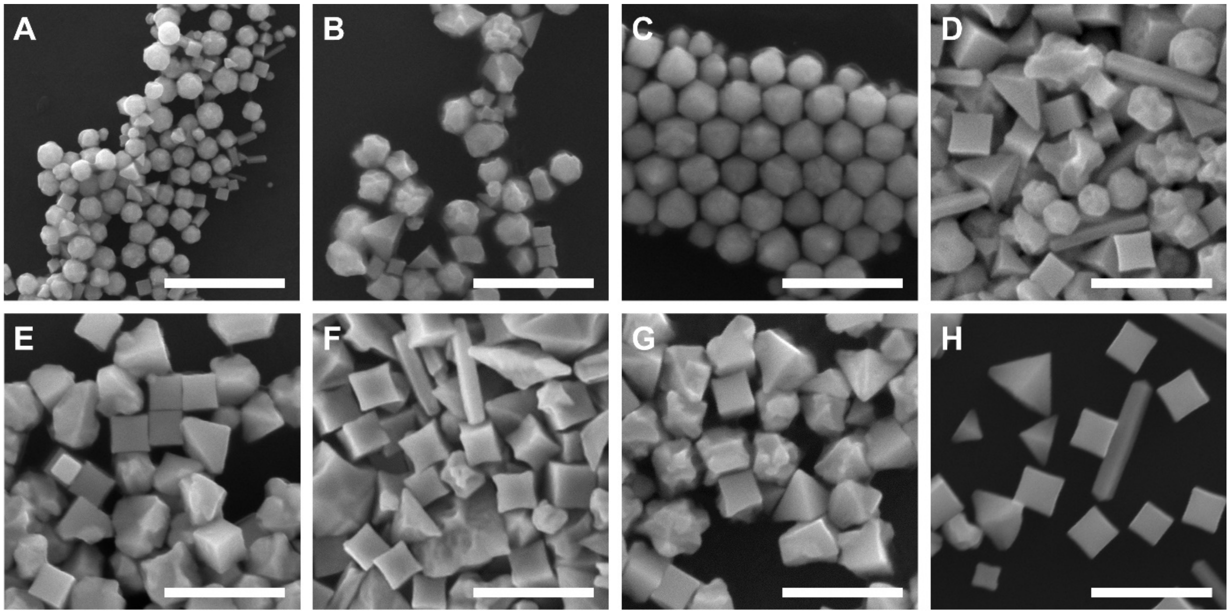

Figure S6. Point-in-time SEM images of Pd CC growth in as-received BioXtra CTAB at (A) 60 min, (B) 120 min, (C) 180 min, (D) 240 min, (E) 360 min, (F) 600 min, (G) 1260 min, and (H) 1500 min. Scale bars: 500 nm.

Table S2. Shape Yield for Pd CC Grown in BioUltra B and BioXtra CTAB.

**BioUltra B CTAB**			**BioXtra CTAB**		
**Shape**	**Count (of 1293)**	**%**	**Shape**	**Count (of 1176)**	**%**
concave cube	1077	83.2	concave cube	873	74.3
concave right bipyramid (single twinned)	102	7.9	concave right bipyramid (single twinned)	117	10.0
rod (5-fold twinned)	86	6.7	rod (5-fold twinned)	98	8.3
decahedron (5-fold twinned)	11	0.9	decahedron (5-fold twinned)	39	3.3
20-fold twinned	15	1.2	20-fold twinned	26	2.1
not faceted	2	0.1	not faceted	23	2.0

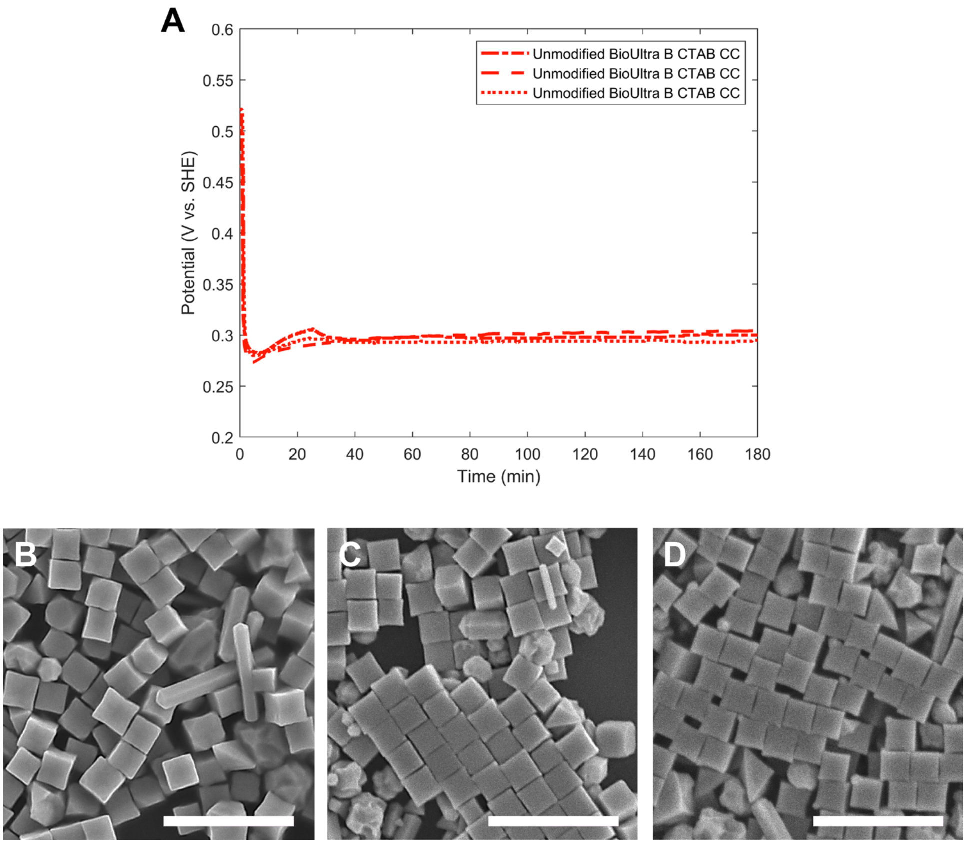

Figure S7. (A) Triplicate OCP measurements of Pd CC growth in BioUltra B CTAB. (B-D) SEM images of the Pd CC products from each growth solution. Scale bars: 500 nm. Red dot-dash trace in panel A adapted with permission from ref. 33. Available under a Creative Commons Attribution-NonCommercial 3.0 Unported License. Copyright 2024 Halford and McDarby *et al.* Image of resulting growth solution is previously unpublished.

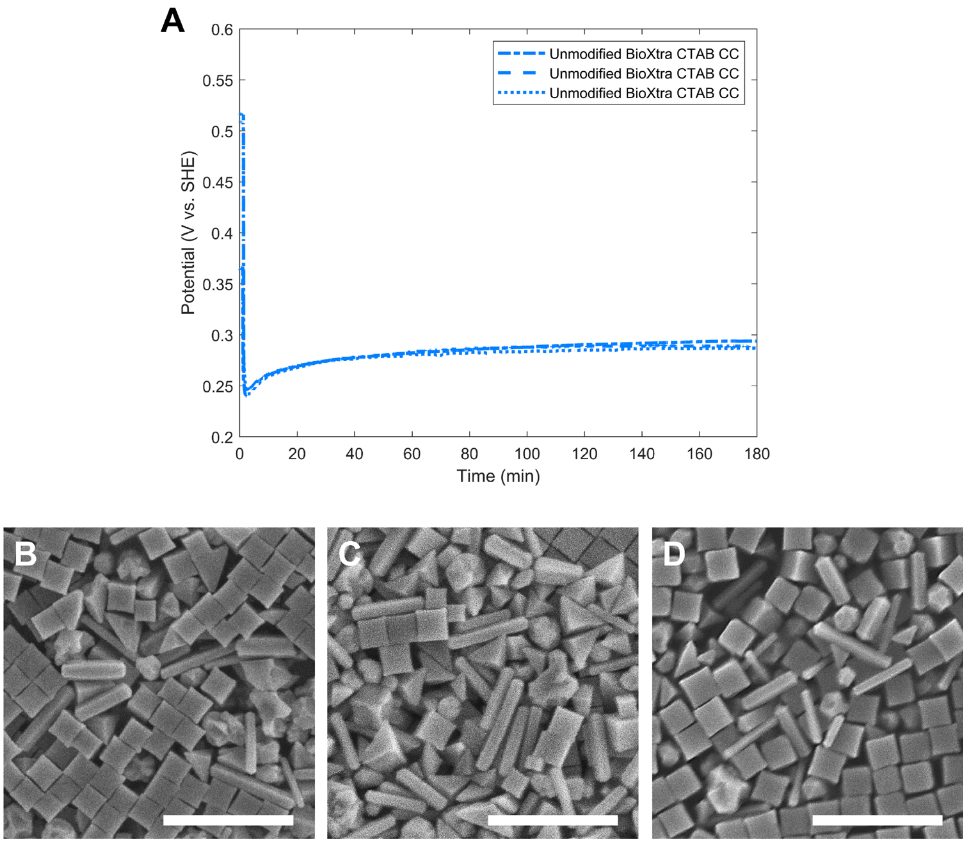

Figure S8. (A) Triplicate OCP measurements of Pd CC growth in BioXtra CTAB. (B-D) SEM images of the Pd CC products from each growth solution. Scale bars: 500 nm. Blue dot-dash trace in panel A adapted with permission from ref. 33. Available under a Creative Commons Attribution-NonCommercial 3.0 Unported License. Copyright 2024 Halford and McDarby *et al.* Image of resulting growth solution is previously unpublished.

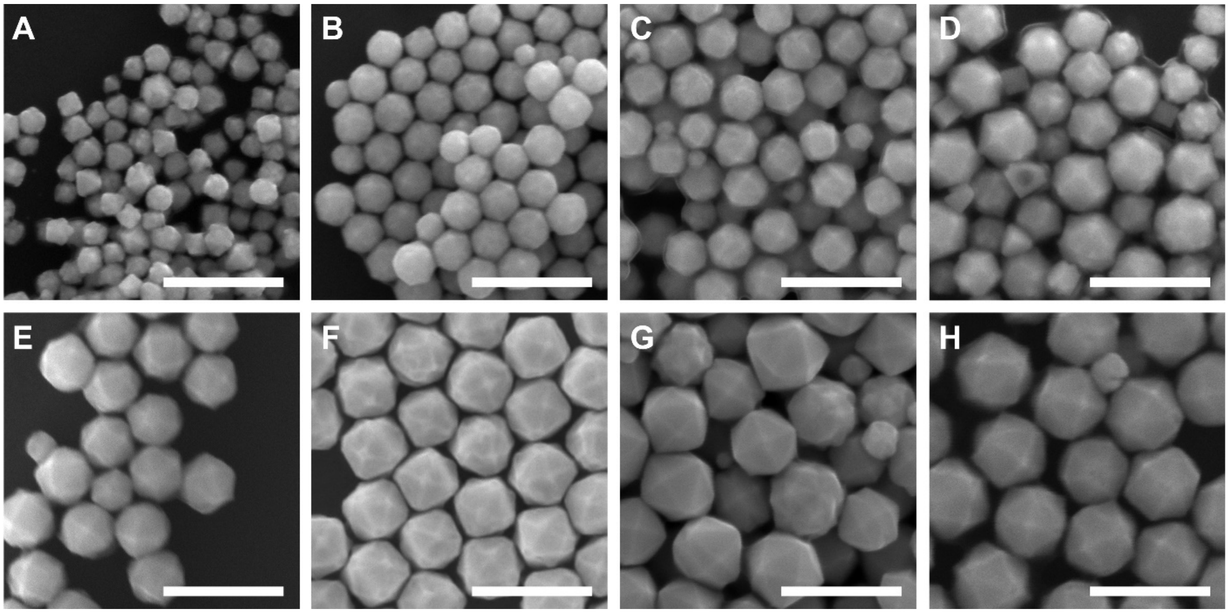

Figure S9. Point-in-time SEM images of Pd THH growth in dried BioUltra Lot B CTAB with acetone and iodide additives at (A) 60 min, (B) 120 min, (C) 180 min, (D) 240 min, (E) 360 min, (F) 600 min, (G) 1260 min, and (H) 1500 min. Scale bars: 500 nm.

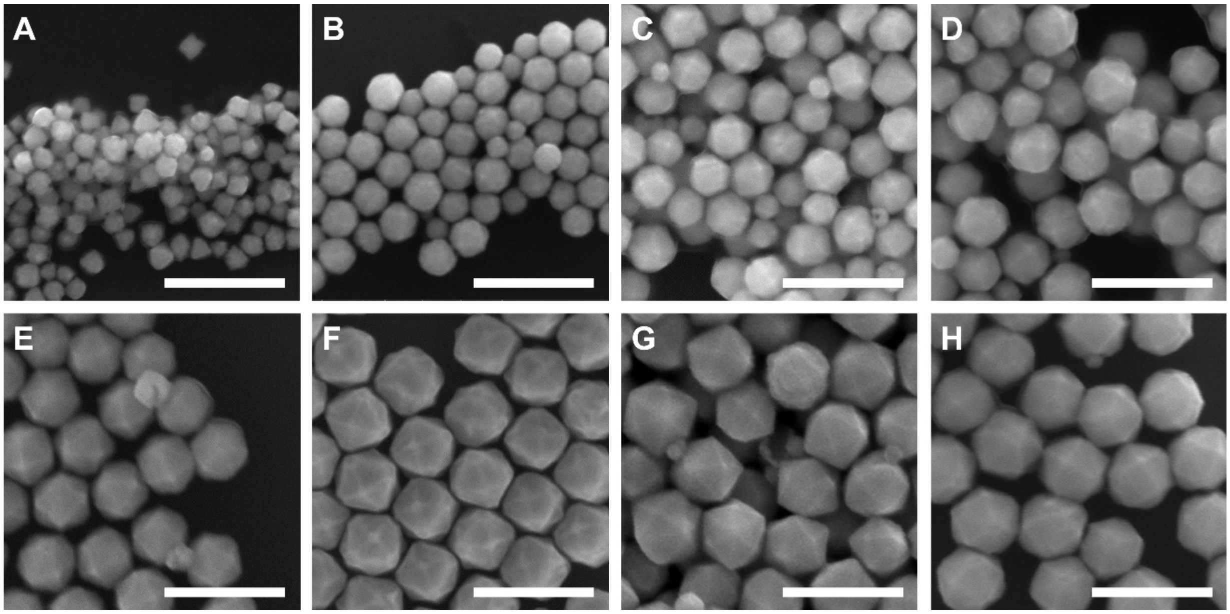

Figure S10. Point-in-time SEM images of Pd THH growth in dried BioXtra CTAB with acetone and iodide additives at (A) 60 min, (B) 120 min, (C) 180 min, (D) 240 min, (E) 360 min, (F) 600 min, (G) 1260 min, and (H) 1500 min. Scale bars: 500 nm.

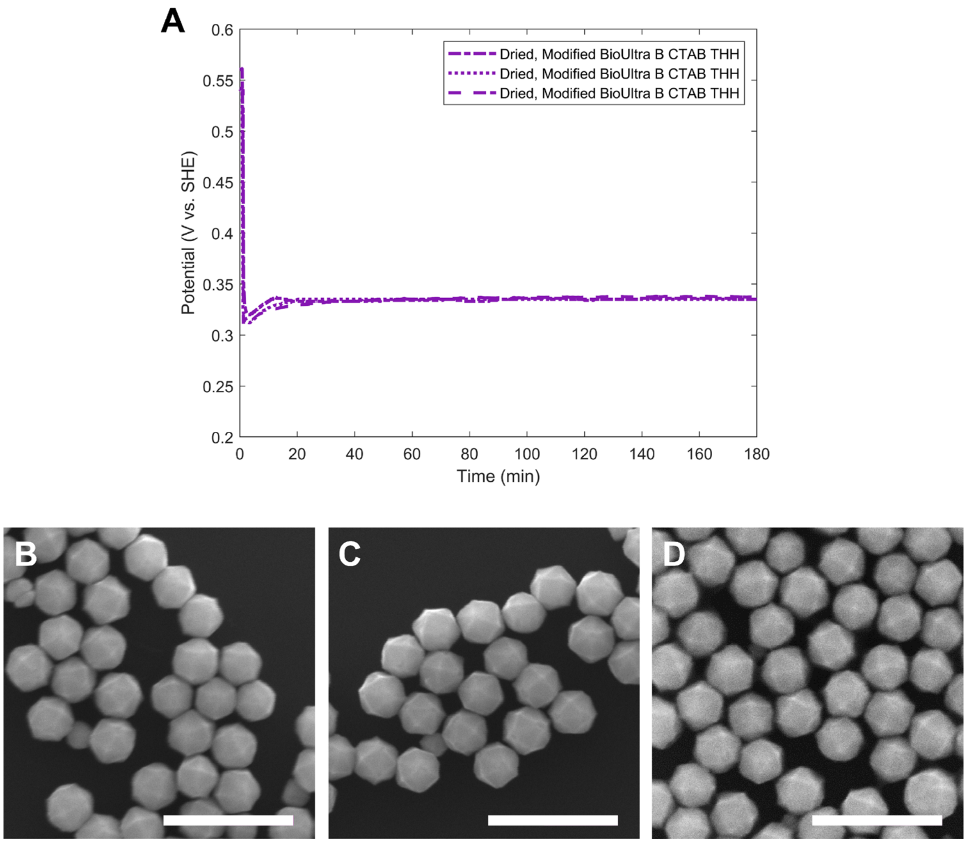

Figure S11. (A) Triplicate OCP measurements of Pd THH growth in BioUltra B CTAB. (B-D) SEM images of the Pd THH products from each growth solution. Scale bars: 500 nm. Purple dot-dash trace in panel A adapted with permission from ref. 33. Available under a Creative Commons Attribution-NonCommercial 3.0 Unported License. Copyright 2024 Halford and McDarby *et al.* Image of resulting growth solution is previously unpublished.

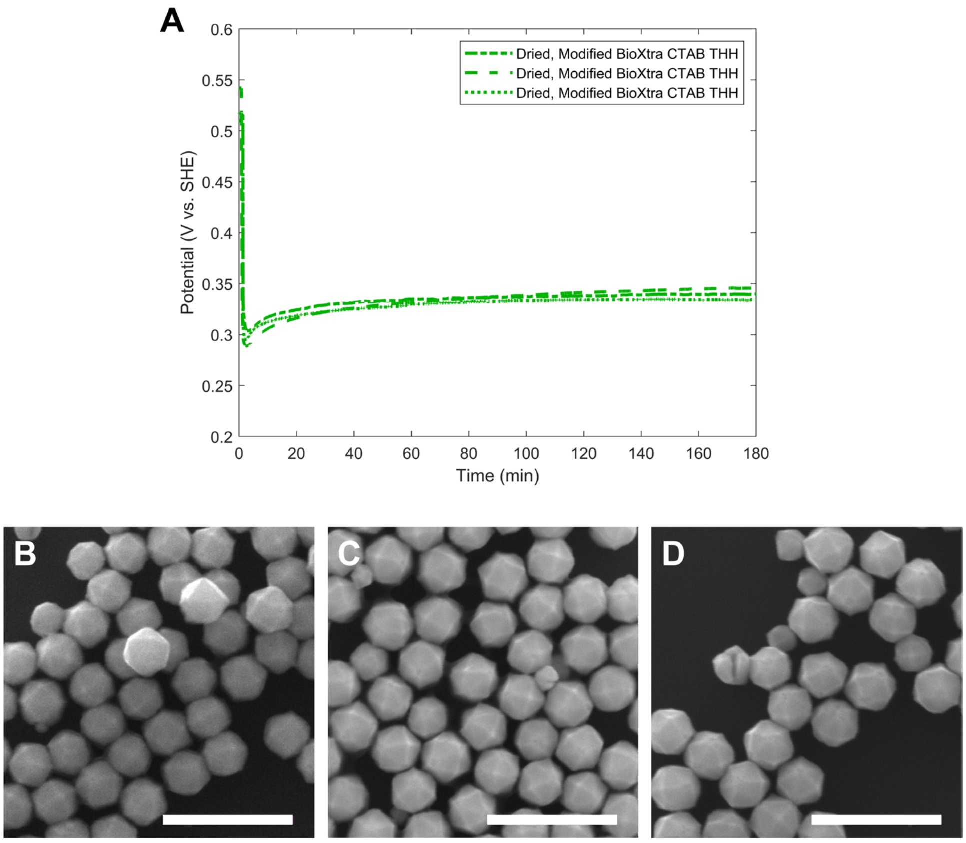

Figure S12. (A) Triplicate OCP measurements of Pd THH growth in BioXtra CTAB. (B-D) SEM images of the Pd THH products from each growth solution. Scale bars: 500 nm. Green dot-dash trace in panel A adapted with permission from ref. 33. Available under a Creative Commons Attribution-NonCommercial 3.0 Unported License. Copyright 2024 Halford and McDarby *et al.* Image of resulting growth solution is previously unpublished.

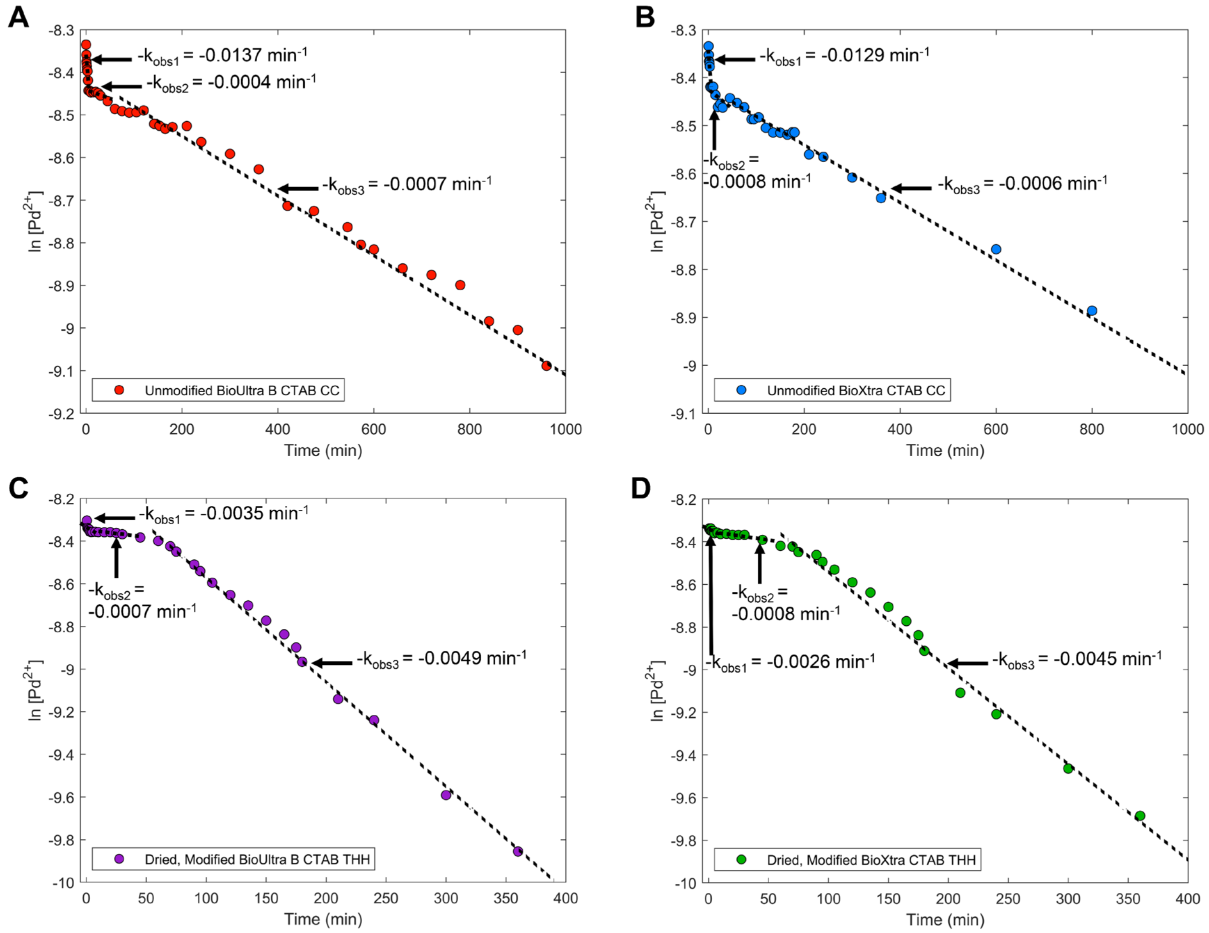

Figure S13. Plots with three-part linear fits of the ln[Pd^{2+}] (in M) over time for (A) BioUltra B CTAB CC, (B) BioXtra CTAB CC, (C) BioUltra B CTAB THH, and (D) BioXtra CTAB THH. Fits assume pseudo first-order reaction kinetics, and the labeled slope of the fit is the negative of the rate constant ($-k_{obs}$) for that portion of the reaction. The ln[Pd^{2+}] was calculated from the average [Pd^0] at each timepoint obtained from triplicate ICP samples.

Table S3. Input Parameters for OCP Kinetics Calculations for Growth of Pd CC and THH in 50 mM CTAB.

Reaction	Region	Range (min)	Time (min)	$[Pd^{2+}]_i$ (M)	$[AA]_i$ (M)	$[AA_{ox}]_i$ (M)
CC Unmodified BioUltra B CTAB	1	0-6	6	0.00024	0.00096	0
	2	6-28	22	0.000215	0.000935	0.000025
	3	28-180	152	0.000213	0.000933	0.000027
CC Unmodified BioXtra CTAB	1	0-6	6	0.00024	0.00096	0
	2	6-45	39	0.00022	0.00094	0.00002
	3	45-180	135	0.000215	0.000935	0.000025
THH Dried, Modified BioUltra B CTAB	1	0-10	10	0.00024	0.00096	0
	2	10-40	30	0.000235	0.000955	5E-06
	3	40-180	140	0.000229	0.000949	0.000011
THH Dried, Modified BioXtra CTAB	1	0-9	9	0.00024	0.00096	0
	2	9-50	41	0.000233	0.000953	7E-06
	3	50-180	130	0.000224	0.000944	0.000016

Table S4. Comparison Between Experimental and Calculated Rate Constants and OCP Parameters for Growth of Pd CC and THH in 50 mM CTAB.

Reaction	Region	Range (min)	k_{obs} (min^{-1}) Measured	R^2	k_{obs} (min^{-1}) Calculated	$Slope_{OCP}$ (mV min^{-1}) Measured	$Slope_{OCP}$ (mV min^{-1}) Calculated	Δ_{OCP} (mV) Measured	Δ_{OCP} (mV) Calculated
CC	1	0-6	0.0137	0.8720	NS[a]	-3.3333		-20	
Unmodified	2	6-28	0.0004	0.9663	NS	0.0545		1.2	
BioUltra B CTAB	3	28-180	0.0007	0.9879	0.0007[b]	0.0373[c]	0.0393	5.7[c]	6.0
CC	1	0-6	0.0129	0.8194	NS	0.8333		5	
Unmodified	2	6-45	0.0008	0.8325	NS	0.5897		23	
BioXtra CTAB	3	45-180	0.0006	0.9836	0.0010	0.0494	0.0402	6.7	5.4
THH	1	0-10	0.0035	0.9051	NS	0.0000		0	
Dried, Modified	2	10-40	0.0007	0.8990	NS	0.2667		8	
BioUltra B CTAB	3	40-180	0.0049	0.9937	0.0118	0.0452	0.0703	6.3	9.8
THH	1	0-9	0.0026	0.8082	NS	-0.7778		-7	
Dried, Modified	2	9-50	0.0008	0.8064	NS	0.4146		17	
BioXtra CTAB	3	50-180	0.0045	0.9760	0.0101	0.0564	0.0747	7.3	9.7

[a] NS = no solution

[b] Calculated value of k_{obs} is the average of fits to three OCP measurements.

[c] Measured values of $Slope_{OCP}$ and Δ_{OCP} for Region 3 are the average of three OCP measurements.

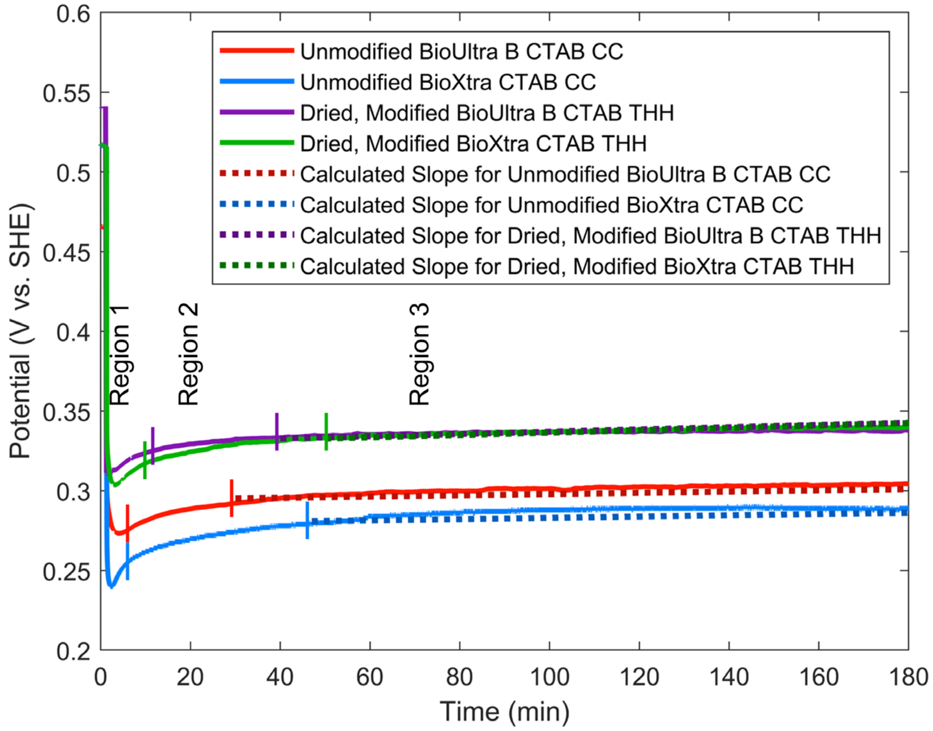

Figure S14. OCP measurements of reaction conditions that yield Pd CC (red, blue) or Pd THH (purple, green), plotted against calculated steady-state OCP slopes for each condition (dotted lines). R^2 values for the fit of the calculated slope to the experimental data are: 0.9344 (red), 0.9247 (blue), 0.9617 (purple), and 0.9652 (green). Plots are labeled with different regions of nanoparticle growth based on the approximate timing of the change in OCP slope (vertical lines). The timing of these changes in OCP slope corresponds to changes in the rate of Pd^{2+} ion reduction.

Table S5. Input Parameters for OCP Kinetics Calculations for Growth of Pd CC and THH in 50 mM CTAB, Incorporating ICP Kinetics Experimental Error.

Reaction	Region	Range (min)	Time (min)	$[Pd^{2+}]_i$ (M)	$[AA]_i$ (M)	$[AA_{ox}]_i$ (M)
CC Unmodified BioUltra B CTAB	1	0-6	6	0.00024	0.00096	0
	2	6-28	22	0.000214-0.000216	0.000934-0.000936	0.000026-0.000024
	3	28-180	152	0.000212-0.000214	0.000932-0.000934	0.000028-0.000026
CC Unmodified BioXtra CTAB	1	0-6	6	0.00024	0.00096	0
	2	6-45	39	0.000217-0.000223	0.000937-0.000943	0.000023-0.000017
	3	45-180	135	0.000213-0.000218	0.000933-0.000938	0.000027-0.000022
THH Dried, Modified BioUltra B CTAB	1	0-10	10	0.00024	0.00096	0
	2	10-40	30	0.000234-0.000236	0.000954-0.000956	0.000006-0.000004
	3	40-180	140	0.000226-0.000232	0.000946-0.000952	0.000014-0.000008
THH Dried, Modified BioXtra CTAB	1	0-9	9	0.00024	0.00096	0
	2	9-50	41	0.000232-0.000234	0.000952-0.000954	0.000008-0.000006
	3	50-180	130	0.000223-0.000225	0.000943-0.000945	0.000017-0.000015

Table S6. Comparison Between Experimental and Calculated Rate Constants and OCP Parameters for Growth of Pd CC and THH in 50 mM CTAB, Incorporating ICP Kinetics Experimental Error.

Reaction	Region	Range (min)	k_{obs} (min^{-1}) Measured	R^2	k_{obs} (min^{-1}) Calculated	$Slope_{OCP}$ (mV min^{-1}) Measured	$Slope_{OCP}$ (mV min^{-1}) Calculated	Δ_{OCP} (mV) Measured	Δ_{OCP} (mV) Calculated
CC	1	0-6	0.0130-0.0137	0.8358-0.8720	NS[a]	-3.3333		-20	
Unmodified	2	6-28	0.0004-0.0007	0.9012-0.9663	NS	0.0545		1.2	
BioUltra B CTAB	3	28-180	0.0006-0.0007	0.9277-0.9879	0.00047-0.00055	0.0373[b]	0.0371-0.0392	5.7[b]	5.6 - 6.0
CC	1	0-6	0.0124-0.0131	0.7855-0.8194	NS	0.8333		5	
Unmodified	2	6-45	0.0006-0.0008	0.8325-0.8904	NS	0.5897		23	
BioXtra CTAB	3	45-180	0.0006-0.0007	0.9533-0.9912	0.00069-0.00153	0.0494	0.0402-0.0470	6.7	5.4 - 6.3
THH	1	0-10	0.0035-0.0040	0.9051-0.9226	NS	0.0000		0	
Dried, Modified	2	10-40	0.0005-0.0007	0.8395-0.9300	NS	0.2667		8	
BioUltra B CTAB	3	40-180	0.0044-0.0055	0.9421-0.9937	0.01014-0.01054	0.0452	0.0668-0.0737	6.3	9.3 – 10.3
THH	1	0-9	0.0026-0.0029	0.8082-0.9006	NS	-0.7778		-7	
Dried, Modified	2	9-50	0.0007-0.0008	0.7799-0.9105	NS	0.4146		17	
BioXtra CTAB	3	50-180	0.0040-0.0051	0.9366-0.9760	0.01277-0.01313	0.0564	0.0727-0.0764	7.3	9.4 – 9.9

[a] NS = no solution

[b] Measured values of $Slope_{OCP}$ and Δ_{OCP} for Region 3 are the average of three OCP measurements.

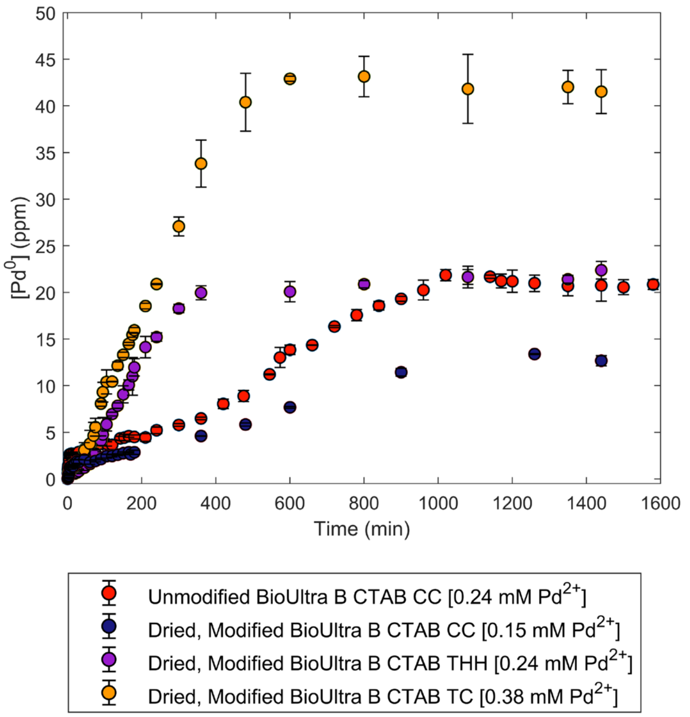

Figure S15. Unscaled ICP kinetics data for Pd nanoparticle growth in dried, modified 50 mM BioUltra Lot B CTAB with 0.38 mM (orange), 0.24 mM (purple), and 0.15 mM (blue) of Pd^{2+} in the growth solution and Pd CC growth in as-received BioUltra Lot B CTAB with 0.24 mM Pd^{2+} (red) in the growth solution.

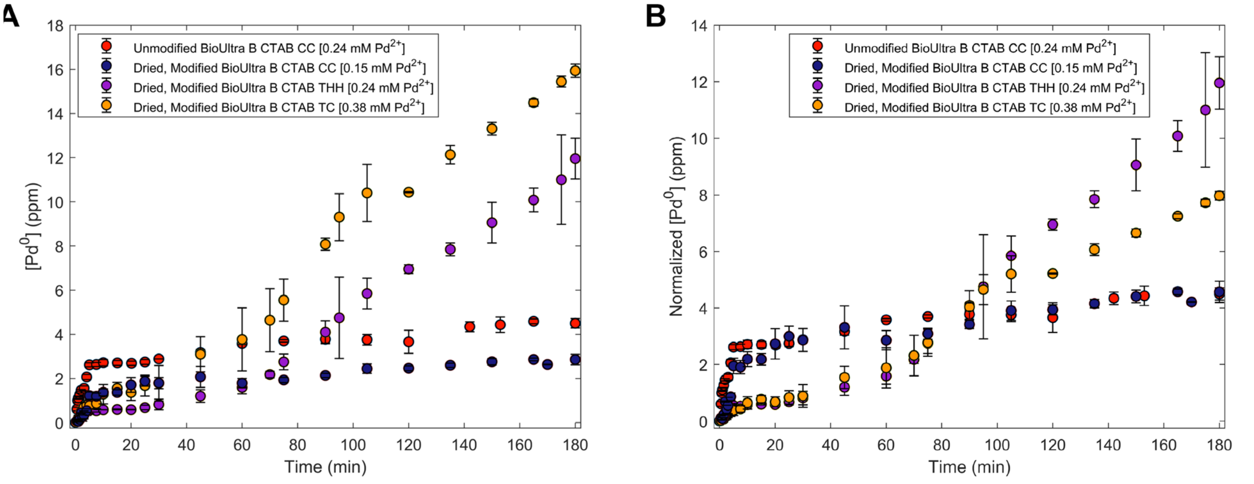

Figure S16. Zoomed-in plot of first 180 minutes of (A) unscaled and (B) normalized ICP kinetics data for Pd nanoparticle growth in dried, modified 50 mM BioUltra Lot B CTAB with 0.38 mM (orange), 0.24 mM (purple), and 0.15 mM (blue) of Pd^{2+} in the growth solution and Pd CC growth in as-received BioUltra Lot B CTAB with 0.24 mM Pd^{2+} (red) in the growth solution.

Table S7. Input Parameters for OCP Kinetics Calculations for Growth of Pd CC, THH, and TC with Varied Input [Pd^{2+}] in 50 mM Dried, Modified BioUltra B CTAB.

Reaction	Region	Range (min)	Time (min)	[Pd^{2+}]$_i$ (M)	[AA]$_i$ (M)	[AA_{ox}]$_i$ (M)
CC (0.15 mM Pd^{2+}) Dried, Modified BioUltra B CTAB	1	0-10	10	0.00015	0.00096	0
	2	10-70	60	0.000136	0.000946	0.000014
	3	70-180	110	0.000129	0.000939	0.000021
THH (0.24 mM Pd^{2+}) Dried, Modified BioUltra B CTAB	1	0-10	10	0.00024	0.00096	0
	2	10-40	30	0.000235	0.000955	5E-06
	3	40-180	140	0.000229	0.000949	0.000011
TC (0.38 mM Pd^{2+}) Dried, Modified BioUltra B CTAB	1	0-6	6	0.00038	0.00096	0
	2	18-100	82	0.000373	0.000953	7E-06
	3	100-180	80	0.000293	0.000873	0.000087

Table S8. Comparison Between Experimental and Calculated Rate Constants and OCP Parameters for Growth of Pd CC, THH, and TC with Varied Input [Pd^{2+}] in 50 mM Dried, Modified BioUltra B CTAB.

Reaction	Region	Range (min)	k_{obs} (min^{-1}) Measured	R^2	k_{obs} (min^{-1}) Calculated	$Slope_{OCP}$ (mV min^{-1}) Measured	$Slope_{OCP}$ (mV min^{-1}) Calculated	Δ_{OCP} (mV) Measured	Δ_{OCP} (mV) Calculated
CC (0.15 mM Pd^{2+})	1	0-10	0.0131	0.8801	NS[a]	0.5000		5	
Dried, Modified	2	10-70	0.0015	0.8717	NS	0.4167		25	
BioUltra B CTAB	3	70-180	0.0006	0.9924	0.00453	0.0909	0.0345	10	3.8
THH (0.24 mM Pd^{2+})	1	0-10	0.0035	0.9051	NS	0.0000		0	
Dried, Modified	2	10-40	0.0007	0.8990	NS	0.2667		8	
BioUltra B CTAB	3	40-180	0.0049	0.9937	0.0118	0.0452	0.0703	6.3	9.8
TC (0.38 mM Pd^{2+})	1	0-6	0.0025	0.9063	NS	-1.6667		-10	
Dried, Modified	2	18-100	0.0011	0.9693	NS	0.3659		30	
BioUltra B CTAB	3	100-180	0.0029	0.9528	0.00222	0.0625	0.0728	5	5.8

[a] NS = no solution

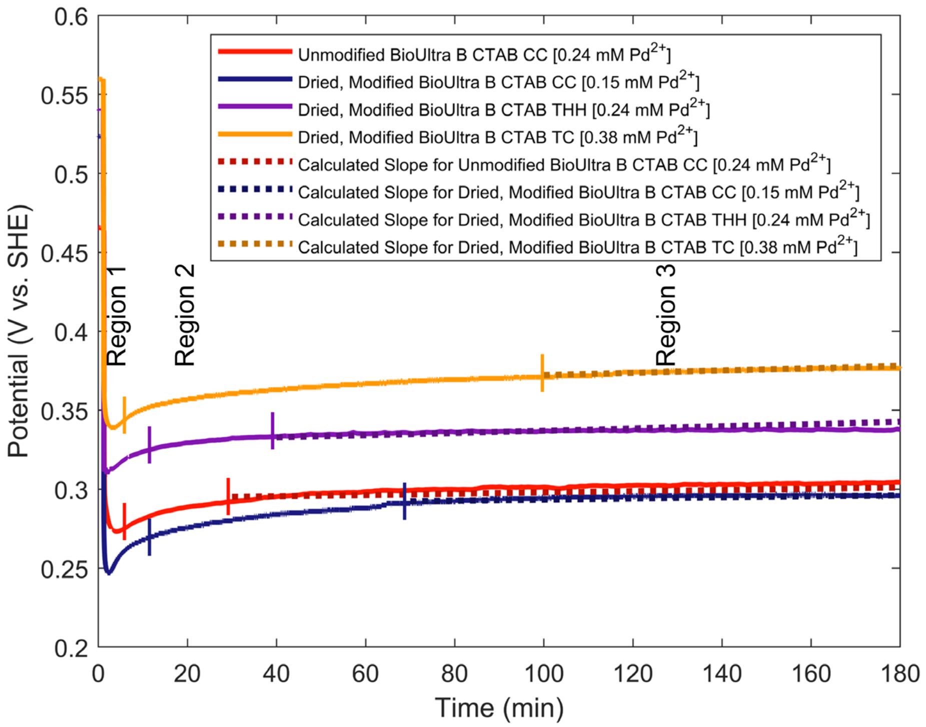

Figure S17. OCP measurements of Pd nanoparticle growth in as-received 50 mM BioUltra Lot B CTAB with 0.24 mM Pd^{2+} in the growth solution (red) and dried, modified 50 mM BioUltra Lot B CTAB with 0.15 mM (blue), 0.24 mM (purple), and 0.38 mM (orange) of Pd^{2+} in the growth solution, plotted against calculated steady-state OCP slopes for each condition (dotted lines). R^2 values for the fit of the calculated slope to the experimental data are: 0.9344 (red), 0.9273 (blue), 0.9617 (purple), and 0.9708 (orange). Plots are labeled with different regions of nanoparticle growth based on the approximate timing of the change in OCP slope (vertical lines). The timing of these changes in OCP slope corresponds to changes in the rate of Pd^{2+} ion reduction.

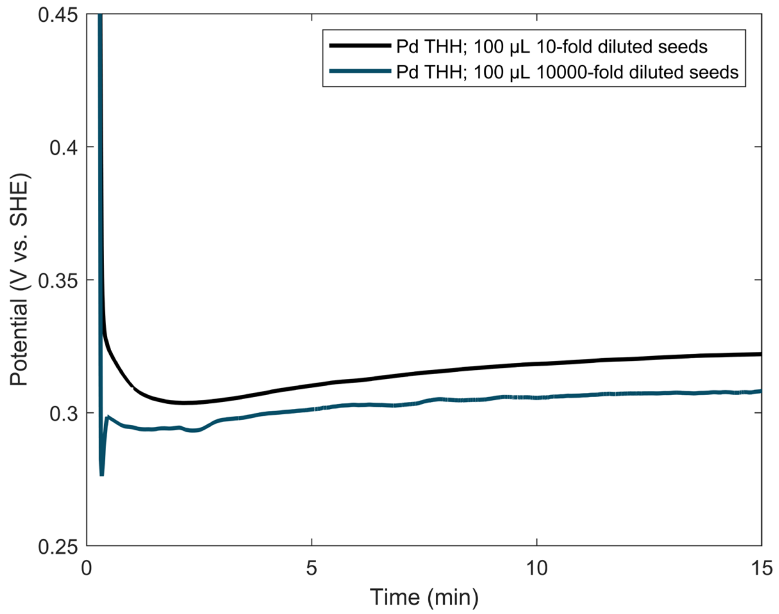

Figure S18. OCP measurements of particle growth reactions in dried, modified 50 mM BioXtra CTAB (THH-forming conditions) with different seed concentrations: 100 μL of 10-fold diluted seeds (standard condition) and 100 μL of 10000-fold diluted seeds.

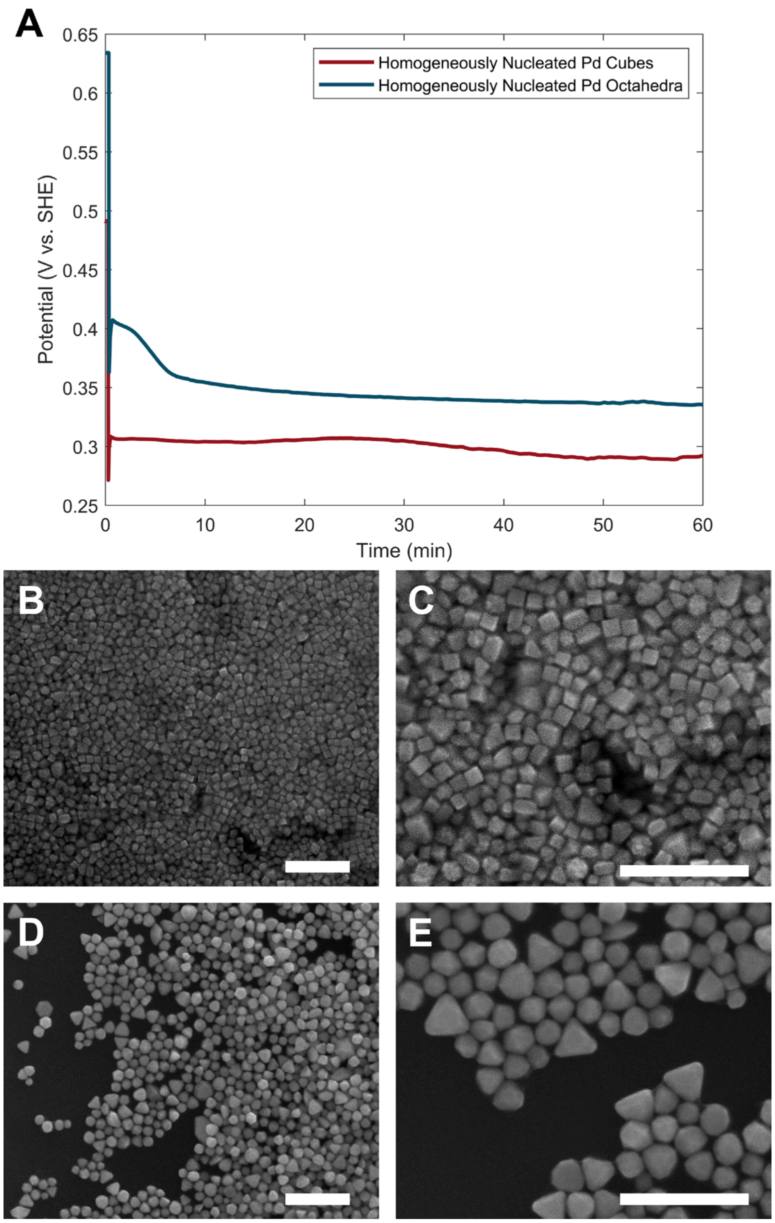

Figure S19. (A) OCP measurements of a homogeneously-nucleated Pd cube and octahedra synthesis with a 19:1 [AA]:[Pd^{2+}] ratio in the growth solution. (B) Low-magnification and (C) high-magnification SEM images of homogeneously-nucleated Pd cubes. (D) Low-magnification and (E) high-magnification SEM images of homogeneously-nucleated Pd octahedra. Scale bars: 500 nm.

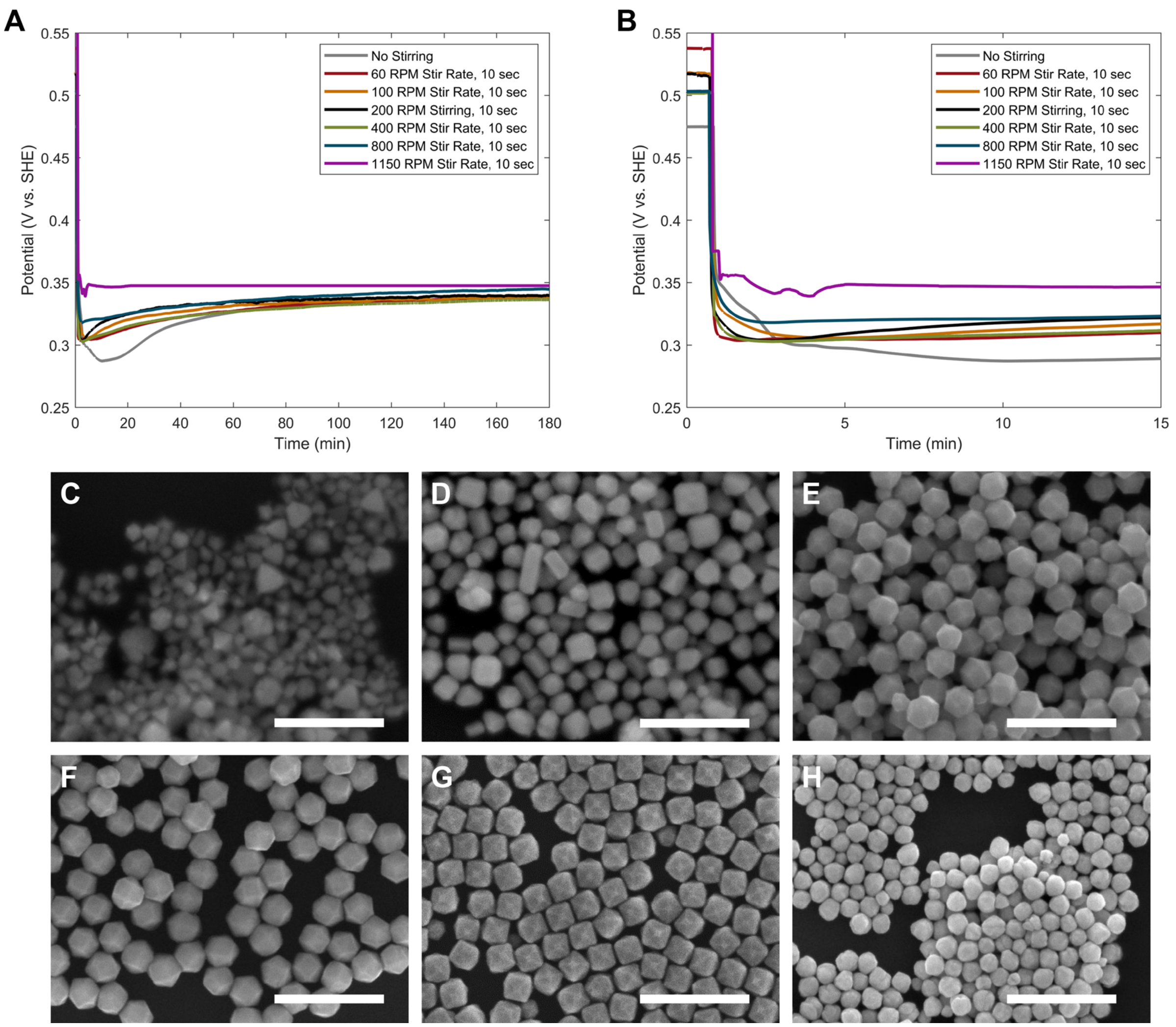

Figure S20. (A) OCP measurements of particle growth reactions in dried, modified 50 mM BioXtra CTAB (THH-forming conditions) with different stir rates for the first 10 seconds of the reaction. (B) Zoomed-in view of the first fifteen minutes of the OCP measurements from (A). (C–H) SEM images of Pd nanoparticle shapes formed during each stirring condition in (A): (C) no stirring, (D) 60 RPM stirring, (E) 100 RPM stirring, (F) 400 RPM stirring, (G) 800 RPM stirring, and (H) 1150 RPM stirring for the first 10 seconds of the reaction. Scale bars: 500 nm.

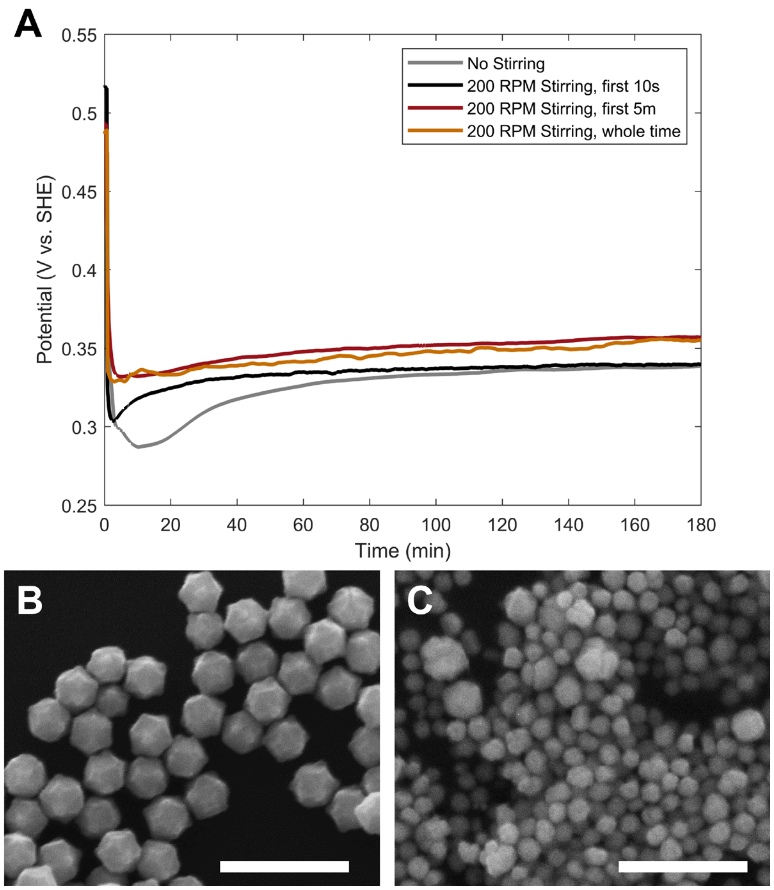

Figure S21. (A) OCP measurements of Pd particle growth with no stirring (gray), 200 RPM stirring for the first 10 seconds of reaction (black; standard condition; THH-forming), 200 RPM stirring for the first 5 minutes of reaction (red), and 200 RPM stirring throughout the entire measurement (orange). (B-C) SEM images of Pd particle products with 200 RPM stirring for (B) 5 minutes and (C) throughout the reaction. Scale bars: 500 nm.

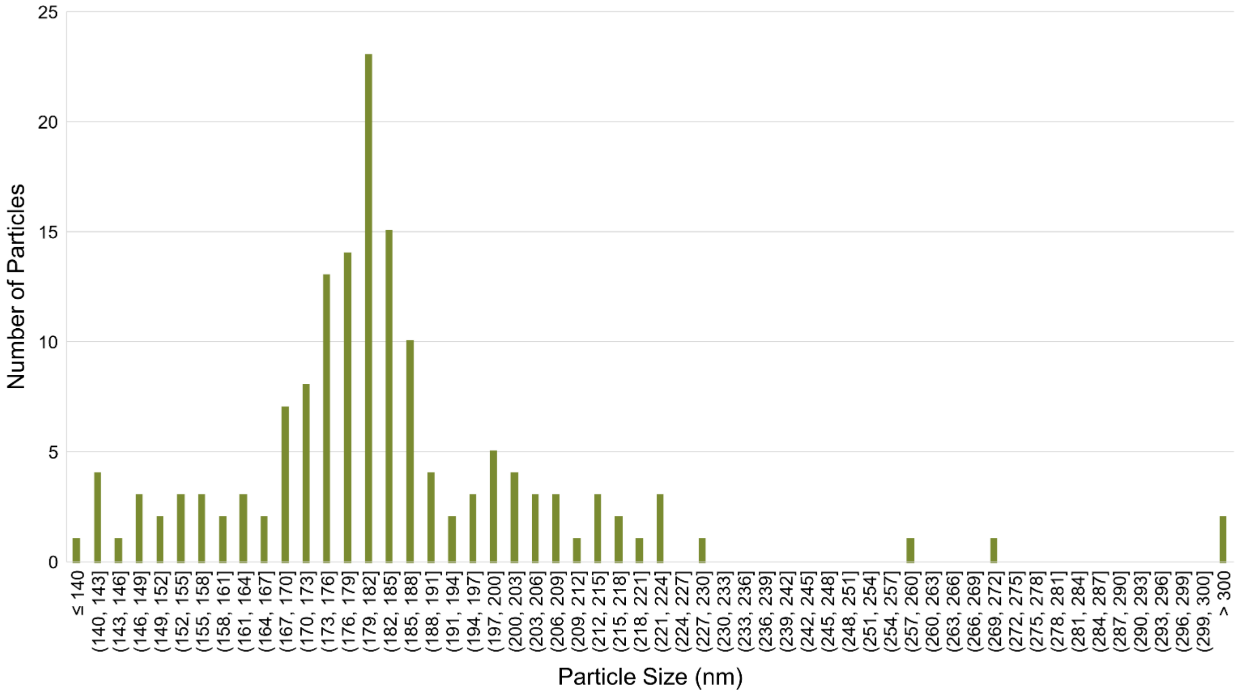

Figure S22. Histogram of nanoparticle size distribution for Pd THH produced with 0.24 mM Pd^{2+} in 150 mM BioUltra Lot B CTAB with added iodide. Average particle size was 183 ± 29 nm (150 particles measured). Particles were measured diagonally.

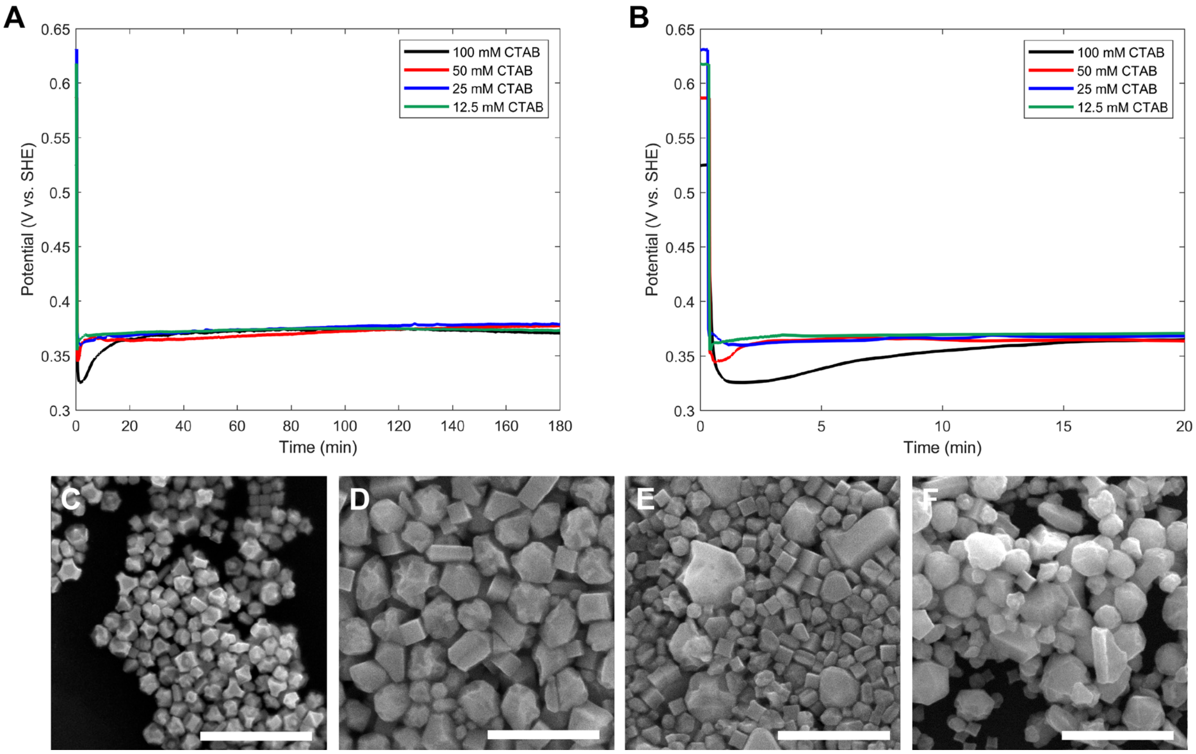

Figure S23. (A) OCP measurements of Pd nanoparticle growth in a minimally complicated growth solution made with 100 mM, 50 mM, 25 mM, and 12.5 mM CTAB. (B) Zoomed-in view of the first twenty minutes of the OCP measurements from (A). (C-F) SEM images of Pd nanoparticles grown in (C) 100 mM CTAB, (D) 50 mM CTAB, (E) 25 mM CTAB, and (F) 12.5 mM CTAB. Scale bars: 500 nm.

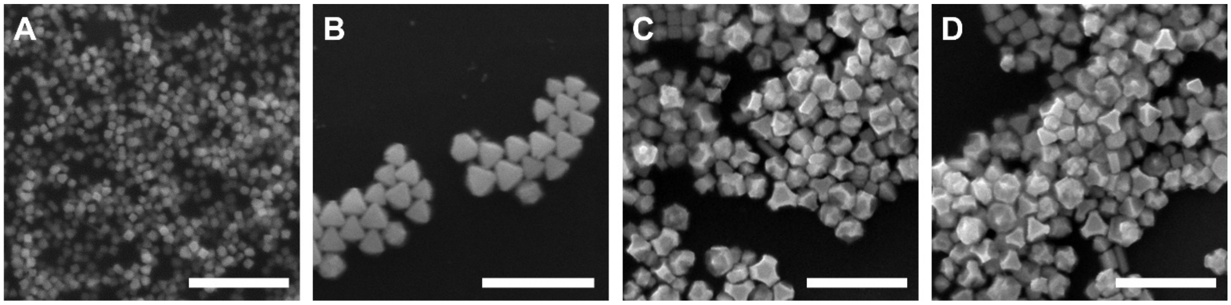

Figure S24. Point-in-time SEM images of Pd nanoparticle growth in a minimally-complicated growth solution made with 100 mM CTAB at (A) 15 min, (B) 60 min, and (C) 120 min. Scale bars: 250 nm.

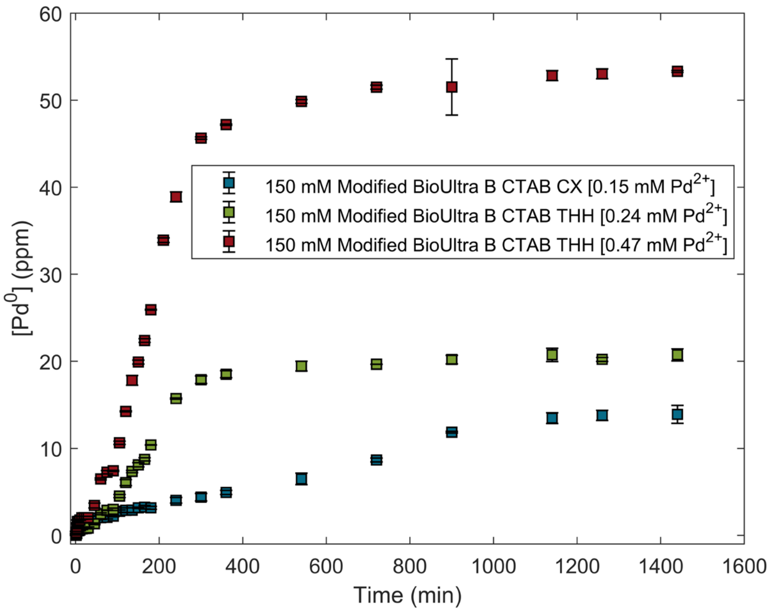

Figure S25. Unscaled ICP kinetics data for Pd nanoparticle growth in 150 mM BioUltra Lot B CTAB with 0.47 mM (red), 0.24 mM (green), and 0.15 mM (teal) of Pd^{2+} in the growth solution.

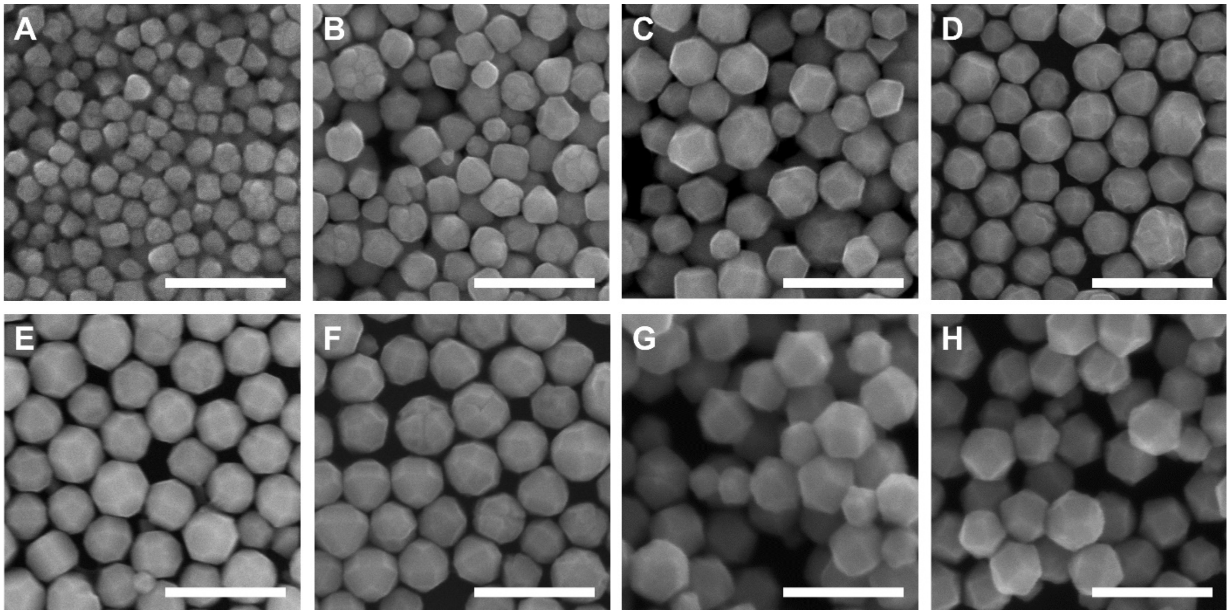

Figure S26. Point-in-time SEM images of Pd THH growth with 0.47 mM Pd^{2+} in 150 mM BioUltra Lot B CTAB at (A) 120 min, (B) 180 min, (C) 240 min, (D) 300 min, (E) 360 min, (F) 420 min, (G) 600 min, and (H) 1260 min. Scale bars: 500 nm.

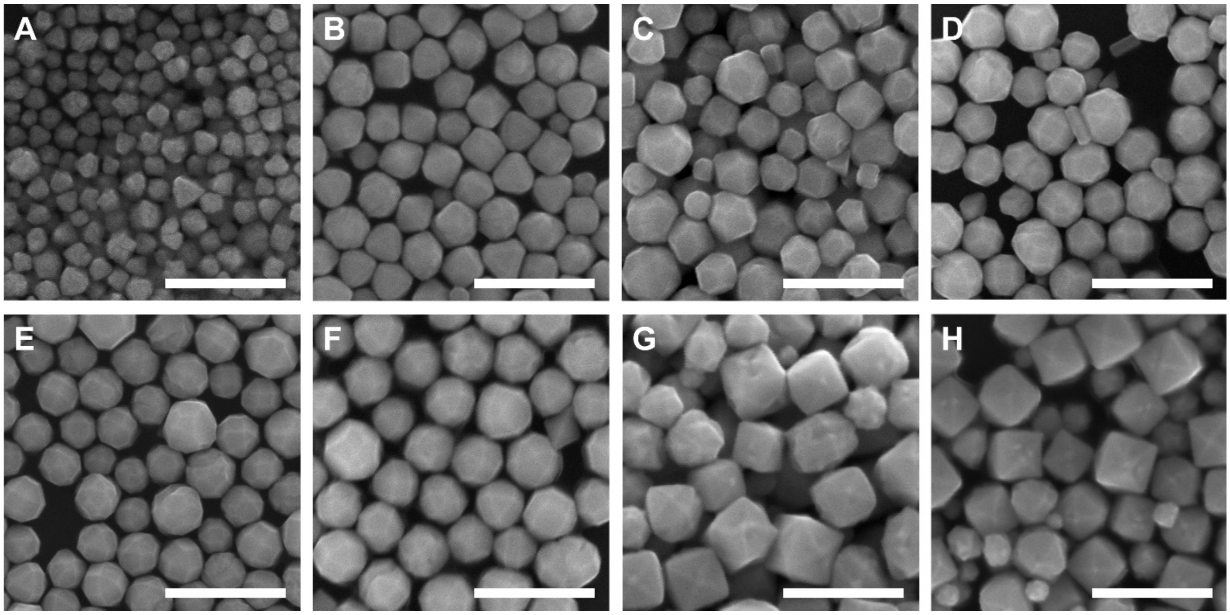

Figure S27. Point-in-time SEM images of Pd THH growth with 0.24 mM Pd^{2+} in 150 mM BioUltra Lot B CTAB at (A) 120 min, (B) 180 min, (C) 240 min, (D) 300 min, (E) 360 min, (F) 420 min, (G) 600 min, and (H) 1260 min. Scale bars: 500 nm.

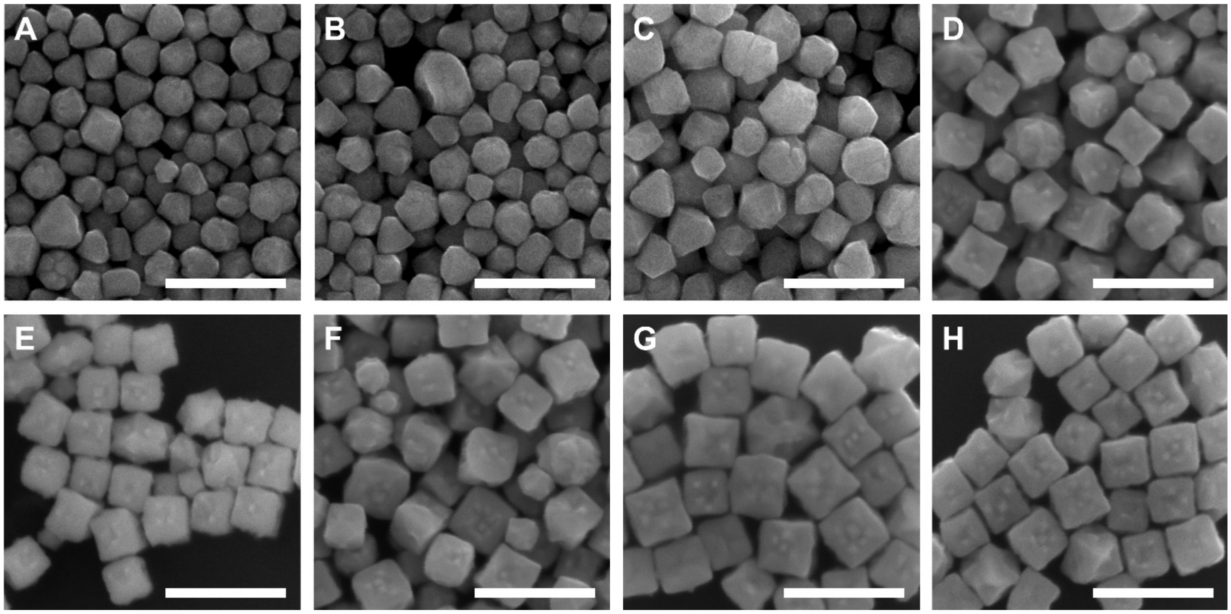

Figure S28. Point-in-time SEM images of Pd concave-convex nanoparticle growth with 0.15 mM Pd^{2+} in 150 mM BioUltra Lot B CTAB at (A) 120 min, (B) 180 min, (C) 240 min, (D) 300 min, (E) 360 min, (F) 420 min, (G) 600 min, and (H) 1260 min. Scale bars: 500 nm.

Table S9. Input Parameters for OCP Kinetics Calculations for Growth of Pd CX and THH with Varied Input [Pd^{2+}] in 150 mM BioUltra B CTAB.

Reaction	Region	Range (min)	Time (min)	[Pd^{2+}]$_i$ [M]	[AA]$_i$ [M]	[AA_{ox}]$_i$ [M]
CX (0.15 mM Pd^{2+}) 150 mM Modified BioUltra B CTAB	1	0-10	10	0.00015	0.00096	0
	2	10-40	30	0.000137	0.000947	0.000013
	3	40-180	140	0.000129	0.000939	0.000021
THH (0.24 mM Pd^{2+}) 150 mM Modified BioUltra B CTAB	1	0-14	14	0.00024	0.00096	0
	2	14-50	36	0.000233	0.000953	7E-06
	3	50-180	130	0.000227	0.000947	0.000013
THH (0.47 mM Pd^{2+}) 150 mM Modified BioUltra B CTAB	1	0-7	7	0.00047	0.00096	0
	2	7-25	18	0.000453	0.000943	0.000017
	3	25-180	155	0.000449	0.000939	0.000021

Table S10. Comparison Between Experimental and Calculated Rate Constants and OCP Parameters for Growth of Pd CX and THH with Varied Input [Pd^{2+}] in 150 mM BioUltra B CTAB.

Reaction	Region	Range (min)	k_{obs} (min^{-1}) Measured	R^2	k_{obs} (min^{-1}) Calculated	$Slope_{OCP}$ (mV min^{-1}) Measured	$Slope_{OCP}$ (mV min^{-1}) Calculated	Δ_{OCP} (mV) Measured	Δ_{OCP} (mV) Calculated
CX (0.15 mM Pd^{2+})	1	0-10	0.0065	0.8142	NS[a]	-1.5000		-15	
150 mM Modified	2	10-40	0.0002	0.6741	NS	0.5000		15	
BioUltra B CTAB	3	40-180	0.0009	0.9848	NS	0.1429	0.0396	20	5.5
THH (0.24 mM Pd^{2+})	1	0-14	0.0030	0.9122	NS	-0.7143		-10	
150 mM Modified	2	14-50	0.0008	0.8915	NS	0.4167		15	
BioUltra B CTAB	3	50-180	0.0044	0.9719	NS	0.1154	0.0769	15	10
THH (0.47 mM Pd^{2+})	1	0-7	0.0034	0.7506	NS	0.4286		3	
150 mM Modified	2	7-25	0.0010	0.7702	NS	1.2222		22	
BioUltra B CTAB	3	25-180	0.0045	0.9774	NS	0.1935	0.0741	30	11.5

[a] NS = no solution

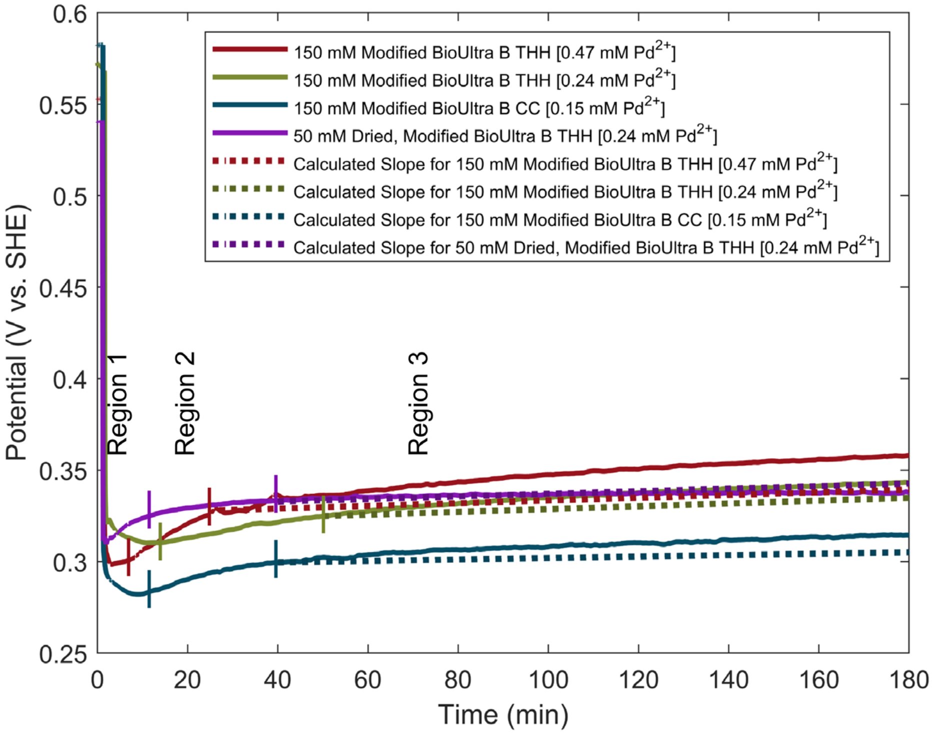

Figure S29. OCP measurements of reactions in iodide-modified 150 mM BioUltra Lot B CTAB with 0.47 mM (red), 0.24 mM (green) and 0.15 mM (teal) of Pd^{2+} in the growth solution, and dried, modified 50 mM BioUltra Lot B CTAB with 0.24 mM Pd^{2+} (purple), plotted against calculated steady-state OCP slopes for each condition (dotted lines). R^2 values for the fit of the calculated slope to the experimental data are: 0.4955 (red), 0.7952 (green), 0.6919 (blue), and 0.9617 (purple). Plots are labeled with different regions of nanoparticle growth based on the approximate timing of the change in OCP slope (vertical lines). The timing of these changes in OCP slope corresponds to changes in the rate of Pd^{2+} ion reduction.

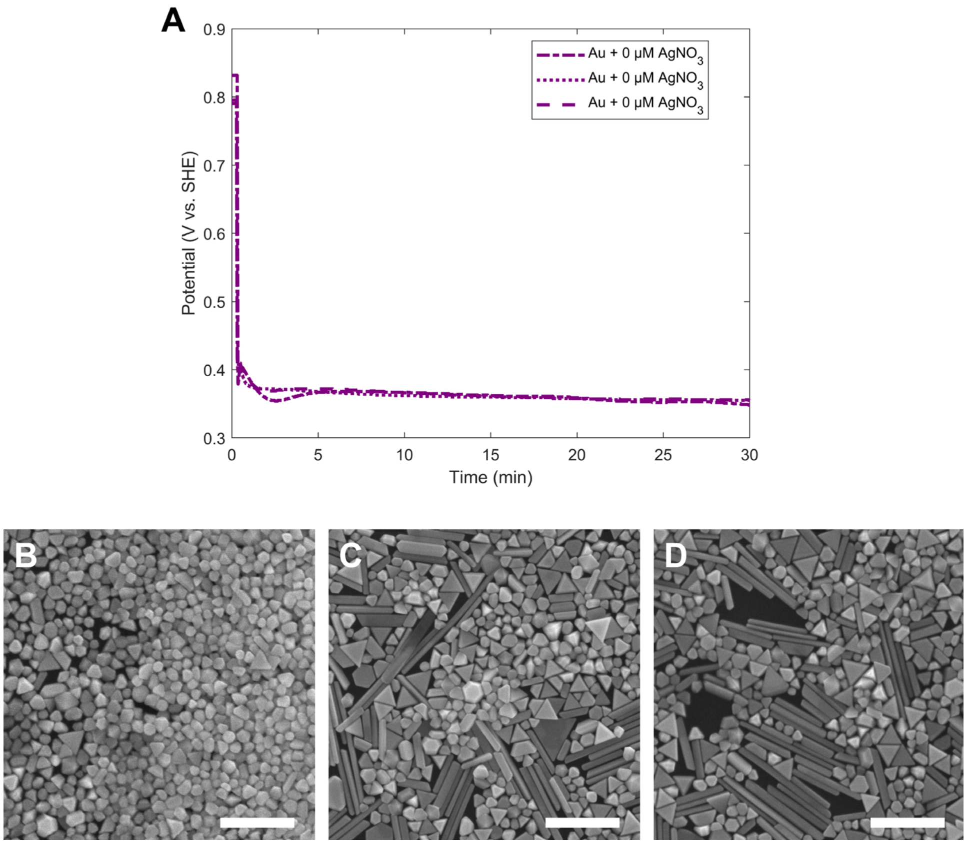

Figure S30. (A) Triplicate OCP measurements of Au nanoparticle growth with no added Ag^+. (B-D) SEM images of the {111}-faceted Au nanoparticle products from each growth solution. Scale bars: 500 nm.

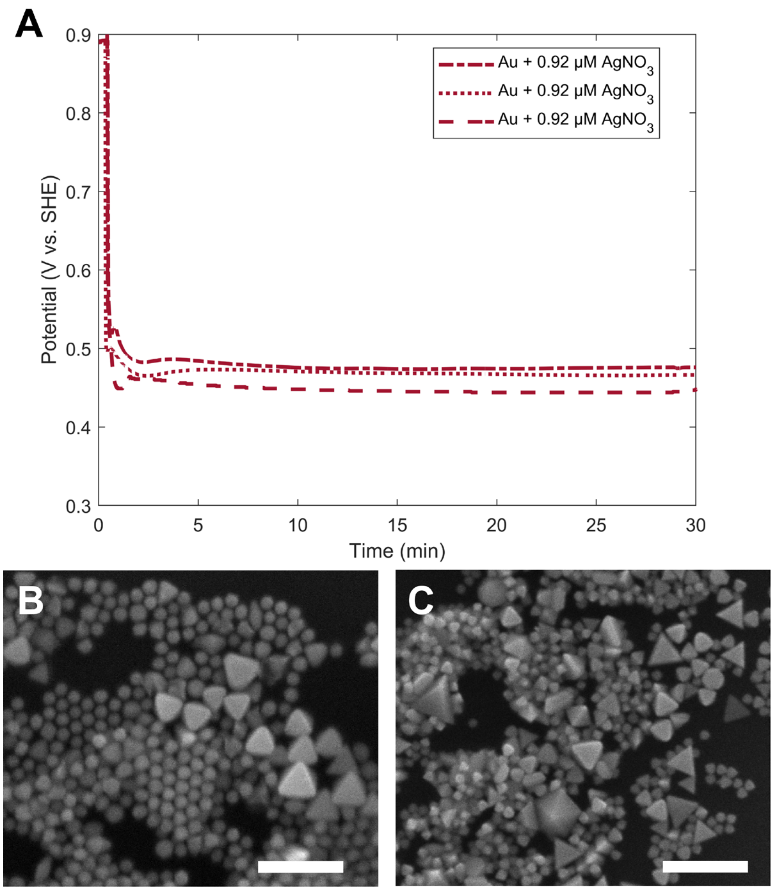

Figure S31. (A) Triplicate OCP measurements of Au nanoparticle growth with 0.92 μM added Ag^+ in the growth solution. (B-C) SEM images of the {111} octahedral (Ag)Au nanoparticle products from two of the three growth solutions. See Figure 5C for the third representative SEM image. Scale bars: 500 nm.

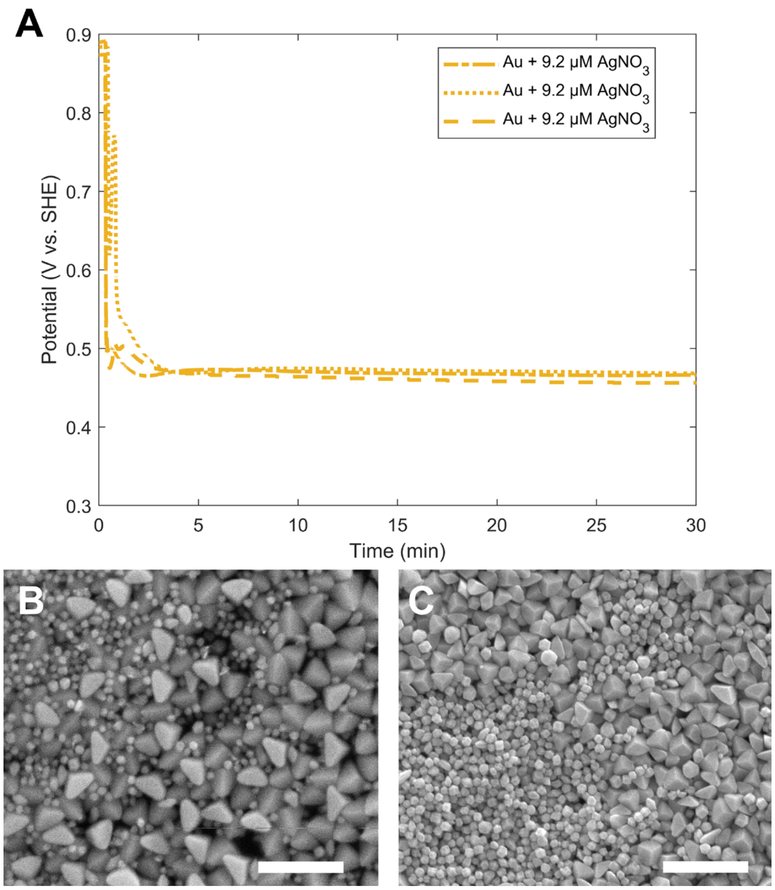

Figure S32. (A) Triplicate OCP measurements of Au nanoparticle growth with 9.2 µM added Ag^+ in the growth solution. (B-C) SEM images of the {110} rhombic dodecahedral and large {110} bipyramidal (Ag)Au nanoparticle products from two of the three growth solutions. See Figure 5D for the third representative SEM image. Scale bars: 500 nm.

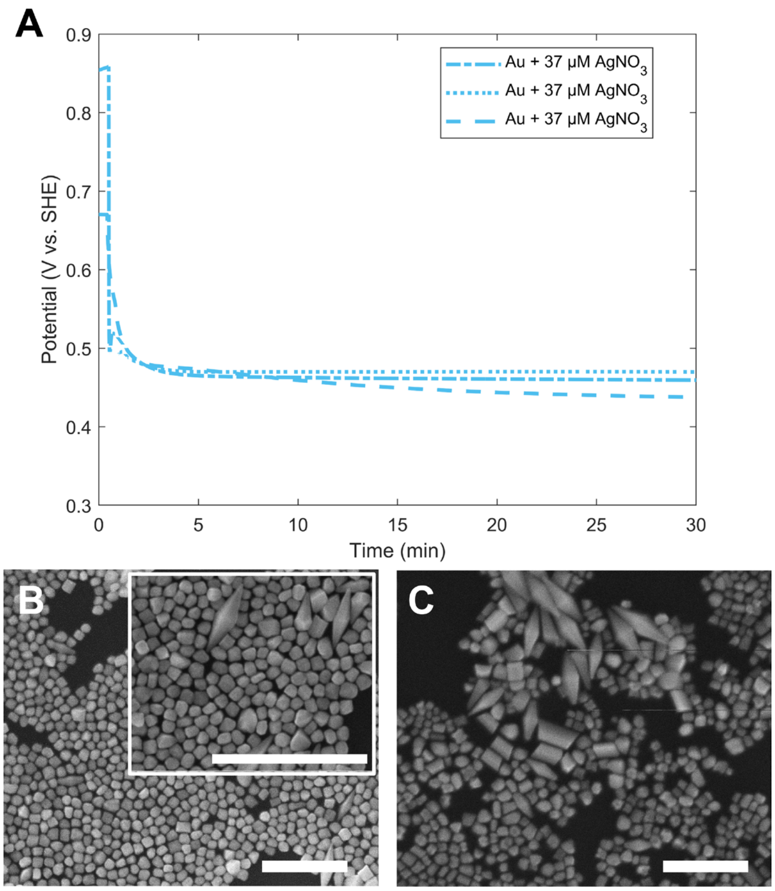

Figure S33. (A) Triplicate OCP measurements of Au nanoparticle growth with 37 μM added Ag^+ in the growth solution. (B-C) SEM images of the {310} (Ag)Au truncated ditetragonal prism nanoparticle products from two of the three growth solutions. See Figure 5E for the third representative SEM image. Scale bars: 500 nm.

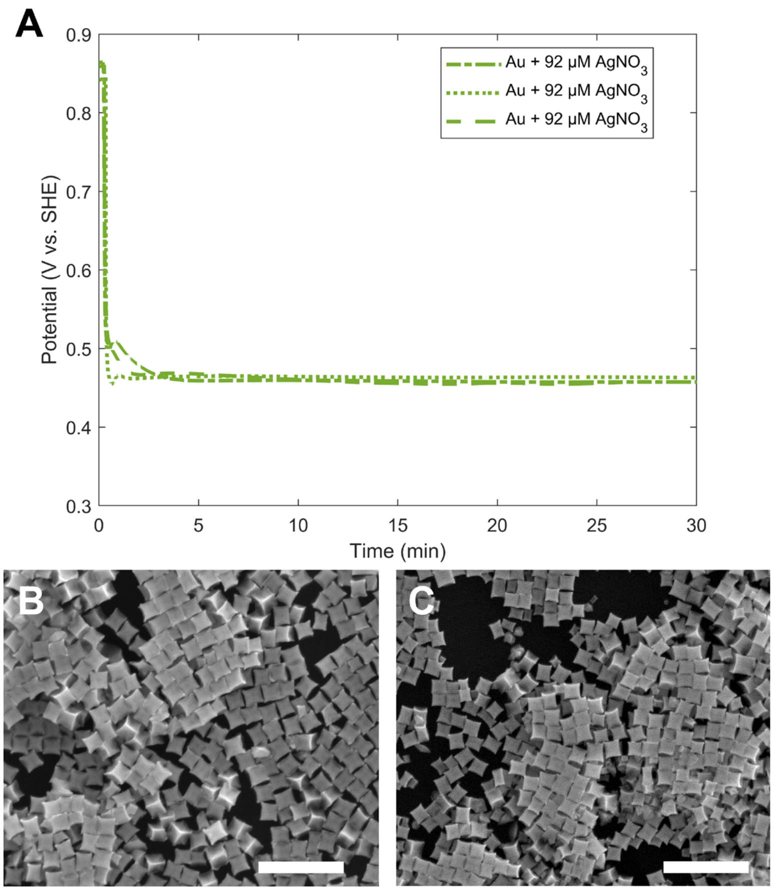

Figure S34. (A) Triplicate OCP measurements of Au nanoparticle growth with 92 µM added Ag^+ in the growth solution. (B-C) SEM images of the {720} (Ag)Au concave cubic (CC) nanoparticle products from two of the three growth solutions. See Figure 5F for the third representative SEM image. Scale bars: 500 nm.

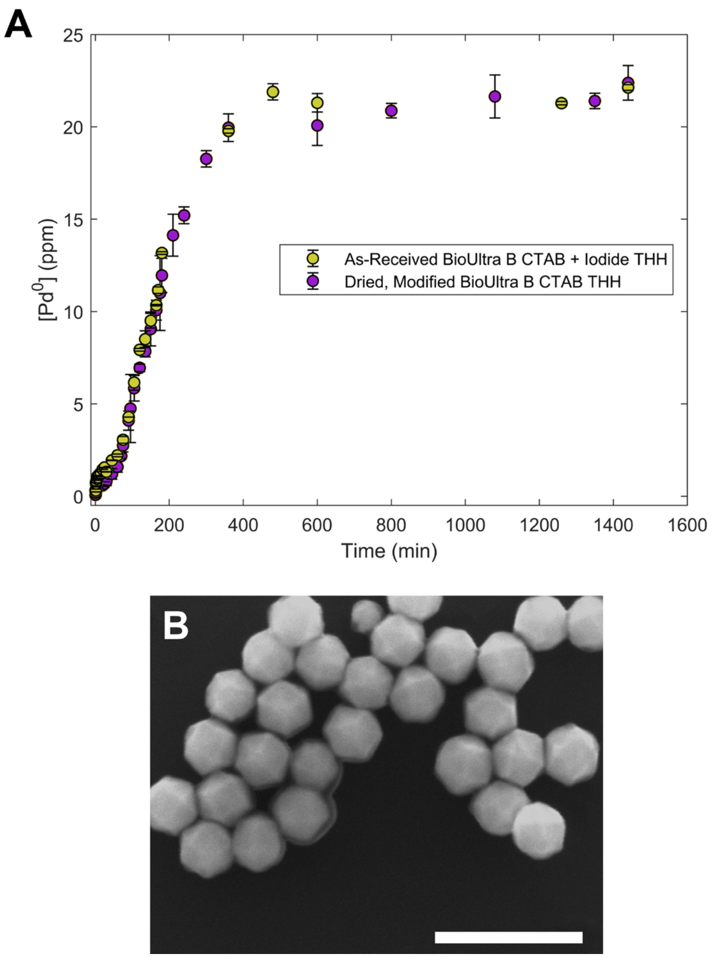

Figure S35. (A) ICP kinetics data for Pd nanoparticle growth in as-received 50 mM BioUltra Lot B CTAB with 0.068 M acetone and 0.68 μM iodide added to the growth solution and for Pd THH growth in dried 50 mM BioUltra Lot B CTAB with 0.26 M acetone and 0.68 μM iodide. (B) SEM image of truncated Pd THH in as-received 50 mM BioUltra Lot B CTAB with 0.068 M acetone and 0.68 μM iodide. Scale bar: 500 nm.

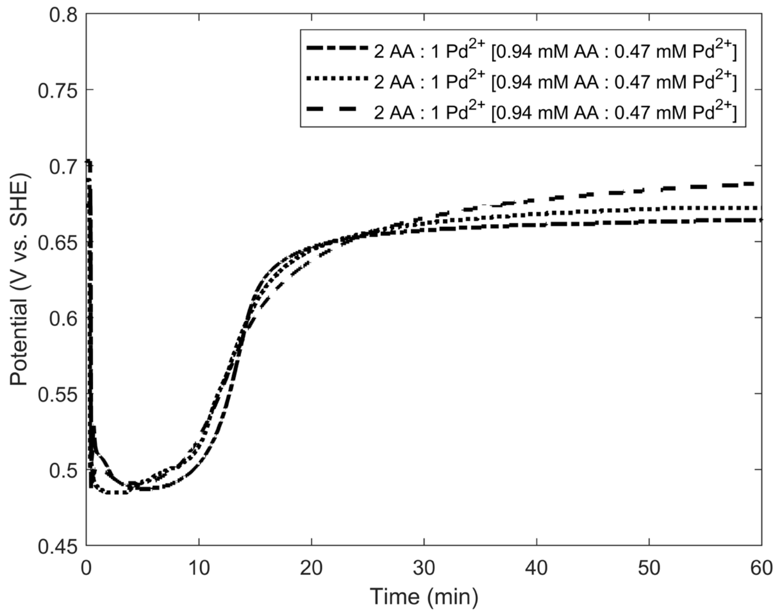

Figure S36. Triplicate OCP measurements of Pd particle growth in $CTAHSO_4$ with 0.94 mM AA and 0.47 mM Pd^{2+} (2:1 [AA]/[Pd^{2+}] ratio).

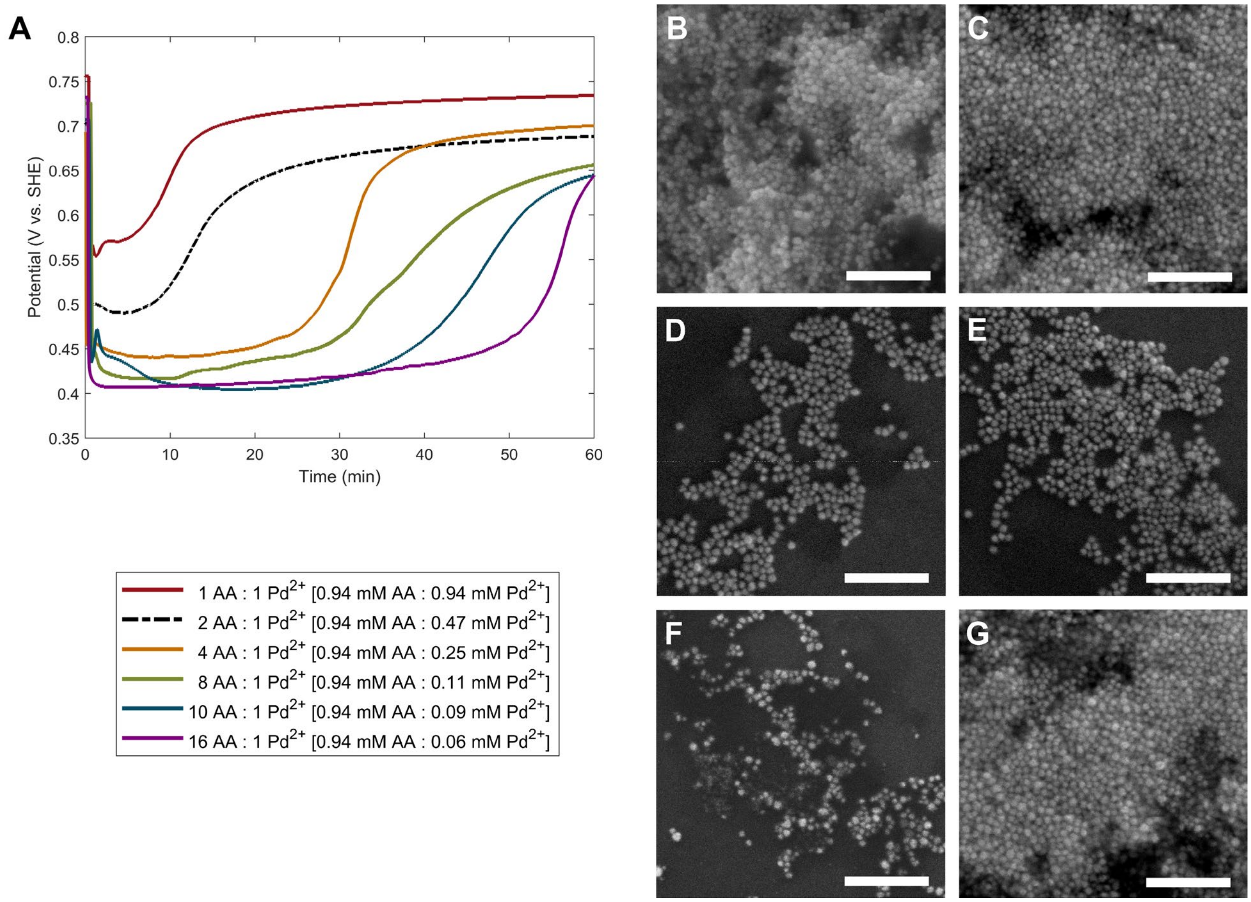

Figure S37. (A) OCP measurements of Pd particle growth solutions in $CTAHSO_4$ with varied ratios of [AA]/[Pd^{2+}], with the ratio controlled by varying [Pd^{2+}] while [AA] is fixed at 0.94 mM. (B-G) SEM images of Pd particle growth in $CTAHSO_4$ with a fixed 0.94 mM AA concentration and (B) 0.94 mM Pd^{2+} (1:1 [AA]/[Pd^{2+}] ratio), (C) 0.47 mM Pd^{2+} (2:1 [AA]/[Pd^{2+}] ratio), (D) 0.25 mM Pd^{2+} (4:1 [AA]/[Pd^{2+}] ratio), (E) 0.11 mM Pd^{2+} (8:1 [AA]/[Pd^{2+}] ratio), (F) 0.09 mM Pd^{2+} (10:1 [AA]/[Pd^{2+}] ratio), and (G) 0.06 mM Pd^{2+} (16:1 [AA]/[Pd^{2+}] ratio). Scale bars: 500 nm.

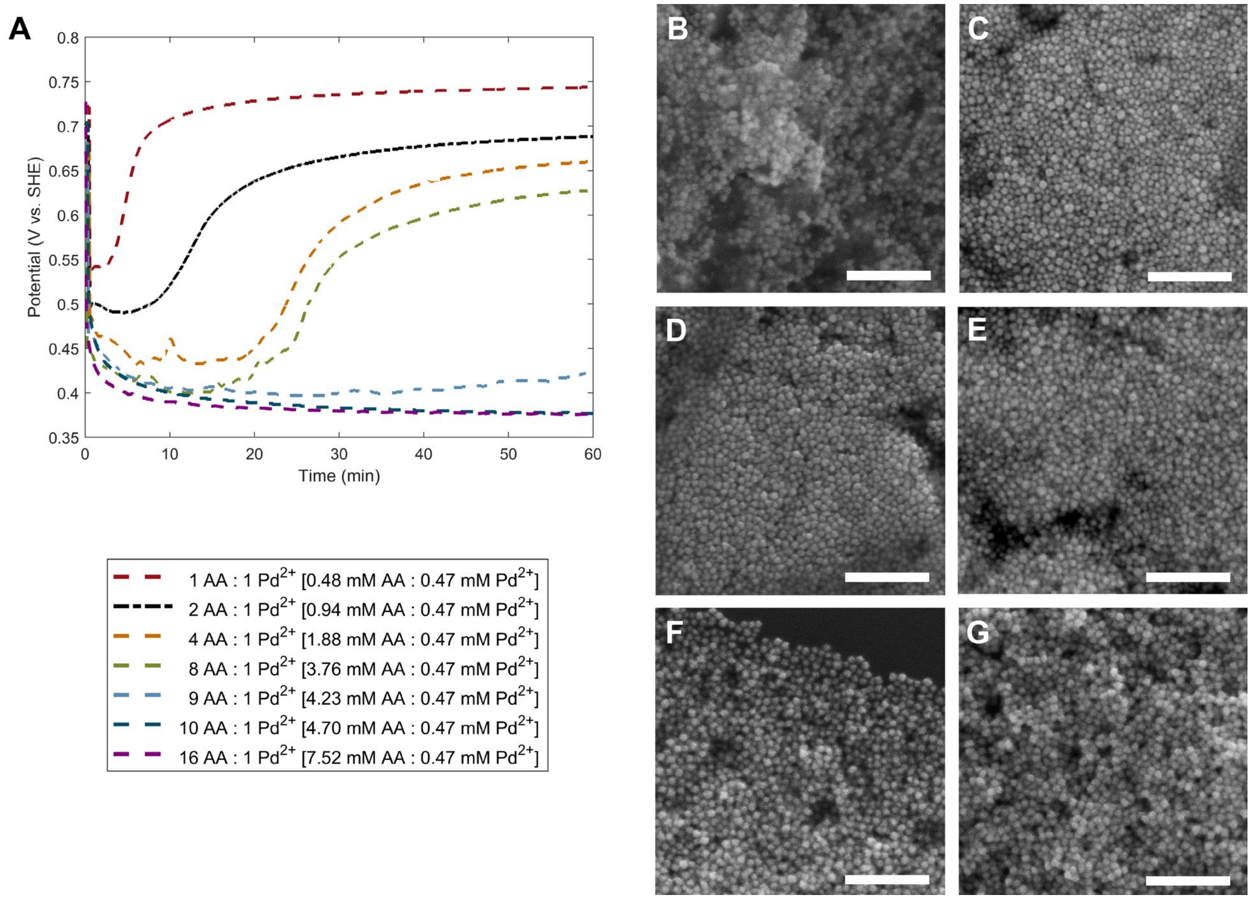

Figure S38. (A) OCP measurements of Pd particle growth solutions in $CTAHSO_4$ with varied ratios of [AA]/[Pd^{2+}], with the ratio controlled by varying [AA] while [Pd^{2+}] is fixed at 0.47 mM. (B-G) SEM images of Pd particle growth in $CTAHSO_4$ with a fixed 0.47 mM Pd^{2+} concentration and (B) 0.48 mM AA (1:1 [AA]/[Pd^{2+}] ratio), (C) 1.88 mM AA (4:1 [AA]/[Pd^{2+}] ratio), (D) 3.76 mM AA (8:1 [AA]/[Pd^{2+}] ratio), (E) 4.23 mM AA (9:1 [AA]/[Pd^{2+}] ratio), (F) 4.70 mM AA (10:1 [AA]/[Pd^{2+}] ratio), and (G) 7.52 mM AA (16:1 [AA]/[Pd^{2+}] ratio). See Figure S37C for SEM image of Pd particles with 2:1 [AA]/[Pd^{2+}] ratio. Scale bars: 500 nm.

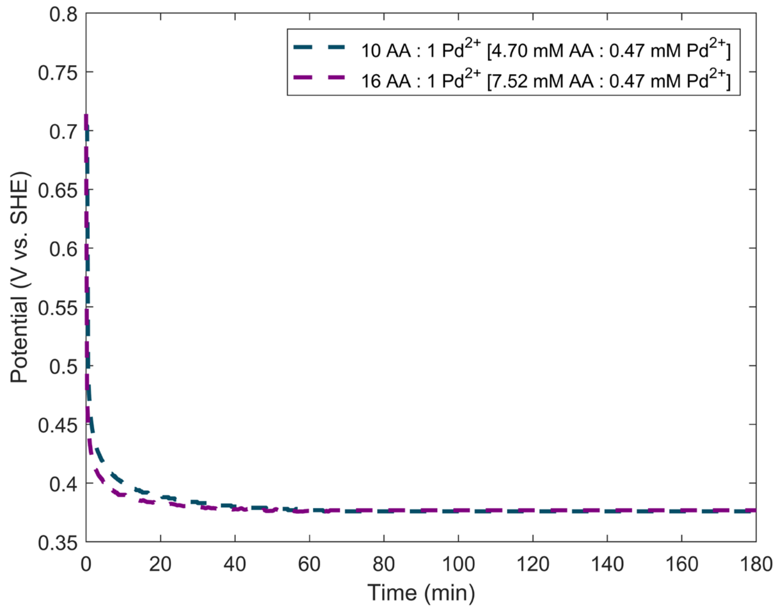

Figure S39. OCP measurements of Pd particle growth in $CTAHSO_4$ with 4.70 mM AA and 0.47 mM Pd^{2+} (10:1 [AA]/[Pd^{2+}] ratio) and with 7.52 mM AA and 0.47 mM Pd^{2+} (16:1 [AA]/[Pd^{2+}] ratio). Measurements were taken for 180 minutes, vs. the standard 60-minute measurement period for this set of reaction conditions.

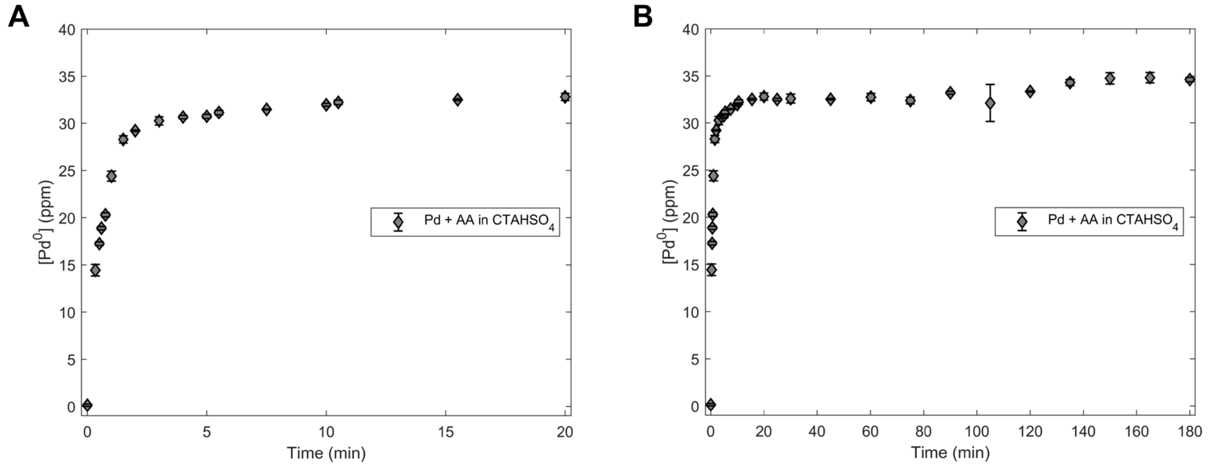

Figure S40. ICP kinetics data for Pd nanoparticle growth in $CTAHSO_4$ with 0.94 mM AA and 0.47 mM Pd^{2+} (2:1 [AA]/[Pd^{2+}] ratio). (A) First 20 minutes of ICP kinetics measurements and (B) full 180 minutes of ICP kinetics measurements.

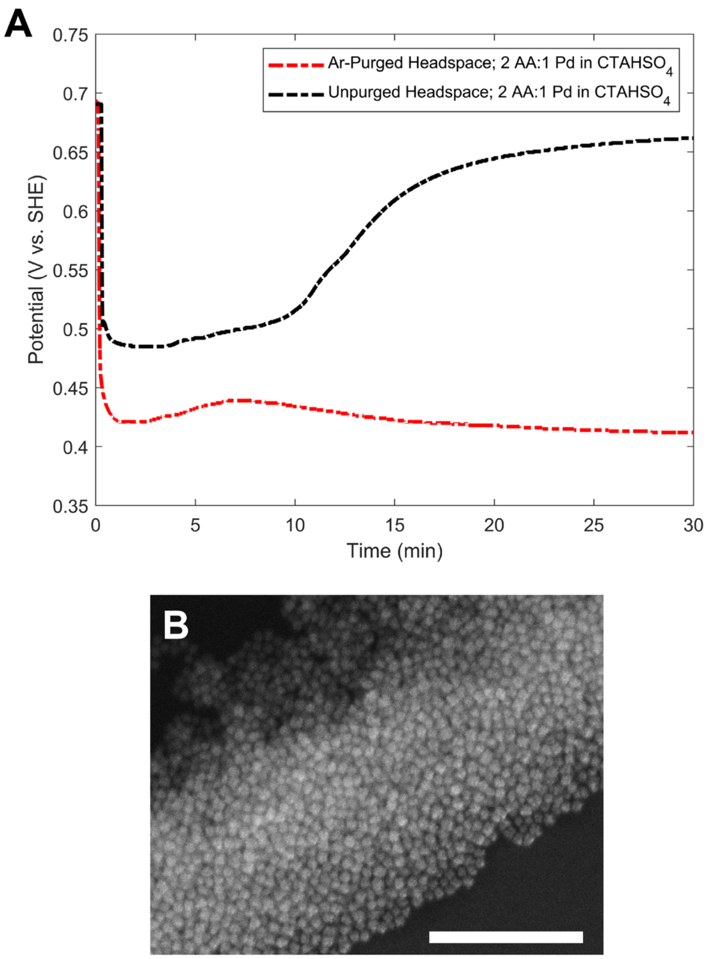

Figure S41. (A) OCP measurements of Pd particle growth in $CTAHSO_4$ with 0.94 mM AA and 0.47 mM Pd^{2+} (2:1 [AA]/[Pd^{2+}] ratio) under ambient conditions (black) and under argon (red). (B) SEM image of Pd particles grown under argon. Scale bar: 500 nm.

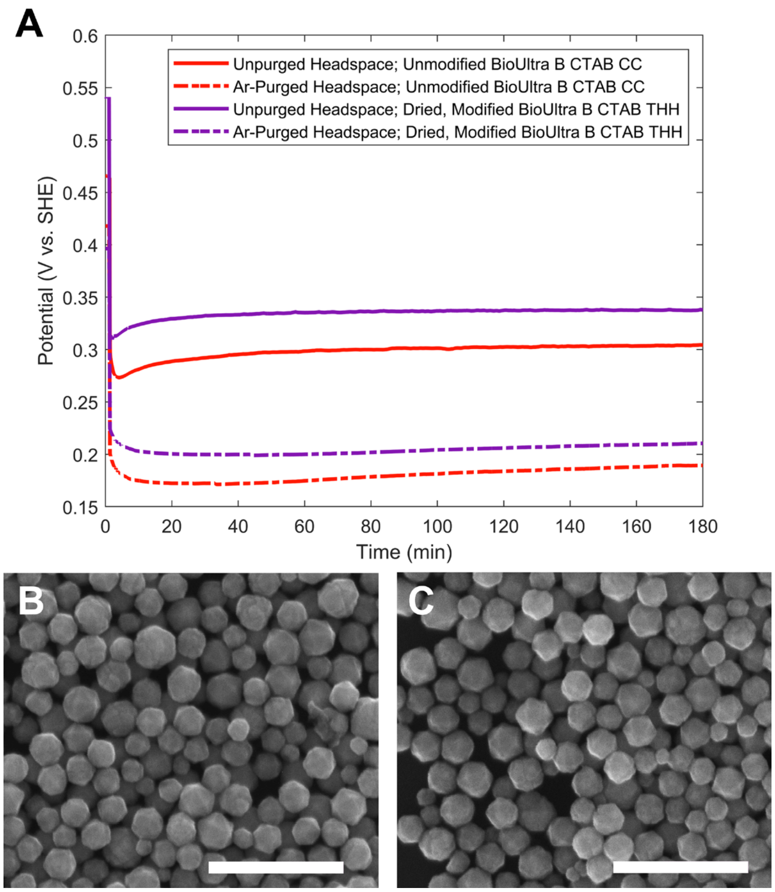

Figure S42. (A) OCP measurements of Pd CC (red) and THH (purple) growth in BioUltra B CTAB under ambient conditions (solid lines) and the same growth conditions under argon (dot-dash lines). (B) SEM image of polydisperse Pd THH grown under argon in as-received BioUltra B CTAB (typical CC-forming conditions). (C) SEM image of polydisperse Pd THH grown under argon in dried, modified BioUltra B CTAB (typical THH-forming conditions). Scale bar: 500 nm.

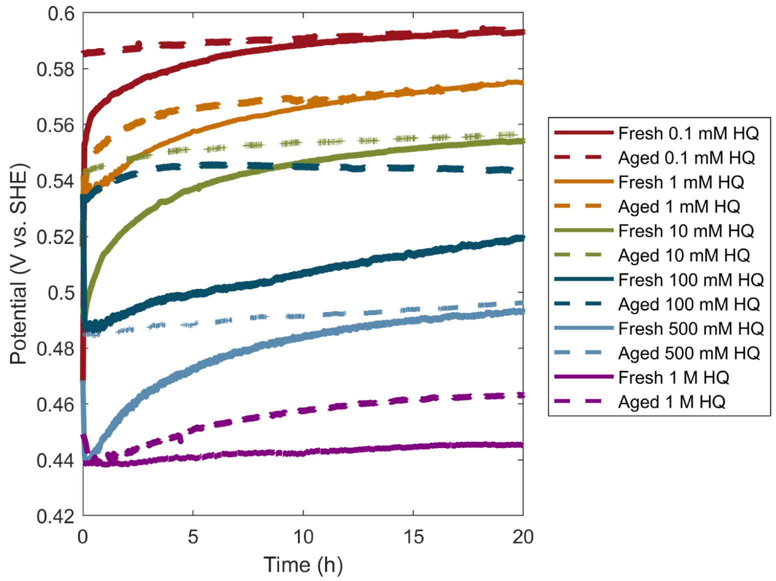

Figure S43. OCP measurements of fresh and aged HQ in acidic, aqueous solution. The potential before and after HQ degradation is [HQ]-dependent.

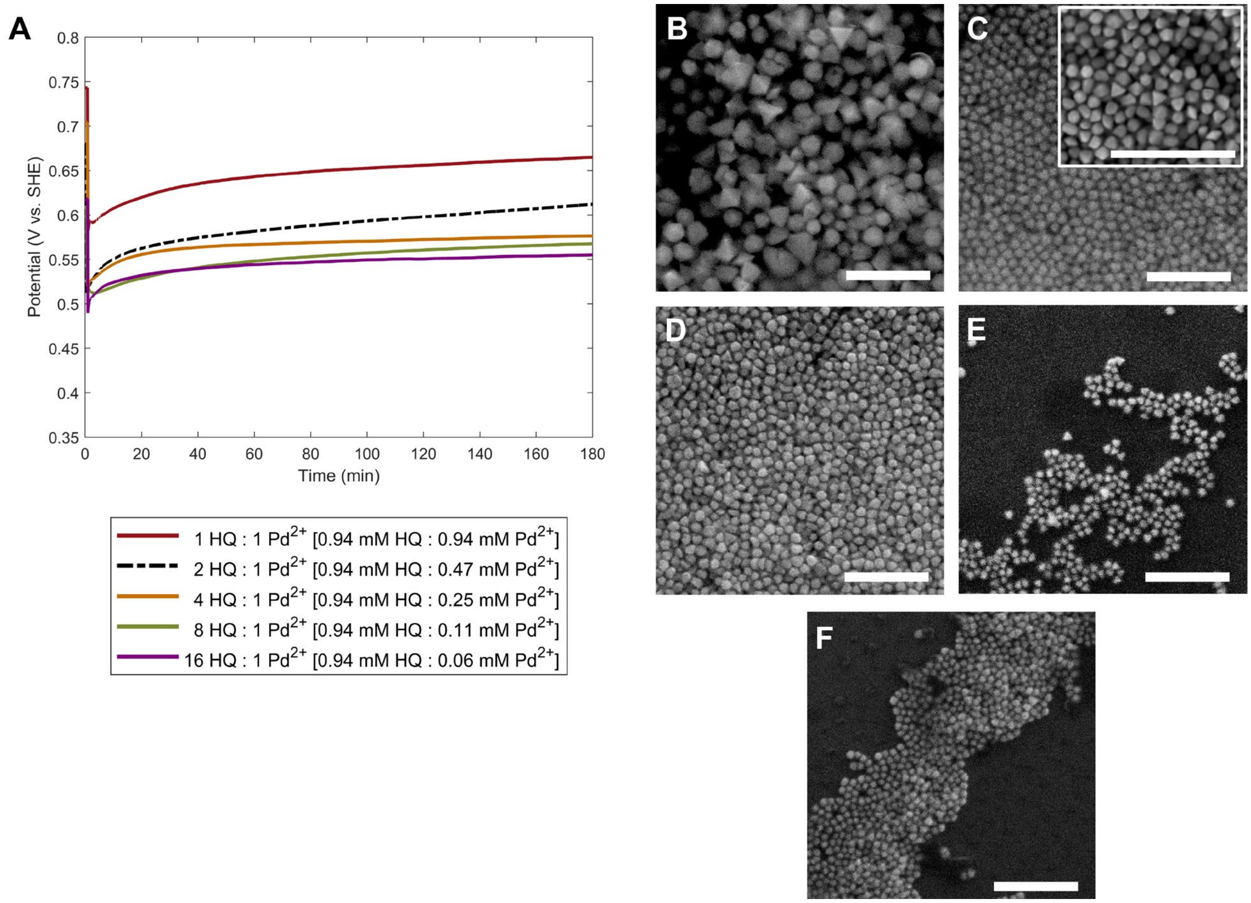

Figure S44. (A) OCP measurements of Pd particle growth solutions in $CTAHSO_4$ with varied ratios of [HQ]/[Pd^{2+}], with the ratio controlled by varying [Pd^{2+}] while [HQ] is fixed at 0.94 mM. (B-F) SEM images of Pd particle growth in $CTAHSO_4$ with a fixed 0.94 mM HQ concentration and (B) 0.94 mM Pd^{2+} (1:1 [HQ]/[Pd^{2+}] ratio), (C) 0.47 mM Pd^{2+} (2:1 [HQ]/[Pd^{2+}] ratio), (D) 0.25 mM Pd^{2+} (4:1 [HQ]/[Pd^{2+}] ratio), (E) 0.11 mM Pd^{2+} (8:1 [HQ]/[Pd^{2+}] ratio), and (F) 0.06 mM Pd^{2+} (16:1 [HQ]/[Pd^{2+}] ratio). Scale bars: 500 nm.

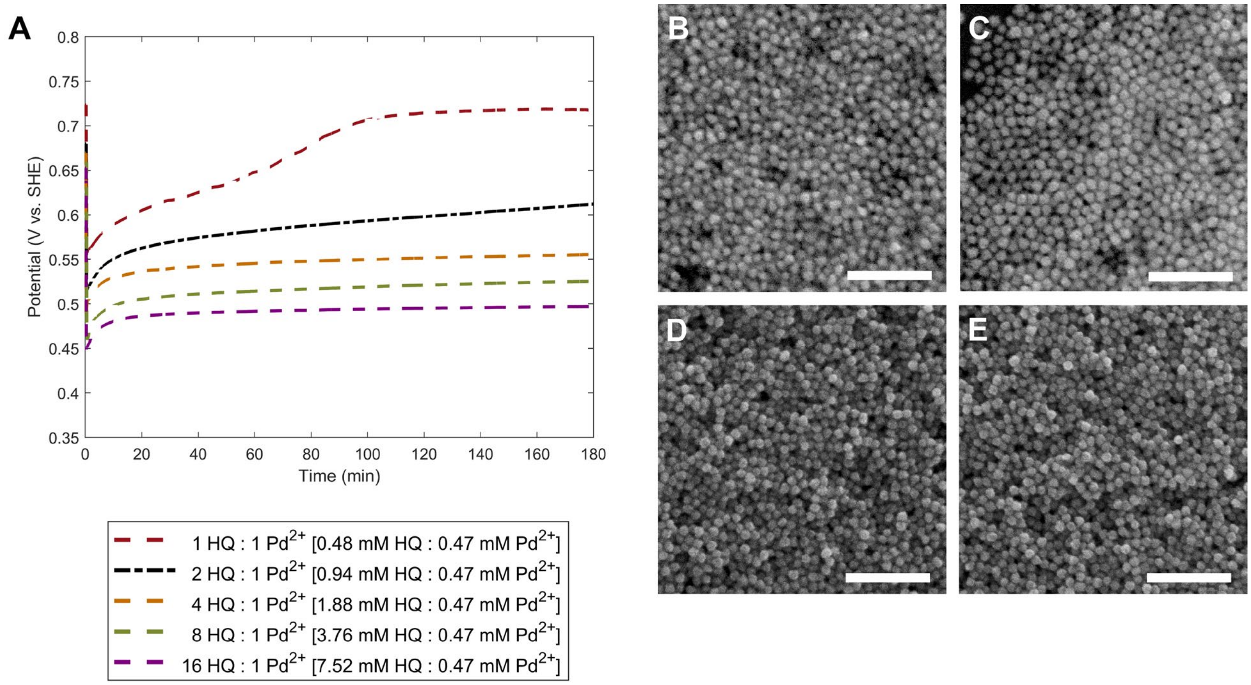

Figure S45. (A) OCP measurements of Pd particle growth solutions in $CTAHSO_4$ with varied ratios of [HQ]/[Pd^{2+}], with the ratio controlled by varying [HQ] while [Pd^{2+}] is fixed at 0.47 mM. (B-E) SEM images of Pd particle growth in $CTAHSO_4$ with a fixed 0.47 mM Pd^{2+} concentration and (B) 0.48 mM HQ (1:1 [HQ]/[Pd^{2+}] ratio), (C) 1.88 mM HQ (4:1 [HQ]/[Pd^{2+}] ratio), (D) 3.76 mM HQ (8:1 [HQ]/[Pd^{2+}] ratio), and (E) 7.52 mM HQ (16:1 [HQ]/[Pd^{2+}] ratio). See Figure S44C for SEM image of Pd particles with 2:1 [HQ]/[Pd^{2+}] ratio. Scale bars: 500 nm.

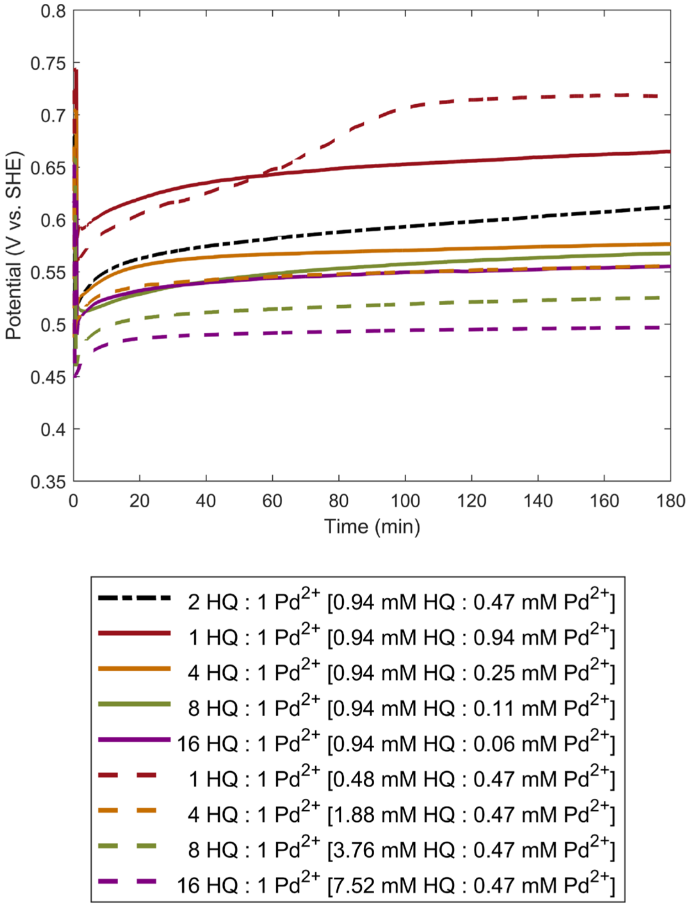

Figure S46. Comparison of OCP measurements of Pd nanoparticle synthesis reactions with different ratios of HQ to Pd precursor, with the ratio controlled by changing [Pd^{2+}] or [HQ].

References

(1) Halford, G. C.; McDarby, S. P.; Hertle, S.; Kiely, A. F.; Luu, J. T.; Wang, C. J.; Personick, M. L. Troubleshooting the Influence of Trace Chemical Impurities on Nanoparticle Growth Kinetics *via* Electrochemical Measurements. *Nanoscale* **2024**, *16*, 11038–11051. https://doi.org/10.1039/D4NR00070F.

(2) Reddy Satyavolu, N. S.; Peinetti, A. S.; Wang, Y.; Ali, A. S.; Lin, J. W.; Lu, Y. Silver-Assisted Synthesis of High-Indexed Palladium Tetrahexahedral Nanoparticles and Their Morphological Variants. *Chem. Mater.* **2019**, *31*, 2923–2929. https://doi.org/10.1021/acs.chemmater.9b00275.

(3) King, M. E.; Personick, M. L. Defects by Design: Synthesis of Palladium Nanoparticles with Extended Twin Defects and Corrugated Surfaces. *Nanoscale* **2017**, *9*, 17914–17921. https://doi.org/10.1039/C7NR06969C.

(4) Zhang, J.; Langille, M. R.; Personick, M. L.; Zhang, K.; Li, S.; Mirkin, C. A. Concave Cubic Gold Nanocrystals with High-Index Facets. *J. Am. Chem. Soc.* **2010**, *132*, 14012–14014. https://doi.org/10.1021/ja106394k.

(5) Liu, S.-Y.; Shen, Y.-T.; Chiu, C.-Y.; Rej, S.; Lin, P.-H.; Tsao, Y.-C.; Huang, M. H. Direct Synthesis of Palladium Nanocrystals in Aqueous Solution with Systematic Shape Evolution. *Langmuir* **2015**, *31*, 6538–6545. https://doi.org/10.1021/acs.langmuir.5b01337.

(6) McDarby, S. P.; Wang, C. J.; King, M. E.; Personick, M. L. An Integrated Electrochemistry Approach to the Design and Synthesis of Polyhedral Noble Metal Nanoparticles. *J. Am. Chem. Soc.* **2020**, *142*, 21322–21335. https://doi.org/10.1021/jacs.0c07987.

(7) Personick, M. L.; Langille, M. R.; Zhang, J.; Mirkin, C. A. Shape Control of Gold Nanoparticles by Silver Underpotential Deposition. *Nano Lett.* **2011**, *11*, 3394–3398. https://doi.org/10.1021/nl201796s.

(8) Roger, I.; Symes, M. D. Silver Leakage from Ag/AgCl Reference Electrodes as a Potential Cause of Interference in the Electrocatalytic Hydrogen Evolution Reaction. *ACS Appl. Mater. Interfaces* **2017**, *9*, 472–478. https://doi.org/10.1021/acsami.6b13438.

(9) Bard, A. J.; Faulkner, L. R.; White, H. S. *Electrochemical Methods: Fundamentals and Applications*; Wiley, 2022.

(10) Zhou, H.; Park, J. H.; Fan, F.-R. F.; Bard, A. J. Observation of Single Metal Nanoparticle Collisions by Open Circuit (Mixed) Potential Changes at an Ultramicroelectrode. *J. Am. Chem. Soc.* **2012**, *134*, 13212–13215. https://doi.org/10.1021/ja305573g.

(11) Halford, G. C.; Hertle, S.; N. Nambiar, H.; Personick, M. L. Using Electrochemistry to Benchmark, Understand, and Develop Noble Metal Nanoparticle Syntheses. *ACS Nanosci. Au* **2025**, *5*, 240-261. https://doi.org/10.1021/acsnanoscienceau.5c00051.

(12) Zimmermann, P.; Weltin, A.; Urban, G. A.; Kieninger, J. Active Potentiometry for Dissolved Oxygen Monitoring with Platinum Electrodes. *Sensors* **2018**, *18*, 2404. https://doi.org/10.3390/s18082404.

(13) Reimer, U.; Cai, Y.; Li, R.; Froning, D.; Lehnert, W. Time Dependence of the Open Circuit Potential of Platinum Disk Electrodes in Half Cell Experiments. *J. Electrochem. Soc.* **2019**, *166*, F3098–F3104. https://doi.org/10.1149/2.0121907jes.

(14) Liu, X.; MacDonald, D. D.; Wang, M.; Xu, Y. Effect of Dissolved Oxygen, Temperature, and pH on Polarization Behavior of Carbon Steel in Simulated Concrete Pore Solution. *Electrochim. Acta* **2021**, *366*, 137437. https://doi.org/10.1016/j.electacta.2020.137437.

(15) Sun, S.; Billings, A.; Wang, B.; Huang, K. Combined Effect of Dissolved Oxygen and pH in Aqueous Electrolytes on Zn-Anode Corrosion Behavior in Aqueous Zn-Ion Batteries. *ACS Electrochem.* **2025**, *1*, 195–204. https://doi.org/10.1021/acselectrochem.4c00086.
(16) Percival, S. J.; Bard, A. J. Ultra-Sensitive Potentiometric Measurements of Dilute Redox Molecule Solutions and Determination of Sensitivity Factors at Platinum Ultramicroelectrodes. *Anal. Chem.* **2017**, *89*, 9843–9849. https://doi.org/10.1021/acs.analchem.7b01856.
(17) Parkhutik, V.; Rayon, E.; Pastor, E.; Matveeva, E.; Sasano, J.; Ogata, Y. Study of Oscillatory Behavior of Open-circuit Potential of Silicon Immersed in $CuSO_4$ /HF Solutions. *Phys. Status Solidi (a)* **2005**, *202*, 1586–1591. https://doi.org/10.1002/pssa.200461192.
